# Monitoring Radical Intermediates in Photoactivated Palladium-Catalyzed Coupling of Aryl Halides to Arenes by an Aryl Radical Assay

**DOI:** 10.1021/acscatal.4c06913

**Published:** 2024-12-30

**Authors:** Seb Tyerman, Donald G. MacKay, Kenneth F. Clark, Alan R. Kennedy, Craig M. Robertson, Laura Evans, Robert M. Edkins, John A. Murphy

**Affiliations:** a Department of Pure and Applied Chemistry, University of Strathclyde, 295 Cathedral Street, Glasgow G1 1XL, United Kingdom; b GSK Medicines Research Centre, Gunnels Wood Road, Stevenage, Herts SG1 2NY, United Kingdom; c Medicinal Chemistry, Research and Early Development, Oncology R&D, AstraZeneca, Cambridge CB10 1XL, United Kingdom

**Keywords:** palladium, coupling, photoactivation, radical, assay, arene, biaryl

## Abstract

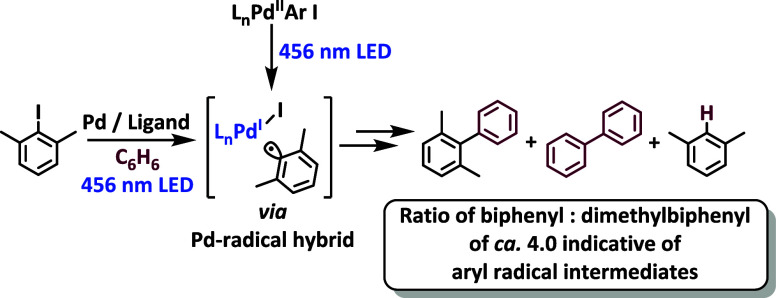

An aryl radical assay is used to provide information about the formation of aryl radicals from aryl halides in coupling reactions to arenes in the presence of palladium sources and under LED irradiation (λ = 456 nm). The assay uses 2-halo-*m*-xylenes as substrates. Aryl radical formation is indicated both by a defined product composition and by signature deuterium isotope effects. Comparison with our recently published results for corresponding ground-state palladium-catalyzed reactions shows three principal differences: (i) in the photoactivated reactions, evidence supports the formation of aryl radical intermediates with all the phosphine ligands tested, in contrast to thermal ground-state chemistry where only specific ligands had encouraged this pathway, while others had promoted a nonradical coupling mechanism; (ii) oxidative addition complexes that are formed from the reaction of Pd(0) sources with aryl halides react under photoactivation to form biaryl coupled products through radical intermediates, in contrast to their behavior under thermal activation – so Ar–Pd bonds are homolyzed under LED irradiation; (iii) the photoreactions work well with mild bases like Cs_2_CO_3_, while the thermal reactions required KO^t^Bu as the base due to the different roles for base under the thermal versus photochemical mechanisms.

## Introduction

**Scheme 1:**
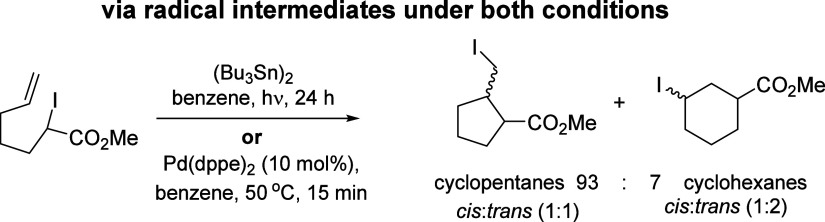
Cyclization Effected under Radical Conditions OR under Palladium Activation Gives Identical Outcome

Catalysis with palladium salts and complexes is an immensely valuable tool for organic chemists. While the majority of these transformations are proposed to proceed through traditional Pd^0^/Pd^II^ catalytic cycles, there are notable exceptions.^1−5^ In some cases, palladium promotes the formation of organic radicals in the ground state, and this was perhaps best illustrated by Curran and Chang,^1^ where the outcome of the atom-transfer reaction with tributyltin radicals was compared to that from thermal reaction with Pd(dppe)_2_ in benzene (Scheme 1). The product distribution between 5-membered and 6-membered ring products was the same under both reaction conditions and the ratio of *cis*:*trans* stereoisomers was the same within both the cyclopentanes and the cyclohexanes, giving a multipoint fingerprint that provided convincing support that the same radical intermediates governed both reactions.

As early as 1985, it was known that common Pd^0^–phosphine complexes absorb light in the visible region and possess long-lived excited triplet states,^6,7^ but in the past decade, visible light-driven palladium catalysis has drawn significant attention.^8−13^ With palladium complexes acting as photocatalysts that harvest photons and subsequently reacting through their excited triplet states for bond-breaking or bond-forming steps, these transformations are quite distinct from those employing non-Pd-based photocatalysts.^14−16^ Reports in this area have primarily focused on the activation of organic halides, principally alkyl halides, fueled by the inherent problems of employing these substrates using ground-state palladium catalysis, namely, slow oxidative addition rates and facile β-hydride elimination from Pd^II^-alkyl intermediates. Visible light has also been shown to accelerate other aspects of palladium catalysis such as reductive elimination^17^ and reduction of the Pd^II^ precatalyst.^18^

**Scheme 2:**
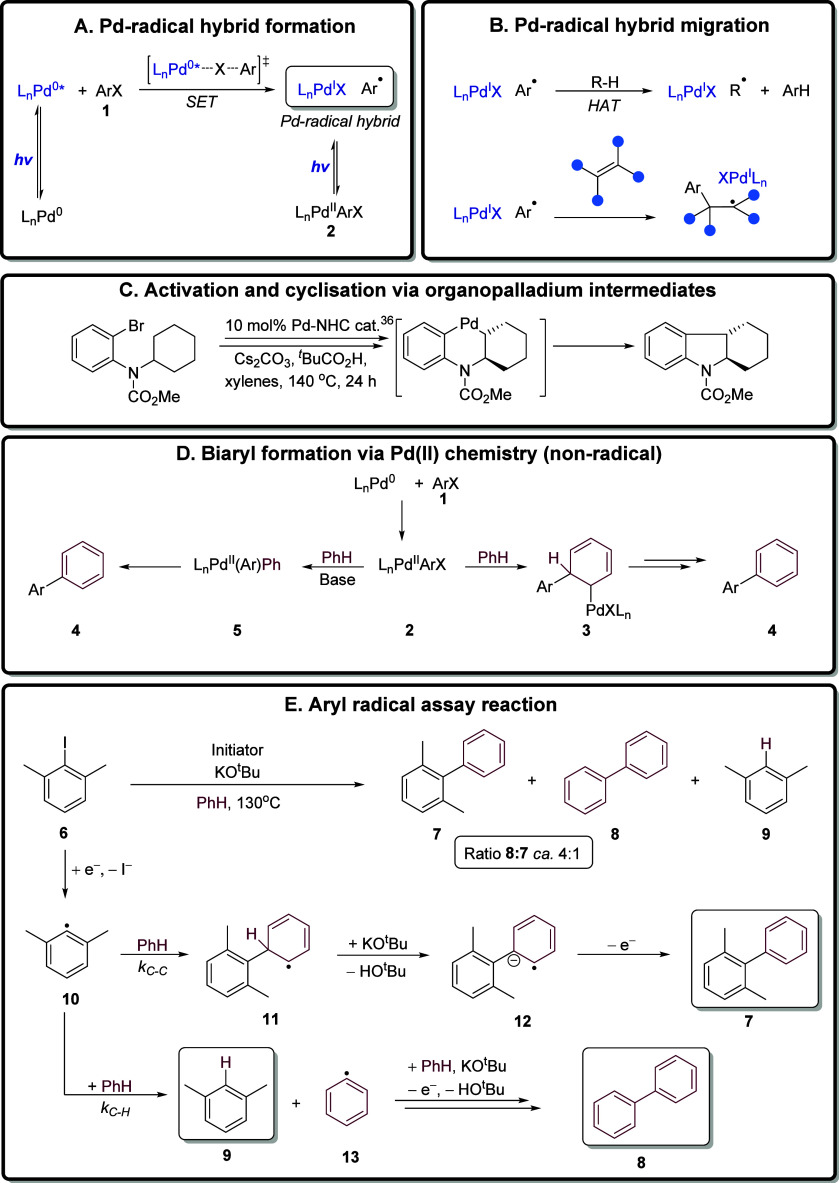
Radical and Nonradical Chemistry of Aryl Halides Mediated by Pd Complexes

Reactions of aryl halides have also been widely studied. Since Gevorgyan’s seminal 2016 report on the remote desaturation of silyl ethers,^19^ many groups have employed aryl halides **1** using photoexcited palladium catalysis for intramolecular^19−26^ and intermolecular transformations.^17,27−31^ A common reaction pathway is proposed by most groups in which a Pd^0^ species is promoted to its excited triplet state Pd^0^* via intersystem crossing following visible-light irradiation to generate the initial excited singlet state (Scheme 2A). This Pd^0^* species then undergoes oxidative quench with the aryl halide either via single-electron transfer (SET) to the organohalide or by halogen atom transfer (XAT),^30^ forming a palladium-radical hybrid species. The radical hybrid is interpreted as an equilibrium between the formal oxidative addition complex L_n_Pd^II^ArX **2** and the separated L_n_Pd^I^ metalloradical and organic radical fragments under irradiation. Once formed, the radical can react through a variety of pathways, such as hydrogen atom transfer (HAT) or addition to unsaturated species, resulting in migration of the Pd-radical hybrid (Scheme 2B).

For alkyl halides, a barrierless single electron transfer (SET) to form the hybrid species has been elucidated by computation,^32−34^ but such calculations are absent for aryl halides. Indirect evidence for the Pd-aryl radical hybrid is provided, based on tests for radical intermediates such as radical traps like TEMPO,^19,21^ cyclization reactions and ring-opening reactions,^19,27^ hydrogen transfer reactions, and patterns of deuterium labeling in products.^19,22,28^ Taken together, the evidence supports radical intermediates, but chemists are rightly cautious in determining the mechanism. Traps such as TEMPO are most informative when they lead to isolated trapped products, but often, they are observed to just inhibit a reaction. This can indicate the presence of radicals, but TEMPO can also modify the nature of metal-based reactions.^35^ Cyclization reactions and transfer reactions can indicate radical processes but these transformations^36−38^ and ring-opening reactions^39−41^ are also associated with nonradical chemistry of palladium (see Scheme 2C for an example involving transfer and cyclization).

The development of the recent radical-based palladium chemistry contrasts with traditional nonradical-based Pd coupling reactions. For example, coupling of aryl halides **1** with arenes mediated by Pd complexes without intervention of radicals leads to formation of biaryls and this can occur by a number of routes (Scheme 2D). Oxidative addition of an aryl halide **1** to form a Pd(II) complex **2** could in principle lead to addition to an arene to form **3**(42) followed by rearomatization to biaryl **4**. Alternatively, concerted metalation-deprotonation (CMD) could generate a complex **5** that affords the biaryl product **4** through reductive elimination.^43^

Returning to radical chemistry, aryl radicals are exceptionally reactive species and thus are difficult to identify directly as reaction intermediates.^44^ We recently reported an assay reaction using a special substrate to identify aryl radicals unambiguously.^45^ When aryl radicals are produced, the assay provides very strong mechanistic evidence through two channels – (i) a diagnostic ratio of two coupled products is observed (as is outlined in the paragraph below), and (ii) the isotope effects seen in deuterated versus nondeuterated solvent strongly either support or refute radical mechanisms (this will be discussed later in this paper). In this way, this assay provides multipoint evidence that can support or refute evidence for aryl radicals.

Using 2,6-dimethyliodobenzene **6** as the substrate with potassium *tert*-butoxide (KO^t^Bu) as the base and benzene as the solvent, together with any reagent that can convert some **6** into radicals **10**, this leads to products 2,6-dimethylbiphenyl **7**, biphenyl **8** and *m*-xylene **9**(45) via a base-promoted homolytic aromatic substitution (BHAS) reaction (Scheme 2E).^46^ Once **6** is converted to a xylyl radical **10** by an initiator, it can react by addition to benzene (*k*_C–C_); after deprotonation of the resulting cyclohexadienyl radical **11**, oxidation of the corresponding radical anion **12** yields product **7**. Alternatively, due to steric hindrance imposed by the *ortho*-methyl groups, **10** may abstract a hydrogen atom from benzene (*k*_C–H_), yielding *m*-xylene **9** and a phenyl radical **13**, which subsequently adds to benzene yielding biphenyl **8**. (Hydrogen abstraction can also occur from other sources, notably benzylic methyl groups). A diagnostic ratio of ca. 4:1 of **8:7** is found when aryl radicals are produced under our thermal conditions, resulting from the ratio of the rate constants *k*_C–C_ and *k*_C–H_.^45^

This assay was recently applied by us to the thermally activated coupling of haloarenes with benzene mediated by palladium complexes in the presence of base.^47^ A number of parameters were examined but, taking as an example results with Pd(OAc)_2_ as the palladium source and KO^t^Bu as the base, addition of a range of ligands including ferrocene-based diphosphine ligands such as dppf (see Table 2 for structure) strongly promoted a radical BHAS mechanism (ratio of **8**:**7** = 4:1) while other ligands, e.g., PCy_3_ (ratio of **8**:**7** = 1:46) opted almost completely for nonradical coupling. In nonradical Pd chemistry, product **8** would not be produced. In this Letter, we now employ this assay to investigate the formation of aryl radicals by excited-state palladium catalysis. The aim is to provide a rapid multipoint test of whether defined conditions (for palladium source, ligands, base, photoactivation) lead to conversion of aryl halides to aryl radicals.

## Results and Discussion

### Effect of Base

Initially Pd(OAc)_2_ and Xantphos were employed in our assay reaction [0.35 mmol **6**, 2 equiv. KO^t^Bu, 2.5 mL C_6_H_6_, 456 nm LED, 24 h], which, besides affording xylene **9**, yielded coupled products **8** and **7** in a ratio of 6.5:1 (Table 1, entry 1), a notably higher ratio than had been observed under thermal conditions.^47^ However, subsequent control reactions showed that KO^t^Bu in combination with Xantphos or other ligands (entry 2 and SI), in the absence of palladium, can initiate coupling under irradiation. This additional photoactivated pathway makes KO^t^Bu unsuitable for photoexcited palladium couplings in our assay reaction.

**Table 1:**
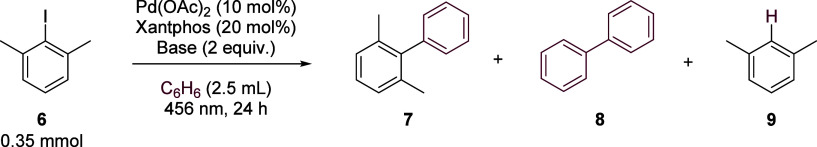
Effect of the Base

			**% yield**				
**entry**	**base**	**conditions changed**	**6**	**7**	**8**	**9**	**ratio 8:7**
1	KO^t^Bu		0.3	3.1	20.2	51.0	6.5
2	KO^t^Bu	no Pd	27.7	2.1	16.1	44.5	7.7
3	NaO^t^Bu		0.0	5.1	26.1	58.6	5.1
4	K_3_PO_4_		17.3	4.6	21.0	51.7	4.5
5	Cs_2_CO_3_		10.8	4.3	21.2	54.0	5.0
6	KOAc		85.5	1.3	6.5	13.5	5.0
7	no base		85.9	1.5	6.4	13.8	4.2
8	Cs_2_CO_3_	no Pd	96.2	0.2	0.5	3.4	2.4
9	Cs_2_CO_3_	no Xantphos	99.6	0.1	0.0	0.3	
10	Cs_2_CO_3_	60 °C, no light	98.2	0.7	0.3	0.9	0.3
11^a^	Cs_2_CO_3_	no **6**			2.0		
12^b^	Cs_2_CO_3_	2-bromo-*m*-xylene instead of **6**	18.3	4.0	19.8	40.3	4.9
13^c^	Cs_2_CO_3_	2-chloro-*m*-xylene instead of **6**	90.2	0.6	3.7	4.9	6.4

a 0.0071 mmol produced, 2.0% refers to a comparison with reactions employing 0.35 mmol of 2-iodo-*m*-xylene.

b 18.3% 2-bromo-*m*-xylene unconsumed.

c 90.2% 2-chloro-*m*-xylene unconsumed.

**Table 2:**
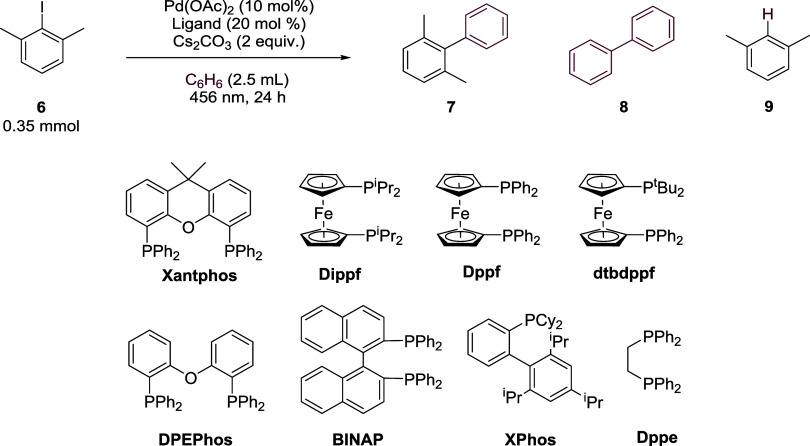
Ligand Screen

		**% yield**				
**entry**	**ligand**	**6**	**7**	**8**	**9**	**ratio 8:7**
1	Xantphos	10.8	4.3	21.2	54.0	5.0
2	DPEPhos	30.0	2.5	10.1	44.0	4.1
3	Dippf	0.1	3.0	14.4	64.7	4.8
4	Dppf	0.0	3.1	14.5	58.1	4.6
5	Dtbdppf	0.9	3.5	14.6	50.2	4.2
6	BINAP	15.1	2.9	14.7	55.6	5.0
7	XPhos	8.9	3.3	11.9	50.1	3.6
8	Dppe	34.8	1.4	7.3	35.8	5.3
9	PPh_3_ (40 mol %)	17.6	3.0	12.0	49.4	4.0
10	PCy_3_ (40 mol %)	28.2	2.0	7.6	46.7	3.8
11	P^t^Bu_3_	58.5	0.9	2.2	15.6	2.5

Alternative bases as weak as Cs_2_CO_3_^19,25^ provided coupled products in improved yields (entries 3–5), which is notable as, under thermal conditions, only stronger potassium alkoxide bases such as KO^t^Bu can promote BHAS coupling (*vide infra*). However, KOAc is too weak to facilitate the photoreaction (entry 6) and provides yields similar to those of a reaction in the absence of base (entry 7). The reaction in the absence of base facilitates a ca. 15% conversion of **6** into products, indicating that Cs_2_CO_3_ and related bases have a role to play that is different than the role of base in BHAS,^46^ and most likely involves regeneration of Pd^0^, as discussed later. Control reactions show that both ligands and Pd(OAc)_2_ are needed to facilitate coupling (entries 8–9). Reactions were measured to reach 60 °C during irradiation without cooling, but a reaction at 60 °C in the absence of irradiation provided only trace amounts of products (entry 10). In a reaction in the absence of haloarene **6**, small quantities of biphenyl were still observed (entry 11). Repeating the reaction in benzene-*d*_6_ afforded unlabeled biphenyl and confirmed the ligand phenyl groups as the origin of this product (see SI Table S1, entry 15). Exchanging **6** for 2-bromo-*m*-xylene did not significantly hinder the reaction (entry 12), while 2-chloro-*m*-xylene proved to be inefficient in this system (entry 13). Recently, Maiti et al. reported cross electrophile coupling (XEC) between (hetero)aryl halides enabled by visible light-induced palladium catalysis;^31^ however, homocoupling was not observed under our conditions, likely due to the steric hindrance of **6** and its derivatives.

### Ligand and Pd Source Screen

A survey of ligands was then carried out using Pd(OAc)_2_ as the range of ligands routinely employed in visible-light-promoted palladium reactions is relatively small compared to that of the ground state. DPEPhos, a structural relative of Xantphos, also facilitated radical coupling, albeit in lower yields (Table 2, entry 2). A range of ferrocene-based ligands and BINAP (entries 3–6), all of which initiated BHAS under ground-state conditions with KO^t^Bu,^47^ as well as bulky XPhos (entry 7), also produced ratios of **8:7** that were consistent with radical processes. It is notable that dippf, which bears no phenyl groups on the phosphine, also provides a high **8:7** ratio of 4.8. Interestingly, when ligands that under thermal conditions promoted nonradical coupling were employed (dppe, PPh_3_ and PCy_3_, entries 8–10), ratios were now observed that supported a radical mechanism, highlighting an entirely different reactivity under irradiation. Tertiary alkyl phosphine P^t^Bu_3_ was not very effective in the reaction (entry 11).^48^

Alternative palladium sources were explored (Table 3). As expected in the absence of phosphine ligands, Pd_2_dba_3_ or PdCl_2_ did not facilitate the reaction (entries 1–2). When either Xantphos or BINAP was added to either of these Pd sources (entries 3–5), effective couplings were seen, similar to those obtained in Table 2 using Pd(OAc)_2_, indicating a similar Pd active catalyst. Employing Pd(PPh_3_)_4_ in the reaction (entry 6) provided results comparable to those using Pd(OAc)_2_/4PPh_3_ (Table 2, entry 9), while (PPh_3_)_2_PdCl_2_ alone was quite ineffective (entry 7). Addition of Xantphos to either of these Pd sources (entries 8–9) as a mixed-ligand system (i.e., combination of bidentate and monodentate phosphines) led to significantly higher yields, but slightly lower **8:7** ratios.^33,49^

**Table 3:**
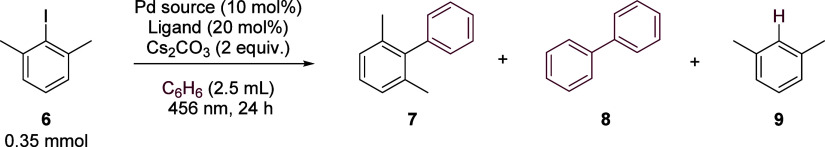
Coupling Reactions Using a Range of Pd Sources

			**% yield**				
**entry**	**Pd**	**ligand**	**6**	**7**	**8**	**9**	**ratio8:7**
1	Pd_2_dba_3_		98.2	0.0	0.0	0.9	
2	PdCl_2_		98.8	0.6	0.1	0.6	0.1
3	Pd_2_dba_3_	Xantphos	1.0	5.6	22.0	55.9	3.9
4	Pd_2_dba_3_	BINAP	0.0	3.6	17.3	62.1	4.8
5	PdCl_2_	Xantphos	15.1	5.0	23.4	53.8	4.7
6	Pd(PPh_3_)_4_		8.9	3.2	14.9	55.7	4.7
7	(PPh_3_)_2_PdCl_2_		77.9	1.0	4.2	15.2	4.2
8	Pd(PPh_3_)_4_	Xantphos	0.2	7.0	22.7	60.8	3.2
9	(PPh_3_)_2_PdCl_2_	Xantphos	0.7	6.6	26.2	61.1	3.9

### Kinetic Isotope Effects

**Scheme 3:**
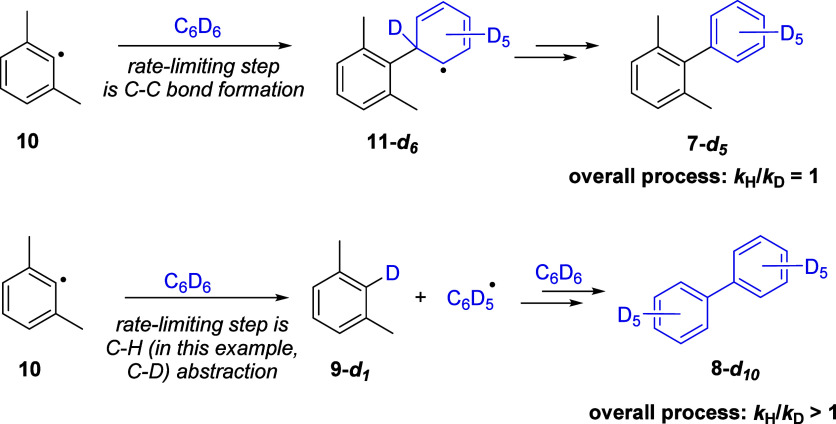
Kinetic Isotope Effects for Attack of the Xylyl Radical on Benzene or HAT by the Xylyl Radical

An important aspect of the assay is the effect of carrying out the reaction in deuterated solvent (Scheme 3).^47^ If the xylyl radical attacks benzene or, in this case, benzene**-***d*_6_ (*k*_C–C_) to form **11-***d*_**6**_ in a rate-determining step (i.e., if the subsequent conversion to **7** or, in this case, **7-***d*_**5**_ including the cleavage of the C–D bond is not rate limiting^44,46^), then carrying out the reaction with benzene-*d*_*6*_ would not incur a primary KIE and thus no fall in the yield of **7-***d*_**5**_ (relative to the yield of **7** when C_6_H_6_ was the solvent) will be seen. However, abstraction of a hydrogen atom by xylyl radical **10** from benzene (*k*_C–H_) to form xylene **9** and phenyl radical **13** would incur a large KIE when C_6_D_6_ is used, leading to a fall in the yield of **9-***d*_**1**_ and, consequently, a fall in the yield of **8-***d*_**10**_ (relative to **8** when the reaction is carried out in C_6_H_6_). If **7** is instead formed via an organometallic pathway such as CMD, a large KIE effect would be expected for formation of **7**.^43^

For all palladium/ligand combinations tested (Table 4), replacing benzene with benzene-*d*_*6*_ led to no change, or indeed a slight increase, in the yield of **7**. This was accompanied by a significant fall in the yield of **8**, and **9** to a lesser extent. This is consistent with the formation of a xylyl radical from **6**. The lesser fall in the yield of **9**, and the fact that it remains predominantly unlabeled (deuterium incorporation between 2.5% and 7.2%) has been explored in our previous ground-state studies with this assay and is due to the large concentration of reactive benzylic C–H bonds providing a ready source of hydrogen atoms.^45,50^ In entries 2, 4 and 6, while the major coupled products in all cases are **7-***d*_**5**_ and **8-***d*_**10**_, there are traces of **7**, **8-***d*_**5**_ and **8** arising from incorporation of the phenyl groups on the ligands (see SI for details). In the case of dippf, however, **7** and **8** were entirely **7-***d*_**5**_ and **8-***d*_**10**_ (entry 8).

**Table 4:**
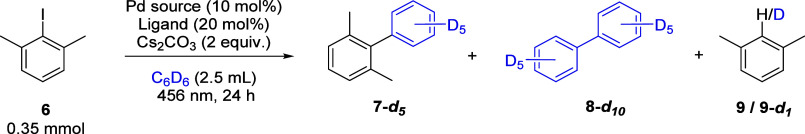
Effect of Deuterated Solvent on the Reaction

				**% yield**						
**entry**	**Pd**	**ligand**	**solvent**	**6**	**7**	**7-***d*_**5**_	**8**	**8-***d*_**10**_	9/9**-***d*_**1**_	**ratio 8:7**
1^a^	Pd(PPh_3_)_4_		C_6_H_6_	8.9	3.2		14.9		55.7	4.7
2	Pd(PPh_3_)_4_		C_6_D_6_	17.9		3.2		1.7	39.9	0.5
3^b^	Pd(OAc)_2_	Xantphos	C_6_H_6_	10.8	4.3		21.2		54.0	5.0
4	Pd(OAc)_2_	Xantphos	C_6_D_6_	15.1		4.3		1.9	39.6	0.4
5^c^	Pd(OAc)_2_	Dppf	C_6_H_6_	0.0	3.1		14.5		58.1	4.6
6	Pd(OAc)_2_	Dppf	C_6_D_6_	0.2		4.0		2.4	43.3	0.6
7^d^	Pd(OAc)_2_	Dippf	C_6_H_6_	0.1	3.0		14.4		64.7	4.8
8	Pd(OAc)_2_	Dippf	C_6_D_6_	0.0		3.0		1.2	46.1	0.4

a Results duplicated from Table 3, entry 6.

b Results duplicated from Table 1, entry 5.

c Results duplicated from Table 2, entry 4.

d Results duplicated from Table 2, entry 3.

### Oxidative Addition Complexes as Substrates

Carrow et al. recently reported that, under visible light irradiation, T-shaped alkyl-Pd^II^ complexes such as (P^t^Bu_3_)Pd^II^MeCl underwent homolysis, liberating alkyl radicals in addition to a Pd^I^ metalloradical fragment which could be trapped by TEMPO.^51^ When studying the carbonylation of aryl halides, Arndtsen et al. found that irradiation of proposed intermediate (DPEPhos)Pd^II^(COAr)(Cl) by visible light produced the acyl chloride (via the acyl radical) and Pd^0^ nearly quantitatively, a reaction that reversed in the dark.^17^ To our knowledge, direct homolysis of Pd^II^–Ar bonds under visible light-irradiation has not been reported.^52^ In our previous study investigating ground-state palladium chemistry, stoichiometric reactions of oxidative addition intermediates did not provide coupled products when using a ligand (dppf) that successfully promoted radical coupling from corresponding aryl halides, showing that the oxidative addition species are not intermediates in the ground-state radical coupling reactions.^47^ We now employed oxidative addition complexes in stoichiometric reactions to test for homolysis of the Pd^II^–Ar bond under visible light.

Stoichiometric reactions were carried out using the *cis-*oxidative addition complexes (dppf)Pd(xylyl)(I) **14a** and (BINAP)Pd(xylyl)(I) **14b** as well as *trans-*complexes (PPh_3_)_2_Pd(xylyl)(I) **14c**, (PCy_3_)_2_Pd(xylyl)(I) **14d**, and (P^i^Pr_3_)_2_Pd(xylyl)(I) **14e**, all of which were simply prepared from Pd_2_dba_3_, ligand, and **6**. Under standard conditions, both *cis-*complexes **14a** and **14b** provided coupled products **7** and **8** in good yields relative to the starting complex and importantly exhibited a ratio of **8:7** (Table 5, entries 1–2) that supported a radical mechanism. *Trans-*complex **14c** also produced high yields, although with a slightly lower ratio of **8:7** (entry 3). Complexes bearing alkylphosphine ligands, **14d** and **14e**, produced coupled products in much lower yields in addition to significant quantities of reductive elimination product **6**, but a ratio of **8:7** supporting aryl radical intermediates was maintained (entries 4–5). When deuterated benzene, C_6_D_6_, was employed, a large decrease in the yield of **8** and **9** was observed, accompanied by a significant increase in the yield of **7** (entries 6–8). Additionally, the proportion of **9** that is deuterated (deuterium incorporation 65.2% from **14a**, 46.9% from **14b**, and 78.8% from **14c**) is much higher than in catalytic reactions carried out in C_6_D_6_ (Table 4) because the concentration of benzyl C–H groups is much lower. These results are consistent with homolysis of the Pd–Ar bond to form an *m*-xylyl radical (**10**). Interestingly, the base could be excluded with little or no detriment to the reactions (entries 9–10). Examination of the same substrates in blank reactions, i.e., in the absence of light (but heating to 60 °C to mimic the temperature attained in the photoactivated conditions), led to very low yields of coupled products **7** and **8**, thereby confirming the key role of irradiation in the conversions of the oxidative addition complexes to coupled products (see SI file).

**Table 5:**
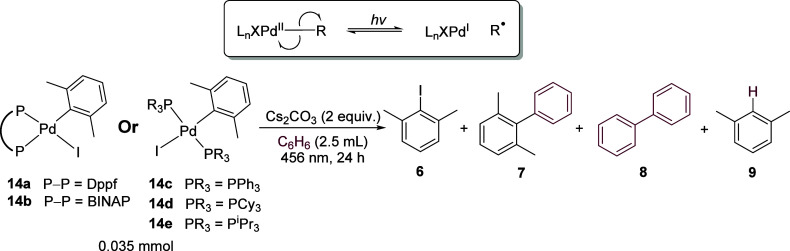
Reactions of Oxidative Addition Products L_n_Pd(xylyl)(I)

			**% yield**				
**entry**	**complex**	**conditions changed**	**6**	**7**	**8**	**9**	**ratio 8:7**
1	**14a**		0.0	15.1	72.0	83.0	4.8
2	**14b**		2.0	10.8	42.9	64.1	4.0
3	**14c**		7.2	15.7	45.8	61.1	2.9
4	**14d**		26.2	2.3	9.0	54.1	3.9
5	**14e**		23.6	3.6	12.2	74.3	3.4
6	**14a**	C_6_D_6_	0.0	52.2	30.5	42.7	0.6
7	**14b**	C_6_D_6_	4.2	26.4	10.9	41.0	0.4
8	**14c**	C_6_D_6_	3.4	38.5	13.6	37.5	0.4
9	**14a**	no base	0.0	15.9	72.3	80.9	4.8
10	**14b**	no base	4.8	9.0	42.1	60.8	4.7

### Mechanistic Investigations

A number of factors in this study contrast with our earlier assay results under ground-state conditions. The most obvious of these is the versatility of base that can be employed, whereby under photoactivation radical reactivity is not limited to reactions using potassium alkoxide bases such as KO^t^Bu. Additionally, the base is not required for high yields in stoichiometric reactions with oxidative addition complexes. These findings indicate that products are not generated by a BHAS chain reaction, and instead, the base is involved in regenerating Pd^0^ from HPd^II^X intermediates as part of a catalytic cycle. This proposal aligns more closely with literature precedent.^19,53^ Potassium alkoxide bases are thought to be essential in BHAS chemistry due to (i) high basicity to deprotonate the cyclohexadienyl radical intermediate and (ii) favorable ion-π interactions between the potassium cation and haloarenes to lower reduction potentials. We did consider whether photoexcitation might increase acidity of intermediates to aid deprotonation and/or increase the reducing power of electron donors, allowing alternative weaker bases, like Cs_2_CO_3_, to facilitate coupling under a BHAS chain mechanism. To investigate this, a biphenyl radical anion (the type of electron donor involved in propagation of a BHAS cycle) was generated by the reaction of 4,4′-di-*tert*-butylbiphenyl (DDTB) **15** with 1 equiv of potassium metal in THF, generating a dark blue/green solution indicative of formation of the radical anion **16**.^54^

The **16**/THF solution was then added as an initiator (ca. 17 mol %) to the assay reaction under a range of conditions (Table 6). When added to a reaction using KO^t^Bu as the base at 130 °C, i.e., our normal thermal conditions under which BHAS is expected to cycle, consumption of **6** was ca. 45%, indicating cycling of the chain reaction (entry 1). Although the major product was **9**, as expected from the introduction of THF (bearing weak OC–H bonds) to the reaction mixture, yields of **7** and **8** were higher than those in the absence of the initiator.^47,55^ When added to a reaction with Cs_2_CO_3_ as the base either under thermal conditions (entry 2) or photoexcited conditions (entry 3), consumption of **6** was ca. 23%, which, within error, does not indicate propagation of a BHAS cycle. Accordingly, any role for Cs_2_CO_3_ under photoexcitation as a base in a BHAS chain mechanism can be ruled out.

**Table 6:**
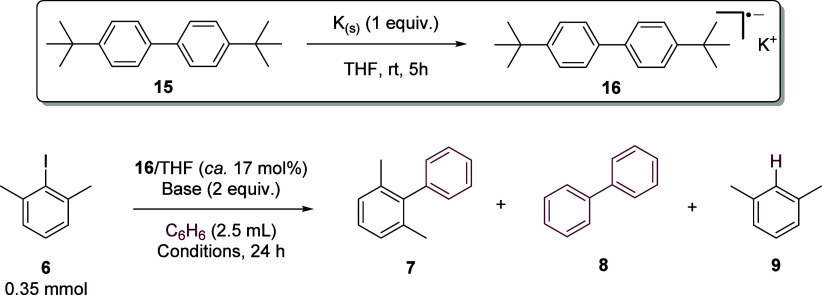
Using the Biphenyl Radical Anion to Probe for the Chain Reaction

			**% yield**			
**entry**	**base**	**conditions**	**6**	**7**	**8**	**9**
1	KO^t^Bu	130 °C	54.7	0.6	1.2	43.1
2	Cs_2_CO_3_	130 °C	77.1	0.1	0.1	18.7
3	Cs_2_CO_3_	456 nm	76.9	<0.1	0.2	20.1

**Figure 1:**
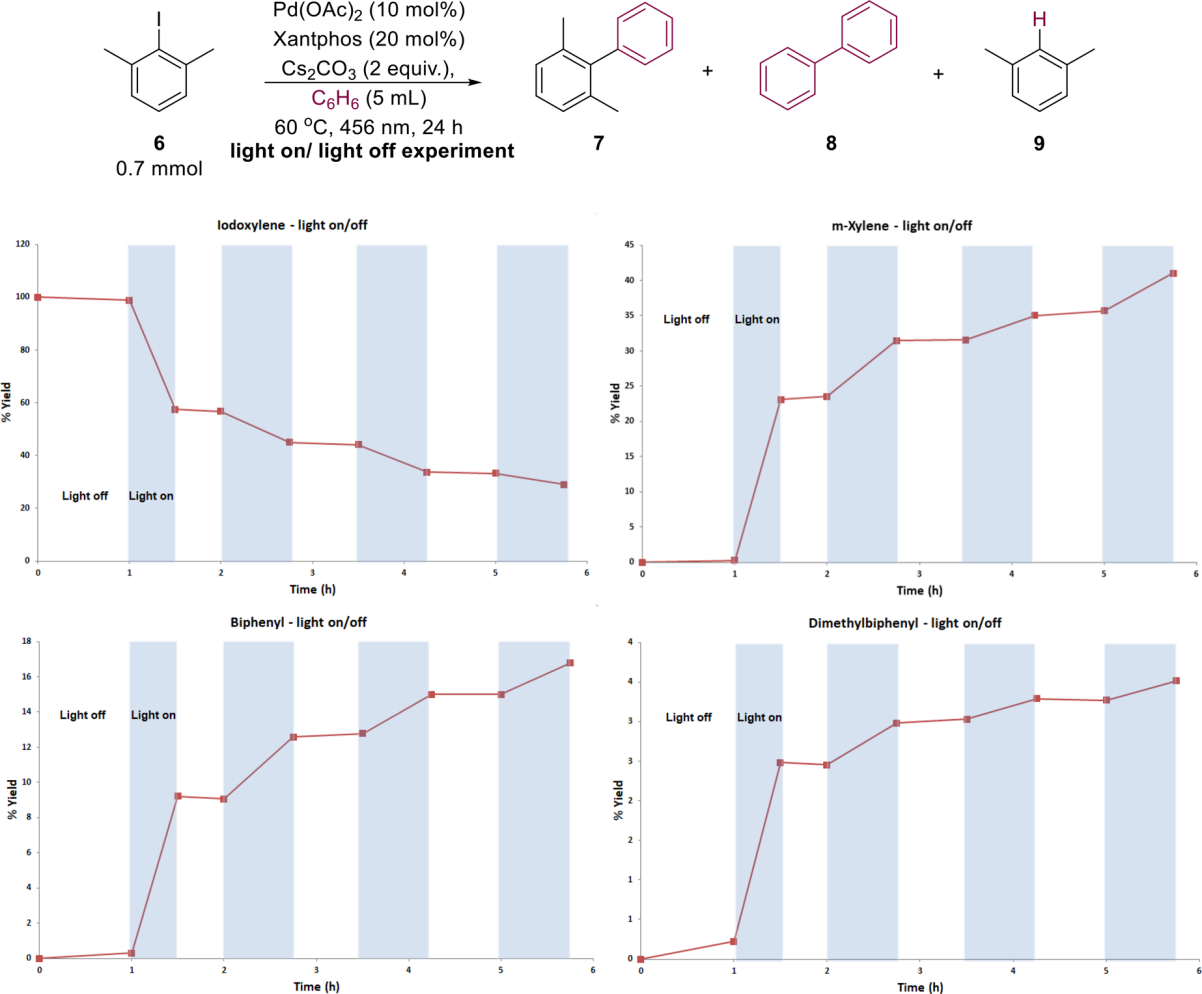
Light on/off experiment showing that irradiation is needed to progress the reaction.

A light on/off experiment was also carried out, using Pd(OAc)_2_ (10 mol %), Xantphos (20 mol %), Cs_2_CO_3_ (2 equiv), and benzene, showing stepwise formation of all three products during light-on cycles, suggesting that a chain reaction was not involved. (Figure 1). We also investigated the quantum yield of the process (Φ = 0.26),^56^ which supported this proposal (see SI file).

**Scheme 4:**
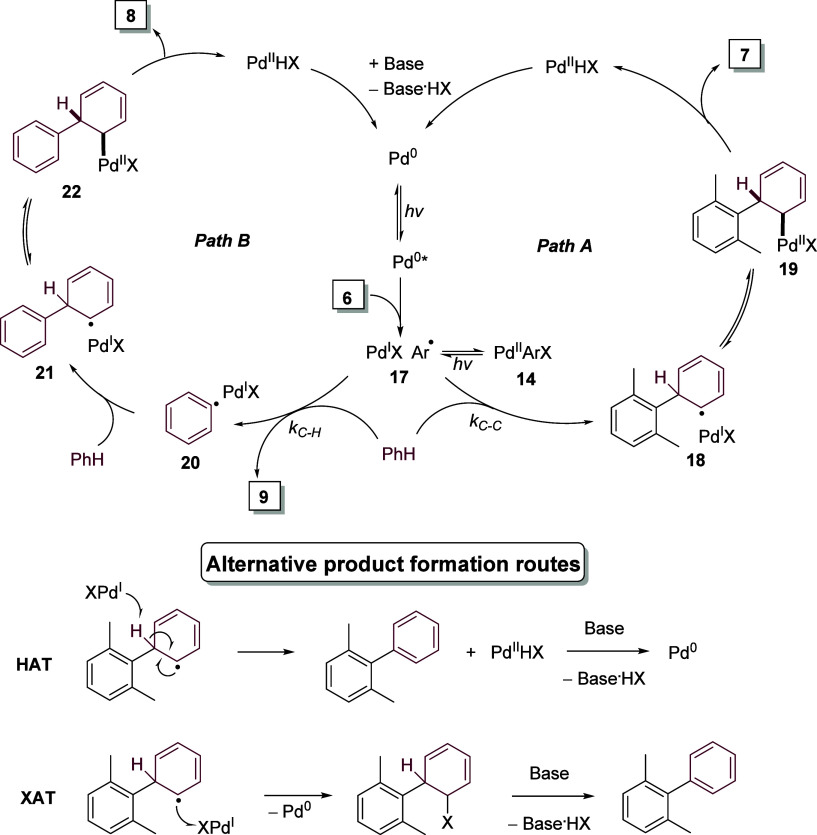
Proposed Mechanism

Based on these experiments and supported by Stern–Volmer quenching measurements (see SI file), it is likely that the reaction of aryl halides with arenes under visible light-induced palladium catalysis is facilitated through a Pd^0^/Pd^I^/Pd^II^ cycle, a plausible mechanism for which is outlined in Scheme 4.^19^ Initially, a Pd^0^ species generated *in situ* is photoexcited to Pd^0^*. This then undergoes SET with iodoxylene **6** yielding the Pd-radical hybrid **17**, which then has two paths of reactivity. In Path A, the radical attacks the π* system of benzene, forming cyclohexadienyl radical hybrid **18**, which reversibly recombines with Pd^I^ to form **19**, and after β-hydride elimination yields dimethylbiphenyl **7** and Pd^II^HX, from which base regenerates Pd^0^. Alternatively, through Path B, **17** undergoes HAT with benzene forming xylene **9** and a new radical hybrid **20**, which can attack benzene and through recombination/β-hydride elimination yields biphenyl **8**. However, there are multiple possibilities for the rearomatization of the cyclohexadienyl radicals **18**/**21** to products by palladium and alternative pathways should be considered, such as direct HAT by Pd^I^ or XAT and subsequent base-assisted elimination.^19^

## Conclusions

In conclusion, an assay has been employed to investigate the formation of aryl radicals using visible light-induced palladium catalysis for the coupling of aryl halides to arenes. Under photoexcitation, the assay confirms that various palladium sources and ligand combinations produce aryl radicals from aryl halides. The presence of radicals is confirmed both by diagnostic ratios of products **8:7** and by comparison of the outcomes of the reactions in deuterated versus undeuterated solvent. Additionally, LED irradiation of the formal oxidative addition complexes, L_n_Pd^II^ArI, is shown to produce aryl radicals. These findings highlight the ability of the assay to provide multipoint mechanistic information. The results of these photoactivated reactions contrast our previous study of ground-state palladium chemistry involving formation of biaryls from aryl halides and arenes under basic (BHAS) conditions.^47^ Under ground-state conditions, products are produced via a BHAS chain reaction requiring a strong base, while in photoactivated mode, a mild base instead promotes a Pd^0^/Pd^I^/Pd^II^ catalytic cycle, supporting other reports on the reactivity of Pd-radical hybrid species.^8−13^ This and related assays are currently being applied to other reaction types.^50^

## Supporting Information Available

Experimental procedures, spectroscopic and crystallographic information (PDF)
