# Supplementary material for: Monitoring Radical Intermediates in Photoactivated Palladium-Catalyzed Coupling of Aryl Halides to Arenes by an Aryl Radical Assay
Source: ACS Catal. 2024 Dec 30;15(2):917–27. doi: 10.1021/acscatal.4c06913 (PMC11744670; doi:10.1021/acscatal.4c06913)
Supplement: Supplementary file 1 — cs4c06913_si_001.pdf [file cs4c06913_si_001.pdf]

Monitoring Radical Intermediates in Photoactivated Palladium-Catalysed Coupling of Aryl Halides to Arenes by an Aryl Radical Assay

Seb Tyerman, [a] Donald G. MacKay, [a] Kenneth F. Clark, [a] Alan R. Kennedy, [a] Craig M. Robertson, [b] Laura Evans, [c] Robert M. Edkins [a] and John A. Murphy* [a]

[a]Department of Pure and Applied Chemistry, University of Strathclyde, 295 Cathedral Street, Glasgow G1 1XL. [b]GSK Medicines Research Centre, Gunnels Wood Road, Stevenage, Herts SG1 2NY, United Kingdom. [c]Medicinal Chemistry, Research and Early Development, Oncology R&D, AstraZeneca, Cambridge CB10 1XL, U.K.

email: john.murphy@strath.ac.uk

Contents | Page number

1.0	General information	S2
2.0	General procedures	S3
3.0	Conditions screen	S4
4.0	Ligand screen	S5
5.0	Pd source screen	S6
6.0	Reactions of different 2-halo-1,6-dimethylbenzenes	S6
7.0	Reactions in deuterated solvents	S7
8.0	Synthesis of oxidative addition complexes L_nPd(xylyl)(I)	S9
9.0	$Pd(Xantphos)_2$	S12
10.0	Reactions of oxidative addition complexes L_nPd(xylyl)(I)	S15
11.0	UV-Visible absorption spectra of oxidative addition complexes	S20
12.0	Stern-Volmer quenching studies	S21
13.0	Testing for a chain reaction	S23
14.0	NMR spectra	S30
15.0	X-ray data	S34
16.0	Calibration curves	S36
17.0	GC-FID traces	S41
18.0	References	S77

1.0 General information

All reactions were carried out using air-free techniques under argon or prepared in a glovebox (Innovative Technology Inc., USA) under a nitrogen atmosphere and sealed, unless otherwise stated. All reagents were obtained from commercial suppliers (Fluorochem, Fisher Scientific, Apollo Scientific, or Merck) and used without further purification, unless otherwise stated. Anhydrous toluene, tetrahydrofuran (THF) and dichloromethane (DCM) were dried using a Pure-Solv 400 solvent purification system (Innovative Technology Inc., USA), while anhydrous benzene was purchased from Merck and used as received. Thin Layer Chromatography was performed on silica gel pre-coated aluminium plates (60 Å, F254 UV indicator) purchased from Merck. The thin layer chromatograms were analysed by UV (254 nm, UVP mineralight UVG-11 lamp) and staining either with basic $KMnO_4$ solution [$KMnO_4$ (6 g), K_2CO_3 (40 g), NaOH (5 mL, 10% w/w) in water (600 mL)] or an ethanolic solution of phosphomolybdic acid [phosphomolybdic acid hydrate (10 g) in ethanol (100 mL)]. Flash Column Chromatography purification was performed with 40-63 µm particle size silica gel 60 Å purchased from Fluorochem. NMR spectroscopy was performed using a Bruker AV3-400 spectrometer. ^{1}H NMR, ^{13}C NMR and ^{31}P NMR spectra were recorded on this spectrometer operating at 400 MHz, 101 MHz, 162 MHz respectively. All spectral data were acquired at 295 K. For ^{1}H and ^{13}C NMR spectra, chemical shifts (δ) are quoted in parts per million (ppm) relative to the following residual solvent peaks; δ_H 7.26 and δ_C 77.2 ppm for $CDCl_3$, δ_H 7.16 and δ_C 128.0 ppm for C_6D_6, δ_{H1} 1.72 ppm and δ_{H2} 3.58 ppm for THF-d_8. ^{31}P NMR chemical shifts are referenced externally to H_3PO_4 at 0.0 ppm in D_2O. $CDCl_3$, C_6D_6 and THF-d_8 were purchased from Eurisotop and used as received. Coupling constants (J) are reported in Hertz (Hz) to the nearest 0.1 Hz. The multiplicity abbreviations used are: s (singlet), d (doublet), t (triplet), q (quartet), m (multiplet), br (broad). GCMS spectra were obtained on an Agilent 7890A GC system coupled to a 5975C inert XL EI/CI MSD triple axis-mass detector. Electron impact (EI) ionisation was utilised, specifically the method EI320-10-1-SPLIT. The column temperature was 320 °C, and the carrier gas was helium with a flow rate of 1 mL/min. GC-FID analyses were carried out using an Agilent 7890A gas chromatograph fitted with an Agilent HP5 column (30 m x 0.25 mm x 0.25 µm). Helium was used as the carrier gas (2.0 mL/min flow rate). The maximum column temperature was 340 °C and the instrument was operated in a splitless mode. UV–visible absorption spectra were recorded at room temperature using an Agilent Cary 60 spectrophotometer. Baseline corrections were achieved by reference to pure solvent. Emission spectra were recorded using a Cary Eclipse spectrometer operating in right-angle geometry mode, and all spectra were fully corrected for the spectral response of the instrument. All solutions used in fluorescence measurements had an absorbance below 0.2 at the excitation wavelength to minimize inner filter, unless stated otherwise.

2.0 General procedures

General Procedure A – Assay reaction

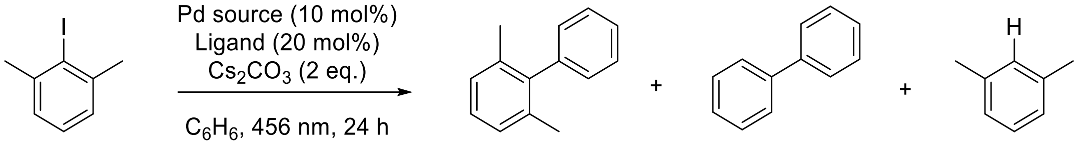

6 **7** **8** **9**

Unless otherwise stated, reactions were carried out as follows. An oven-dried 10 mL microwave vial was charged with 2,6-dimethyliodobenzene **6** (50.5 μL, 0.35 mmol, 1 eq.), a palladium source (0.035 mmol, 0.1 eq.), a ligand (0.07 mmol, 0.2 eq.), finely ground Cs_2CO_3 (228.1 mg, 0.7 mmol, 2 eq.) (or other base, as specified), anhydrous benzene (2.5 mL) and a clean magnetic stirbar. The vial was capped, removed from the glovebox and stirred under irradiation from a 456 nm Kessil lamp placed 2 cm away for 24 hours (reactions were measured to reach 60°C using an infrared thermometer). The reaction was then allowed to cool to room temperature and uncapped, after which an accurately weighed amount of *n*-dodecane internal standard was added. The reaction mixtures were then filtered through a short pad of celite to remove insolubles and washed with *n*-hexane and EtOAc. A small aliquot of the reaction mixture was diluted in EtOAc and submitted for analysis by GC-FID, where the yields of products were determined by calibration of the instrument with authentic samples *vs*. *n*-dodecane (see page 31 for calibration information). % Yields are reported with respect to 2,6-dimethyliodobenzene **6** (0.35 mmol).

General Procedure B – Stoichiometric reactions from complexes $L_nPd(xylyl)(I)$ (14)

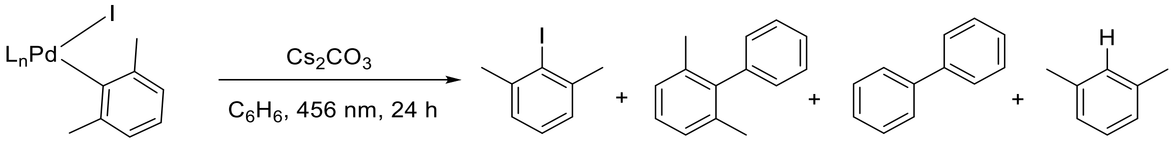

14 **6** **7** **8** **9**

Unless otherwise stated, reactions were carried out as follows. An oven-dried 10 mL microwave vial was charged with the palladium complex **14** (0.035 mmol, 1 eq.), finely ground Cs_2CO_3 (22.8 mg, 0.07 mmol, 2 eq.), anhydrous benzene (2.5 mL) and a clean magnetic stirbar. The vial was capped, removed from the glovebox and stirred under irradiation from a 456 nm Kessil lamp placed 2 cm away for 24 h (reactions were measured to reach 60°C using an infrared thermometer). The reaction was then allowed to cool to room temperature and uncapped, after which an accurately weighed amount of *n*-dodecane internal standard was added. The reaction mixtures were then filtered through a short pad of celite to remove insolubles and washed with *n*-hexane and EtOAc. A small aliquot of the reaction mixture was diluted in EtOAc and submitted for analysis by GC-FID, where the yields of products were determined by calibration of the instrument with authentic samples vs. *n*-dodecane (see page 31 for calibration information). % Yields are reported with respect to the palladium complex **14** (0.035 mmol).

3.0 Condition screen

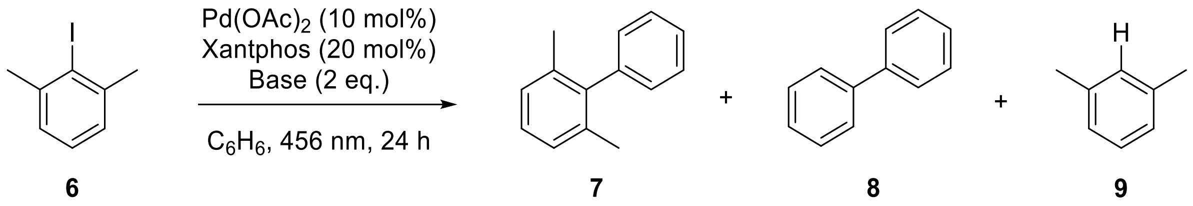

These reactions were carried out according to General Procedure A using $Pd(OAc)_2$ (7.9 mg, 0.035 mmol, 0.1 eq.) and Xantphos (40.5 mg, 0.07 mmol, 0.2 eq.), unless otherwise stated.

Entry	Base	Conditions changed	Dodecane (mg)	% Yield				Ratio 8:7
				6	7	8	9	
1	KO^tBu	-	10.7	0.3	3.1	20.2	51.0	6.5
2	KO^tBu	No $Pd(OAc)_2$	10.8	27.7	2.1	16.1	44.5	7.7
3	KO^tBu	C_6D_6, No $Pd(OAc)_2$,	10.5	47.4	1.9	1.4	29.4	1.5
4[a]	KO^tBu	PCy_3, No $Pd(OAc)_2$,	11.3	39.5	1.2	10.6	40.0	9.1
5	KO^tBu	No $Pd(OAc)_2$, No Xantphos	10.0	97.4	0.1	0.2	0.6	2.6
6	NaO^tBu	-	10.1	0.0	5.1	26.1	58.6	5.1
7	K_3PO_4	-	10.1	17.3	4.6	21.0	51.7	4.5
8	Cs_2CO_3	-	9.8	10.8	4.3	21.2	54.0	5.0
9	KOAc	-	10.8	79.9	1.3	6.5	14.9	5.0
10	No base	-	10.5	85.9	1.5	6.4	13.8	4.2
11	Cs_2CO_3	No $Pd(OAc)_2$	10.8	96.2	0.2	0.5	3.4	2.4
12	Cs_2CO_3	No Xantphos	10.2	99.6	0.1	0.0	0.3	-
13	Cs_2CO_3	60°C, no light	10.7	98.2	0.7	0.3	0.9	0.3
14[b]	Cs_2CO_3	No iodoxylene	10.4	-	-	2.0	-	-
15[c]	Cs_2CO_3	No iodoxylene, in C_6D_6	10.3	-	-	2.1	-	-

Table S1 – *[a]significant amounts of Cy-Ph produced. [b]0.0071 mmol.[c]0.0072 mmol*

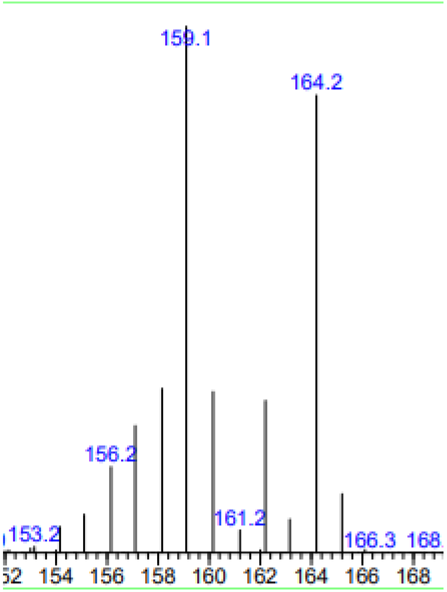

Figure S1 *– Mass spectrum for biphenyl* ***8*** *produced in reaction of iodoxylene/Xantphos/KO^tBu /C_6D_6 (Table S1, entry 3). A large proportion of the biphenyl is C_6H_5– C_6D_5 (m/z = 159), arising from incorporation of a Ph (C_6H_5) group of Xantphos. C_6D_5– C_6D_5 (m/z = 164.)*

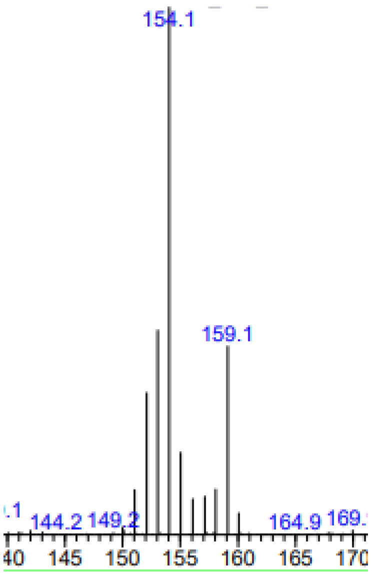

Figure S2 *– Mass spectrum for biphenyl **8** produced in reaction of Pd(OAc)$_2$/Xantphos/Cs_2CO_3/C_6D_6 in the absence of iodoxylene (Table S1, entry 15). The deuteration profile shows that here biphenyl arises from the Ph groups of Xantphos. C_6H_5– C_6H_5 (m/z = 154).*

4.0 Ligand screen

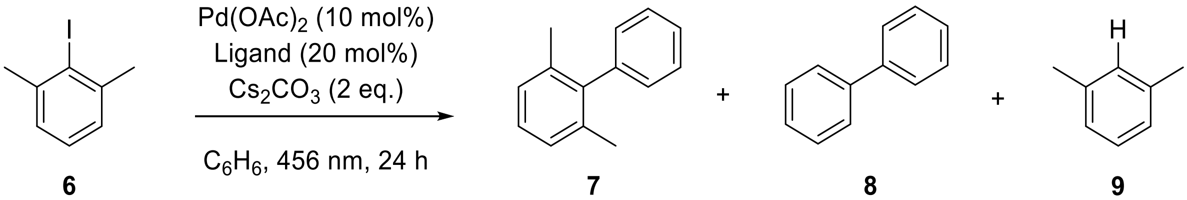

6 **7** **8** **9**

These reactions were carried out according to General Procedure A using Pd(OAc)$_2$ (7.9 mg, 0.035 mmol, 0.1 eq.) and a ligand (20 mol%), unless otherwise stated.

Entry	Pd	Ligand	Dodecane (mg)	% Yield				Ratio
				6	7	8	9	8:7
1	Pd(OAc)$_2$	Xantphos	9.8	10.8	4.3	21.2	54.0	5.0
2	Pd(OAc)$_2$	Xantphos (10 mol%)	8.6	54.1	2.9	10.0	28.3	3.4
3	Pd(OAc)$_2$	DPEPhos	9.0	30.0	2.5	10.1	44.0	4.1
4	Pd(OAc)$_2$	Dippf	9.2	0.1	3.0	14.4	64.7	4.8
5	Pd(OAc)$_2$	Dppf	8.8	0.0	3.1	14.5	58.1	4.6
6	Pd(OAc)$_2$	dtbdppf	9.3	0.9	3.5	14.6	50.2	4.2
7	Pd(OAc)$_2$	BINAP	9.2	15.1	2.9	14.7	55.6	5.0
8	Pd(OAc)$_2$	Dppe	9.7	34.8	1.4	7.3	35.8	5.3
9	Pd(OAc)$_2$	PPh_3	9.6	71.4	2.2	4.1	18.6	1.9
10	Pd(OAc)$_2$	PPh_3 (40 mol%)	9.8	17.6	3.0	12.0	49.4	4.0
11	Pd(OAc)$_2$	PCy_3	9.4	62.1	1.6	3.5	21.6	2.2
12	Pd(OAc)$_2$	PCy_3 (40 mol%)	11.0	28.2	2.0	7.6	46.7	3.8
13	Pd(OAc)$_2$	XPhos	9.2	8.9	3.3	11.9	50.1	3.6
14	Pd(OAc)$_2$	XPhos (40 mol%)	8.5	0.0	2.6	11.0	58.9	4.2
15	Pd(OAc)$_2$	P^tBu_3	9.6	58.5	0.9	2.2	15.6	2.5

Table S2 –

5.0 Pd source screen

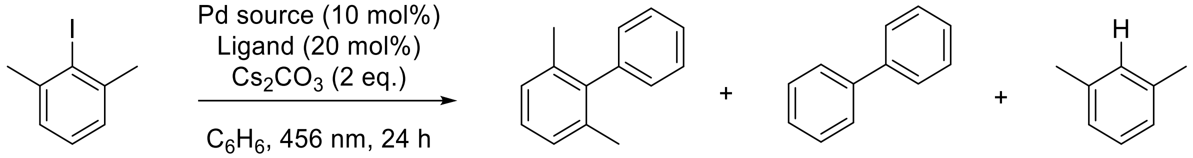

6 **7** **8** **9**

These reactions were carried out according to General Procedure A unless otherwise stated. Where Pd_2dba_3 was used, 5 mol% (10 mol% Pd) was used.

Entry	Pd	Ligand	Dodecane (mg)	% Yield				Ratio 8:7
				**6**	**7**	**8**	**9**	
1	Pd_2dba_3	-	11.1	98.2	0.0	0.0	0.9	-
2	$PdCl_2$	-	10.7	98.8	0.6	0.1	0.6	0.1
3	Pd_2dba_3	Xantphos	10.9	1.0	5.6	22.0	55.9	3.9
4	Pd_2dba_3	BINAP	10.4	0.0	3.6	17.3	62.1	4.8
5	$PdCl_2$	Xantphos	10.6	15.1	5.0	23.4	53.8	4.7
6	$Pd(PPh_3)_4$	-	10.2	8.9	3.2	14.9	55.7	4.7
7	$(PPh_3)_2PdCl_2$	-	10.6	77.9	1.0	4.2	15.2	4.2
8	$Pd(PPh_3)_4$	Xantphos (10 mol%)	10.9	0.2	7.0	22.7	60.8	3.2
9	$(PPh_3)_2PdCl_2$	Xantphos (10 mol%)	10.8	0.7	6.6	26.2	61.1	3.9

Table S3 –

6.0 Reactions of different 2,6-dimethylhalobenzenes

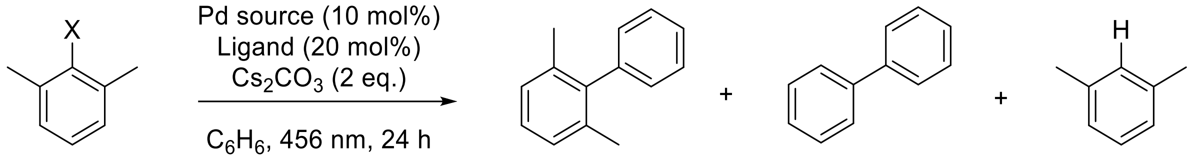

X = Br or Cl **7** **8** **9**

These reactions were carried out according to General Procedure A using $Pd(OAc)_2$ (7.9 mg, 0.035 mmol, 0.1 eq.), Xantphos (40.5 mg, 0.07 mmol, 0.2 eq.) and either 2-bromoxylene (46.6 μL, 0.35 mmol, 1 eq.) or 2-chloroxylene (46.4 μL, 0.35 mmol, 1 eq.).

Entry	Haloarene	Dodecane (mg)	% Yield				Ratio 8:7
			**haloarene**	**7**	**8**	**9**	
1	2-Bromoxylene	10.6	18.3	4.0	19.8	40.3	4.9
2	2-Chloroxylene	10.6	90.2[a]	0.6	3.7	4.9	6.4

Table S4 – *[a]calculated by ^1H NMR with a 1,1,2,2-tetrachloroethane (TCE) internal standard.*

7.0 Reactions in deuterated solvents

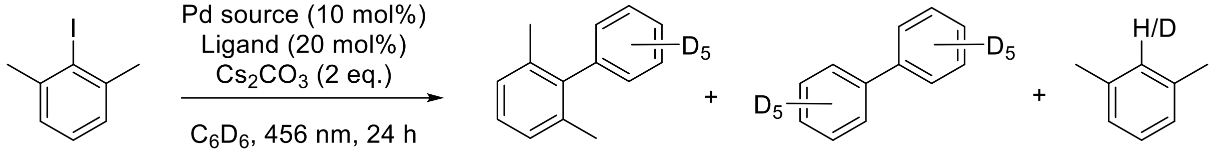

6 **7-*d*$_5$** **8-*d*$_{10}$** **9**

These reactions were carried out according to General Procedure A, except deuterated benzene C_6D_6 was employed as solvent (2.5 mL). Yields were determined by GC-FID, while deuteration of the products was determined by GC-MS.

Entry	Pd	Ligand	Dodecane (mg)	% Yield				Ratio
				**6**	**7-*d*$_5$**	**8-*d*$_{10}$**	**9**	**8:7**
1	$Pd(PPh_3)_4$	-	10.8	17.9	3.2	1.7	39.9	0.5
2	$Pd(OAc)_2$	Xantphos	10.4	15.1	4.3	1.9	39.6	0.4
3	$Pd(OAc)_2$	Dppf	7.9	0.2	4.0	2.4	43.3	0.6
4	$Pd(OAc)_2$	Dippf	10.4	0.0	3.0	1.2	46.1	0.4

Table S5 *–*

GC-FID followed by GCMS

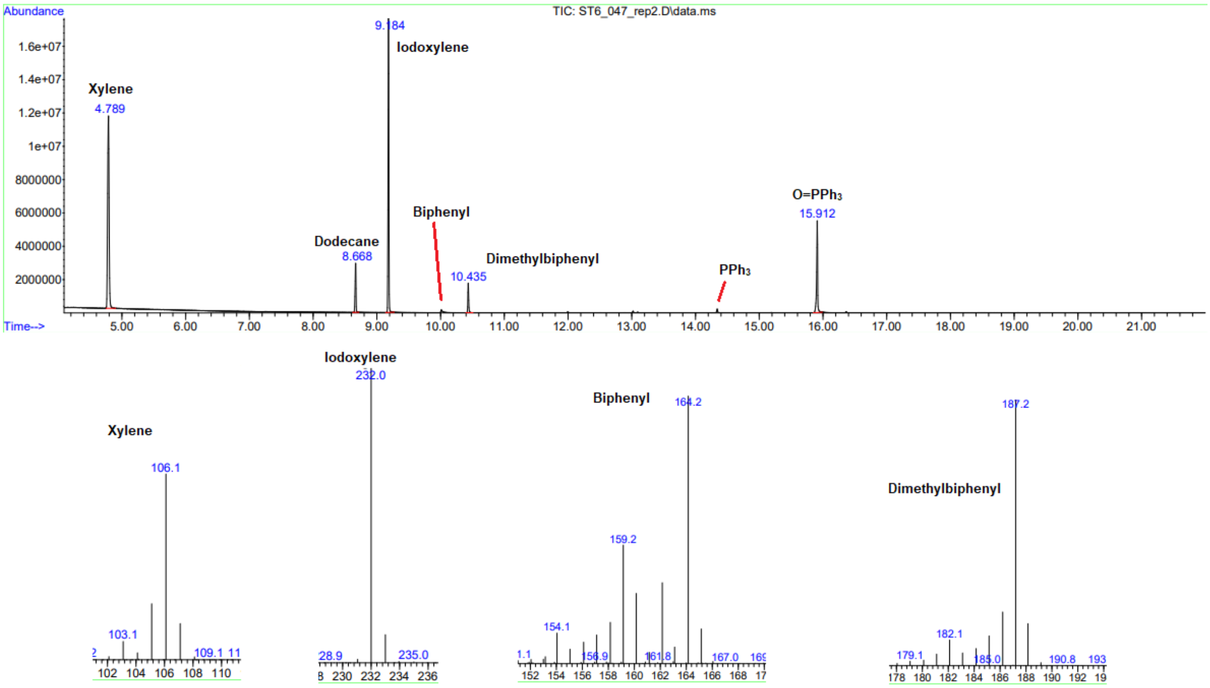

Figure S3 *– GC-MS trace for reaction of iodoxylene, $Pd(PPh_3)_4$ and Cs_2CO_3 in C_6D_6, accompanied by mass spectra for compounds **7**, **8** and **9**.*

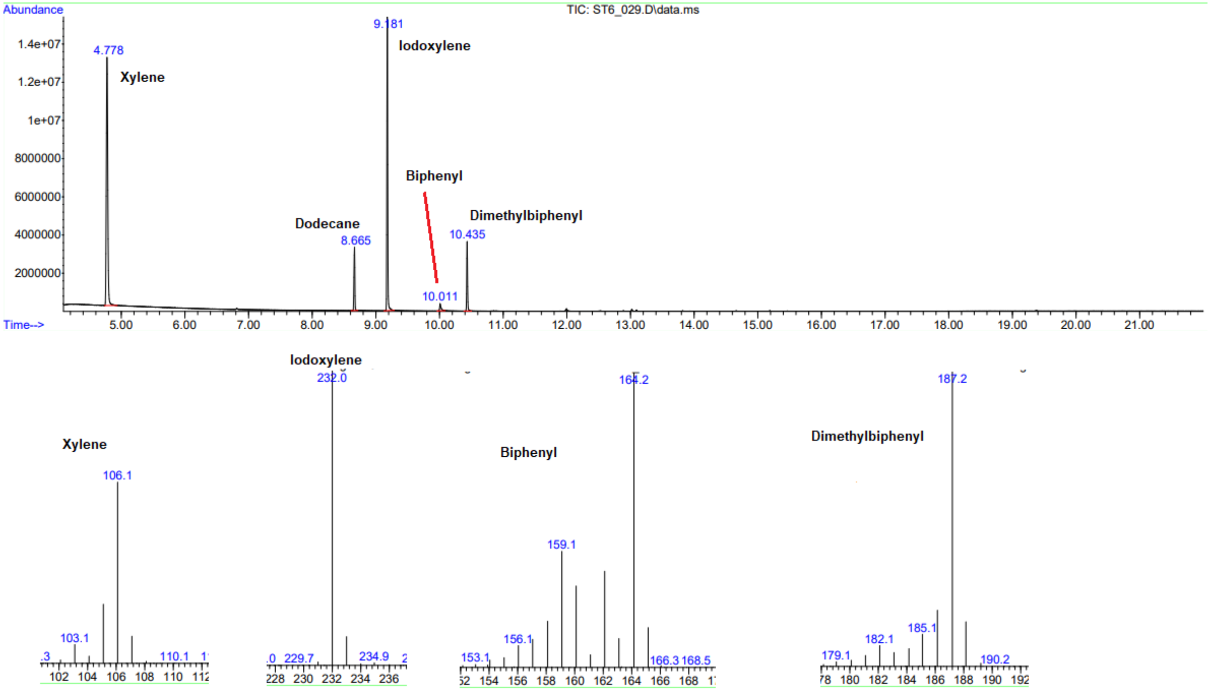

Figure S4 *– GC-MS trace for reaction of iodoxylene, Pd(OAc)*$_2$*, Xantphos and Cs*$_2$*CO*$_3$ *in C*$_6$*D*$_6$*, accompanied by mass spectra for compounds **7**, **8** and **9**.*

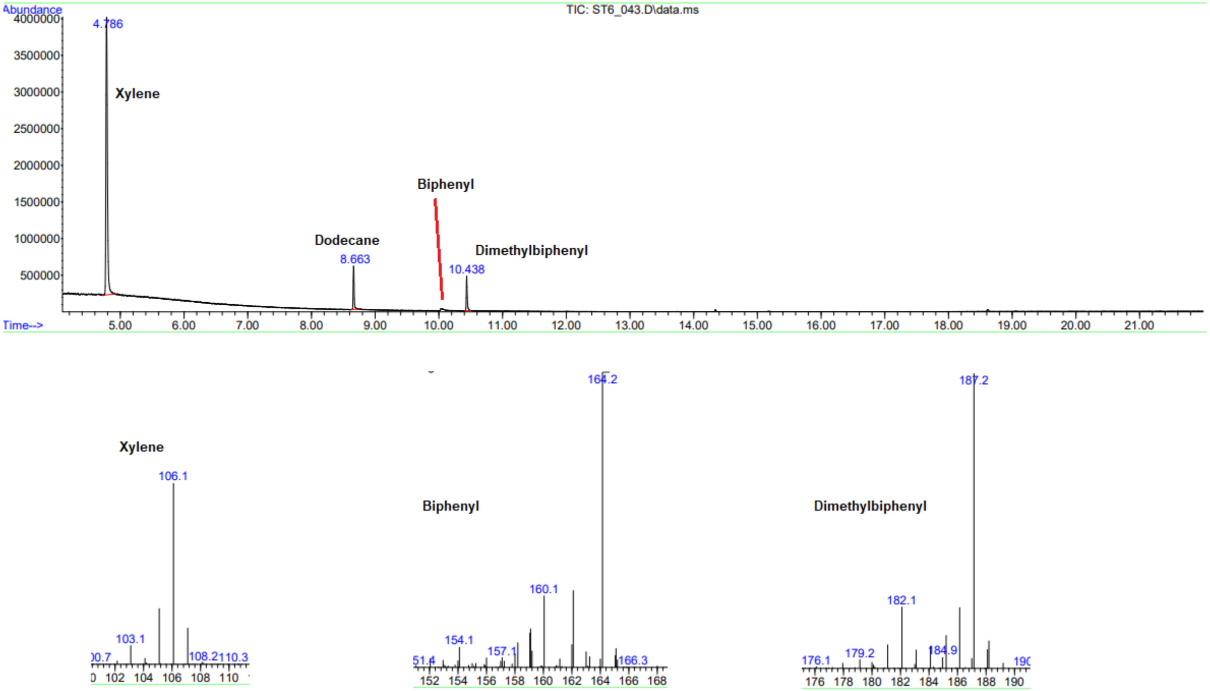

Figure S5 *– GC-MS trace for reaction of iodoxylene, Pd(OAc)*$_2$*, dppf and Cs*$_2$*CO*$_3$ *in C*$_6$*D*$_6$*, accompanied by mass spectra for compounds **7**, **8** and **9**.*

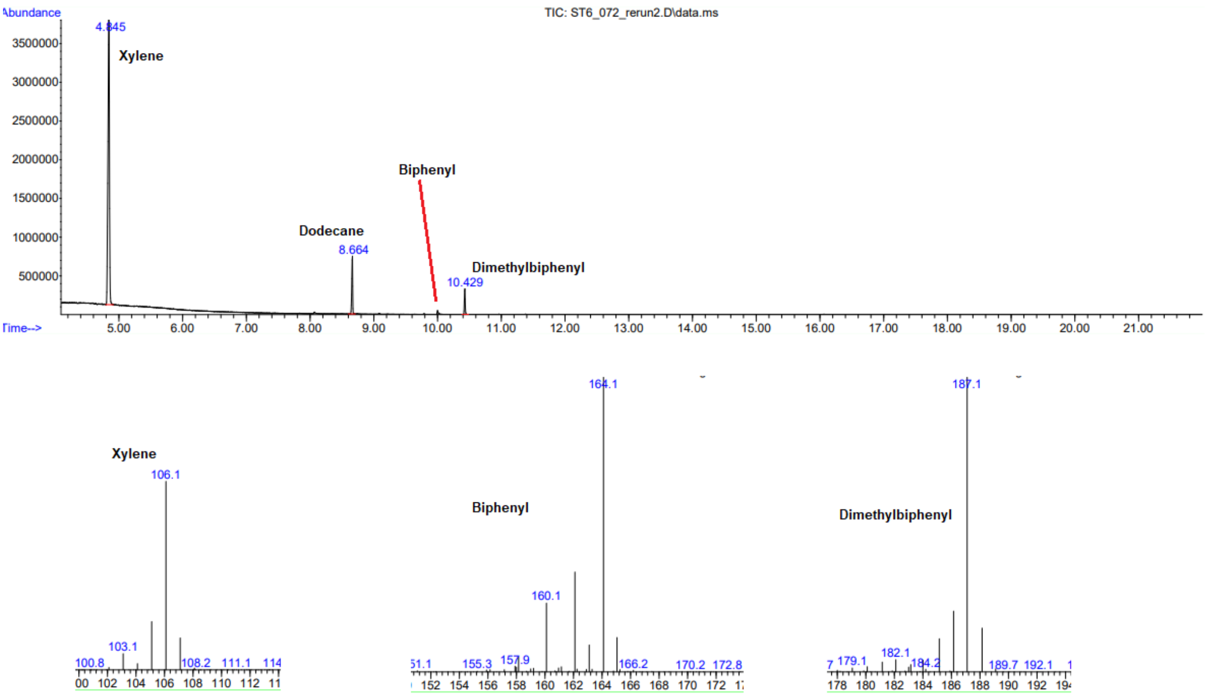

***Figure S6** – GC-MS trace for reaction of iodoxylene, $Pd(OAc)_2$, dippf and Cs_2CO_3 in C_6D_6, accompanied by mass spectra for compounds **7**, **8** and **9**.*

8.0 Synthesis of oxidative addition products $L_nPd(xylyl)(I)$

Synthesis of (dppf)Pd(xylyl)(I) 14a

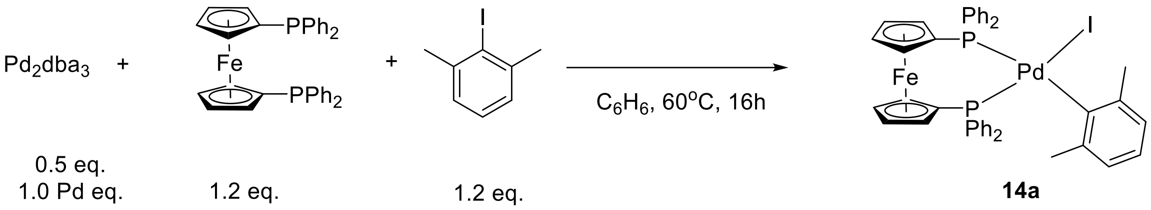

An oven-dried 25 ml pressure flask was charged in a glovebox with Pd_2dba_3 (228.9 mg, 0.25 mmol, 1.0 Pd eq.), dppf (332.63 mg, 0.6 mmol, 1.2 eq.), 2,6-dimethyliodobenzene (87 μL, 0.6 mmol, 1.2 eq.), benzene (8 mL) and a stirbar. The flask was sealed, removed from the glovebox and the purple solution was stirred at 60°C for 16 h yielding a brown solution. The solution was filtered through a short pad of celite, then concentrated to about 10% of the volume. The concentrate was then layered with pentane and allowed to recrystallize in a freezer. The crystals were then washed with further pentane and dried under reduced pressure, yielding (dppf)pd(xylyl)(I) **14a** as light orange crystals (304.4 mg, 0.333 mmol, 66.6 % wrt Pd). **^{1}H NMR (400 MHz, C_6D_6)** δ 8.37 (br, 4H), 7.48 – 7.27 (m, 4H), 7.26 – 7.20 (m, 4H), 7.15 – 7.10 (m, 2H), 6.91 – 6.86 (m, 2H), 6.77 – 6.72 (m, 4H), 6.67 (t, *J* = 7.1 Hz, 1H), 6.60 (br, 2H), 4.66 (br, 2H), 4.07 (br, 2H), 3.63 – 3.59 (m, 4H), 2.82 (s, 6H). **^{31}P NMR (162 MHz, C_6D_6)** δ 24.48 (d, *J* = 37.9 Hz), 6.68 (d, *J* = 37.8 Hz). The data match those for a sample reported previously by our group, for which a crystal structure was obtained.[1]

Synthesis of (*rac*-BINAP)Pd(xylyl)(I) 14b

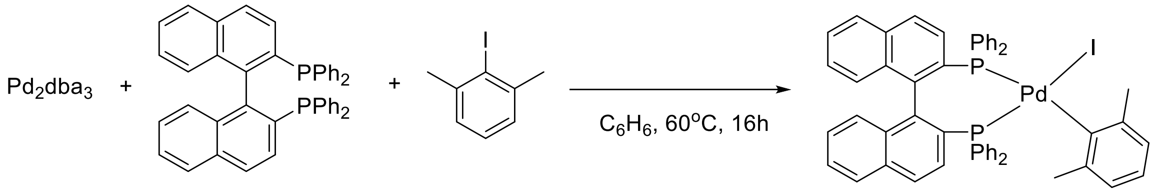

0.5 eq.
1.0 Pd eq. 1.2 eq. 1.2 eq. **14b**

An oven-dried 25 ml pressure flask was charged in a glovebox with Pd_2dba_3 (228.9 mg, 0.25 mmol, 1.0 Pd eq.), *rac*-BINAP (373.6 mg, 0.6 mmol, 1.2 eq.), 2,6-dimethyliodobenzene (87 μL, 0.6 mmol, 1.2 eq.), benzene (8 mL) and a stirbar. The flask was sealed, removed from the glovebox and the purple solution was stirred at 60°C for 16 h yielding an orange/brown solution and yellow solid. The flask was taken into a glovebox, and the contents transferred to a 100 mL round-bottom flask. Volatiles were removed under reduced pressure, yielding a dark orange solid. The residues were dissolved in toluene (100 mL), filtered to remove Pd black, and then toluene removed under reduced pressure. The orange solid was then triturated with THF until the washings were very pale yellow, after which solvent was removed under reduced pressure yielding (*rac*-BINAP)Pd(xylyl)(I) **14b** as a yellow powder (213.1 mg, 0.222 mmol, 44.3 % wrt Pd). **^{1}H NMR (400 MHz, THF-d$_8$)** δ 8.19 (dd, *J* = 10.6, 8.8 Hz, 1H), 7.83 (dd, *J* = 8.8, 1.5 Hz, 3H), 7.63 (d, *J* = 8.1 Hz, 2H), 7.58 (d, *J* = 8.1 Hz, 2H), 7.54 – 7.46 (m, 4H), 7.39 – 7.29 (m, 3H), 7.24 – 7.03 (m, 6H), 7.01 – 6.91 (m, 2H), 6.86 (ddd, *J* = 9.6, 8.4, 1.1 Hz, 2H), 6.78 – 6.61 (m, 7H), 6.56 (m, 1H), 6.48 (m, *J* = 8.5 Hz, 2H), 2.85 (s, 3H), 2.57 (s, 3H) **^{31}P NMR (162 MHz, THF-d$_8$)** δ 18.28 (d, *J* = 42.8 Hz), 7.71 (d, *J* = 42.8 Hz). **HRMS-ESI m/z**: 833.1709 $[M-I]^+$ (Calc for $C_{52}H_{41}P_2{}^{106}Pd$ 833.1713). Due to a complicated ^{1}H NMR spectrum, single crystals were grown from a solution of THF layered with hexane and analysed by X-ray diffraction, which was found to incorporate an equivalent of THF into the crystal. (For X-ray data, see later in S.I.)

Synthesis of *trans*-$(PPh_3)_2$Pd(xylyl)(I) 14c

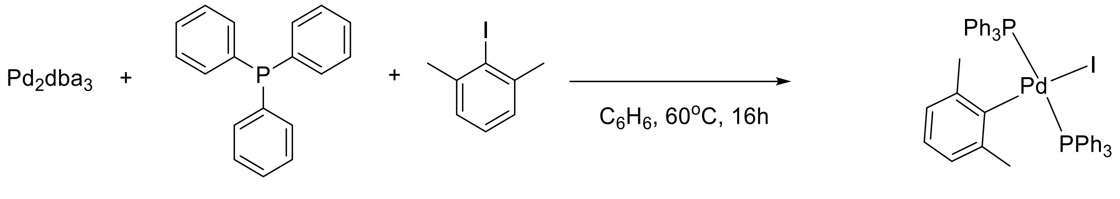

0.5 eq.
1.0 Pd eq. 2.4 eq. 1.2 eq. **14c**

An oven-dried 25 ml pressure flask was charged in a glovebox with Pd_2dba_3 (457.9 mg, 0.5 mmol, 1.0 Pd eq.), PPh_3 (629.5 mg, 2.4 mmol, 2.4 eq.), 2,6-dimethyliodobenzene (173 μL, 1.2 mmol, 1.2 eq.), benzene (10 mL) and a stirbar. The flask was sealed, removed from the glovebox and the purple solution was stirred at 60°C for 16 h yielding an orange solution with green insolubles. The mother liquor was removed and the insolubles dissolved in dry toluene (100 mL), then filtered to removed Pd black. The solvent was removed under reduced pressure, and then the solids washed with dry acetone until the washings ran clear. The product was recrystallized from DCM/acetone, yielding **14c** as white crystals (624.5 mg, 0.724 mmol, 72.4 % wrt Pd). **^{1}H NMR (400 MHz, C_6D_6)** δ 7.76 (br, 12H),

7.05 – 6.93 (m, 18H), 6.53 (t, *J* = 7.3 Hz, 1H), 6.20 (d, *J* = 7.3 Hz, 2H), 2.35 (s, 6H). **^{31}P NMR (162 MHz, C_6D_6)** δ 22.43. **HRMS-ESI** *m/z*: 735.1551 $[M-I]^+$ (Calc. for $C_{44}H_{39}P_2{}^{106}Pd$, 735.1556).

Synthesis of *trans*-$(PCy_3)_2$Pd(xylyl)(I) 14d

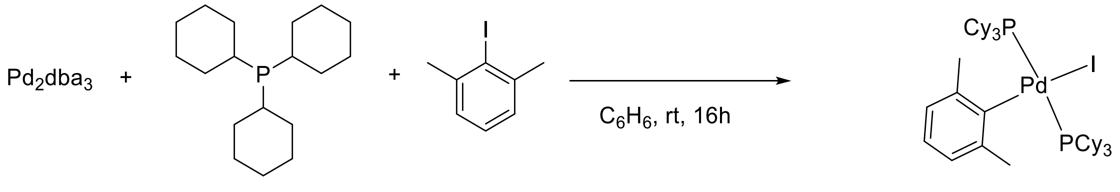

0.5 eq.
1.0 Pd eq. 2.4 eq. 1.2 eq. **14d**

An oven-dried 25 ml pressure flask was charged in a glovebox with Pd_2dba_3 (228.9 mg, 0.25 mmol, 1.0 Pd eq.), PCy_3 (336.5 mg, 1.2 mmol, 2.4 eq.), 2,6-dimethyliodobenzene (87 μL, 0.6 mmol, 1.2 eq.), benzene (5 mL) and a stirbar. The flask was sealed, removed from the glovebox and the purple solution was stirred at room temperature for 16 h yielding a dark orange solution with pale green insolubles. Solvent was removed under reduced pressure, then the solids were dissolved in DCM (100 mL) and filtered to removed Pd black. Solvent was removed under reduced pressure, then the solids washed with dry acetone until the washings were colourless. The solids were then recrystallized from DCM/acetone, yielding **14d** as white crystals (247.2 mg, 0.275 mmol, 55.0% wrt Pd). **^{1}H NMR (400 MHz, $CDCl_3$)** δ 6.83 (t, *J* = 7.3 Hz, 1H), 6.69 (d, *J* = 7.2 Hz, 2H), 2.67 (s, 6H), 2.38 – 2.21 (m, 6H), 2.09 – 1.93 (m, 12H), 1.73 – 1.55 (m, 18H), 1.44 – 1.28 (m, 12H), 1.20 – 1.00 (m, 18H). **^{31}P NMR (162 MHz, $CDCl_3$)** δ 23.79. **HRMS-ESI** *m/z*: 771.4364 $[M-I]^+$ (Calc. for $C_{44}H_{75}P_2{}^{106}Pd$, 771.4373).

Synthesis of *trans*-$(P^iPr_3)_2$Pd(xylyl)(I) 14e

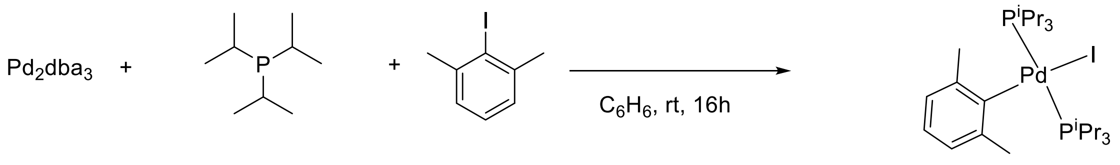

0.5 eq.
1.0 Pd eq. 2.4 eq. 1.2 eq. **14e**

In the glovebox, a 25 mL pressure flask was charged with Pd_2dba_3 (228.9 mg, 0.25 mmol, 1.0 Pd eq.), P^iPr_3 (229 μL, 1.2 mmol, 2.4 eq.), 2,6-dimethyliodobenzene (87 μL, 0.6 mmol, 1.2 eq.), and stirbar and dry benzene (5 mL). The vial was capped, removed from the glovebox and the purple solution was stirred at room temperature for 16 h, yielding a red solution. The mixture was filtered to removed palladium black and evaporated to dryness. The crude was the purified by sequential recrystallization from DCM/acetone in a freezer to remove dimers, and yielded **14e** as pale yellow crystals (98.2 mg, 0.149 mmol, 29.8 %). **^{1}H NMR (400 MHz, $CDCl_3$)** δ 6.86 – 6.80 (t, *J* = 8.0 Hz, 1H), 6.71 (d, *J* = 8.0 Hz, 2H), 2.71 (s, 6H), 2.70 – 2.60 (m, 6H), 1.21 (dd, *J* = 14.0, 7.1 Hz, 36H). **^{31}P NMR (162 MHz, $CDCl_3$)** δ 29.35. **HRMS-ESI** *m/z*: 531.2500 $[M-I]^+$ (Calc. for $C_{26}H_{51}P_2{}^{106}Pd$, 531.2495).

9.0 Pd(Xantphos)$_2$

Attempted synthesis of (Xantphos)Pd(xylyl)(I) – Isolation of Pd(Xantphos)$_2$

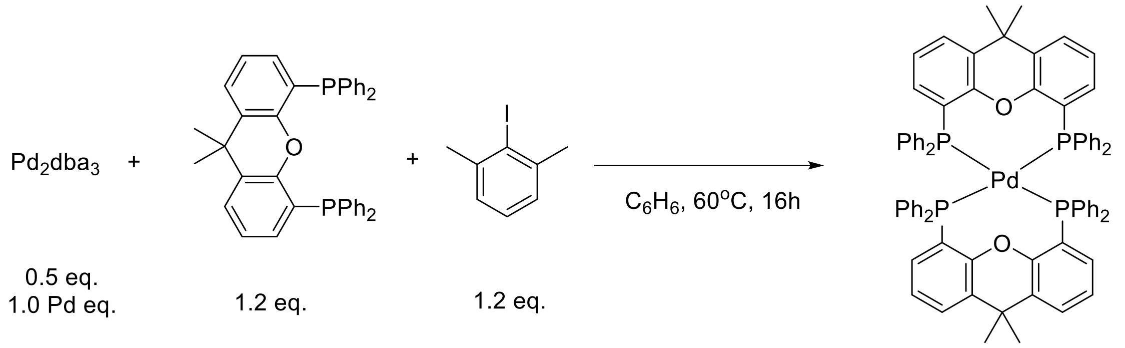

An oven-dried 25 ml pressure flask was charged in a glovebox with Pd_2dba_3 (228.9 mg, 0.25 mmol, 1.0 Pd eq.), Xantphos (347.3 mg, 0.6 mmol, 1.2 eq.), 2,6-dimethyliodobenzene (86.6 μL, 0.6 mmol, 1.2 eq.), benzene (6 mL) and a stirbar. The flask was sealed, removed from the glovebox and the purple solution was stirred at 60°C for 16 h yielding a brown solution and yellow solid. The flask was taken into a glovebox, and the mother liquor removed leaving a yellow-green solid. After washing with various solvents (benzene, toluene, THF, DCM, chloroform and acetone) in which the solid was insoluble, ESI-MS confirmed the identity of the solid as Pd(Xantphos)$_2$.[2] The green tinge is due to traces of Pd black. **MS-ESI** *m/z*: 1262.7

Independent synthesis of Pd(Xantphos)$_2$

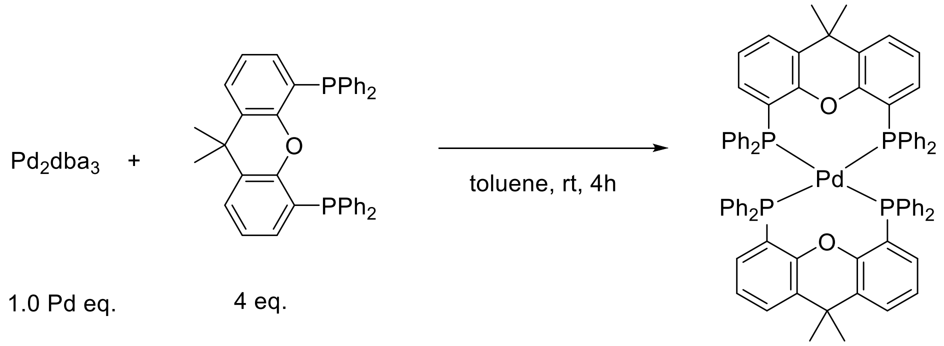

Pd(Xantphos)$_2$ was independently synthesised following a modified procedure reported by Buchwald et al.[2] An oven-dried 500 mL 3-neck flask was charged with Pd_2dba_3 (229 mg, 0.25 mmol, 1 Pd eq.), Xantphos (579 mg, 1 mmol, 4 eq.) and a stirbar, then evacuated and refilled with argon three times. Dry toluene (300 mL) was added and the dark purple solution was stirred for 4 h, yielding a bright yellow solution. The solution was allowed to stand overnight after which Pd black had settled to the bottom of the flask. The solution was then transferred to an oven-dried 500 mL round-bottom flask *via* cannula filter, and solvent was removed under reduced pressure. Excess dba was removed by washing with dry toluene (50 mL x 3, solid separated from supernatant by centrifuge after each wash), yielding Pd(Xantphos)$_2$ as a bright yellow solid (492 mg, 0.39 mmol, 78 %), identified by HRMS. The solid is too insoluble to analyse by NMR once isolated, but NMR was used to confirm that no other species were present. **HRMS-ESI** *m/z*: 1262.2879 (Calc. for $C_{76}H_{64}O_2P_4{}^{106}Pd$, 1262.2886).

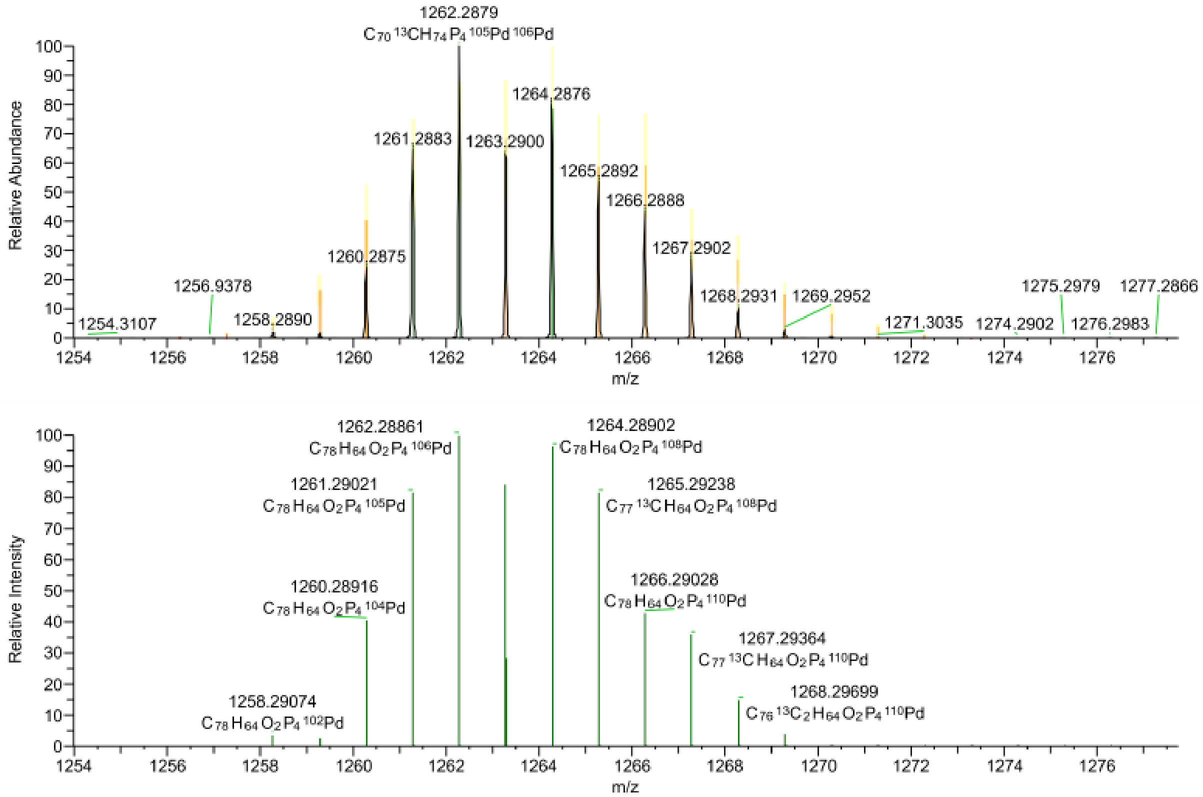

Figure S7 – top – observed mass spectrum; below – calculated values.

Using $Pd(Xantphos)_2$ as a precatalyst in assay reaction

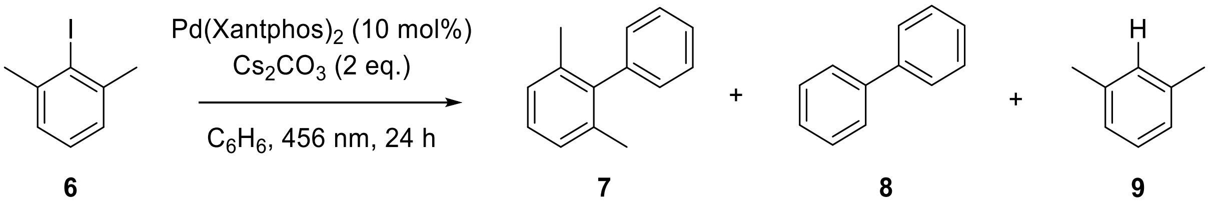

This reaction was carried out according to General Procedure A using $Pd(Xantphos)_2$ (44.2 mg, 0.035 mmol, 0.1 eq.). Under ground-state conditions Buchwald found $Pd(Xantphos)_2$ to be an unreactive species that removed Pd from the catalytic cycle.[2] Under these photoexcited conditions $Pd(Xantphos)_2$ yielded a very similar outcome to results obtained from a combination of $Pd(OAc)_2$/Xantphos, with slightly higher yields.

Entry	Dodecane (mg)	% Yield				Ratio 8:7
		6	7	8	9	
1	10.3	9.3	5.6	27.1	57.2	4.8

Table S6 –

Formation of $Pd(Xantphos)_2$ *in situ* from $Pd(OAc)_2$ + Xantphos

To observe the formation of $Pd(Xantphos)_2$ in solution, an NMR tube was charged in a glovebox with $Pd(OAc)_2$ (2.2 mg, 0.01 mmol, 1 eq.), Xantphos (23.1 mg, 0.04 mmol, 4 eq.) and C_6D_6 (0.7 mL).

^{31}P NMR:

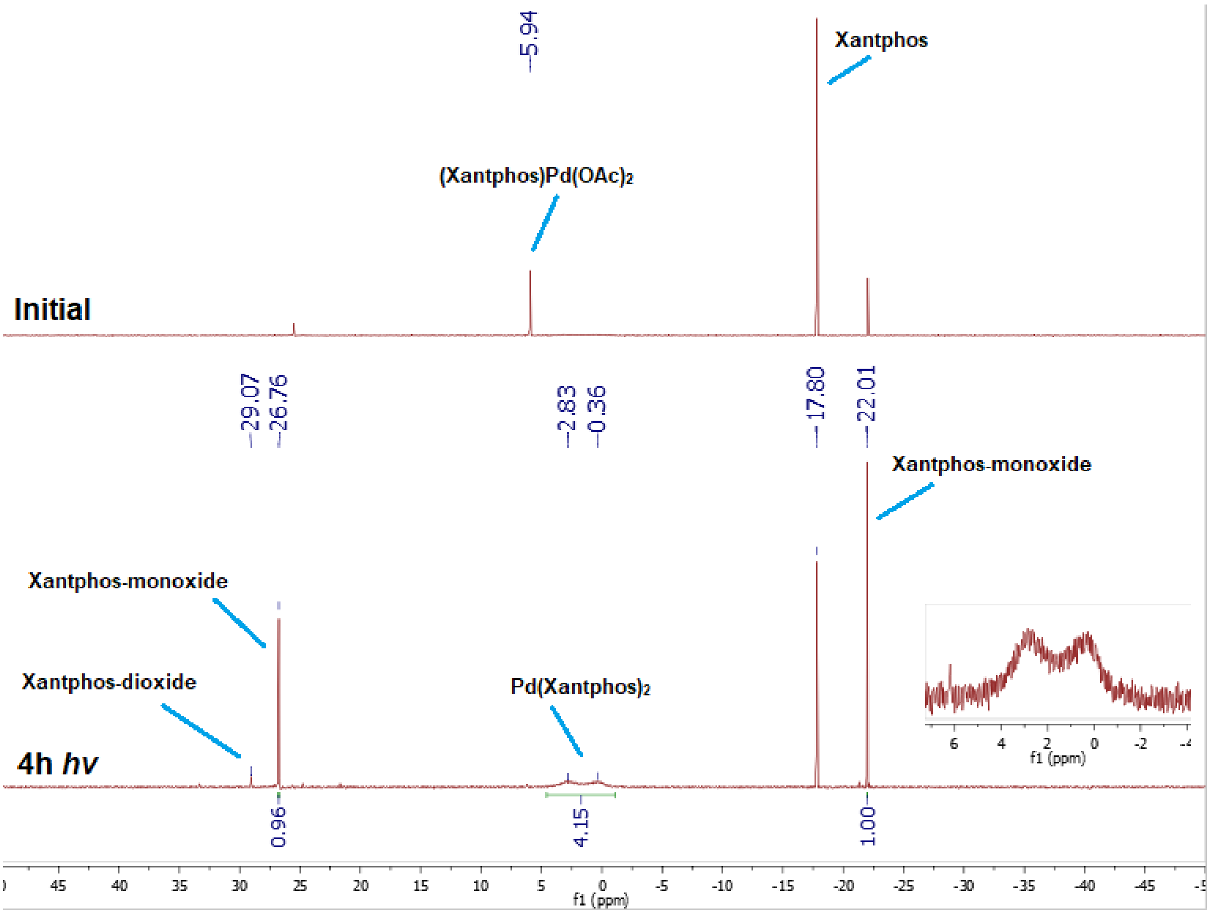

Figure S8 -

The solution was then monitored by ^{31}P NMR. In addition to Xantphos (δ –17.80 ppm), a singlet was initially observed to form at δ 5.94 ppm, which we attribute to *trans*-$(Xantphos)Pd(OAc)_2$, along with small quantities of Xantphos-monoxide (δ –22.01 and 26.76 ppm) and $Pd(Xantphos)_2$ (broad double peak, centred at δ 1.60 ppm). The reaction was then irradiated at 456 nm. After 4 h the $(Xantphos)Pd(OAc)_2$ peak had completely disappeared, replaced by an increase in $Pd(Xantphos)_2$ and Xantphos-monoxide in a ratio of *ca.* 1:1, along with small quantities of Xantphos-dioxide (δ 29.07 ppm). The complicated signal observed for $Pd(Xantphos)_2$ is similar to that observed by Buchwald et al. for related but more soluble compound $Pd(4,7\text{-di-tert-butylXantphos})_2$, which was proposed to fluctuate between two states due to steric crowding around the Pd centre.[2]

10.0 Reactions of oxidative addition products $L_nPd(xylyl)(I)$

Reactions of (dppf)Pd(xylyl)(I) 14a

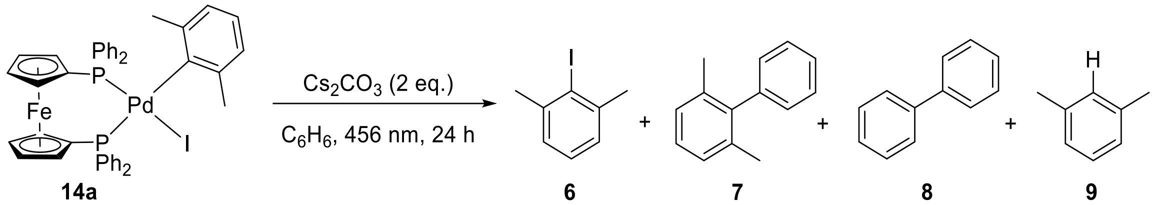

These reactions were carried out according to General Procedure B, using (dppf)Pd(xylyl)(I) **14a** (33.9 mg, 0.035 mmol, 1 eq.).

Entry	Conditions changed	Dodecane (mg)	% Yield				Ratio 8:7
			6	7	8	9	
1	-	7.9	0.0	15.1	72.0	83.0	4.8
2	No Cs_2CO_3	8.5	0.0	15.9	72.3	80.9	4.8

Table S7 –

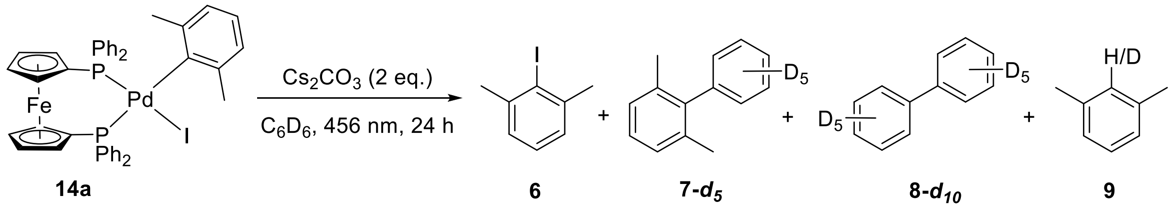

This reaction was carried out according to General Procedure B, using (dppf)Pd(xylyl)(I) **14a** (33.9 mg, 0.035 mmol, 1 eq.) and C_6D_6 (2.5 mL).

Entry	Dodecane (mg)	% Yield				Ratio 8:7
		1	2-d_5	3-d_{10}	4	
1	8.4	0.0	52.2	30.5	42.7	0.6

Table S8 –

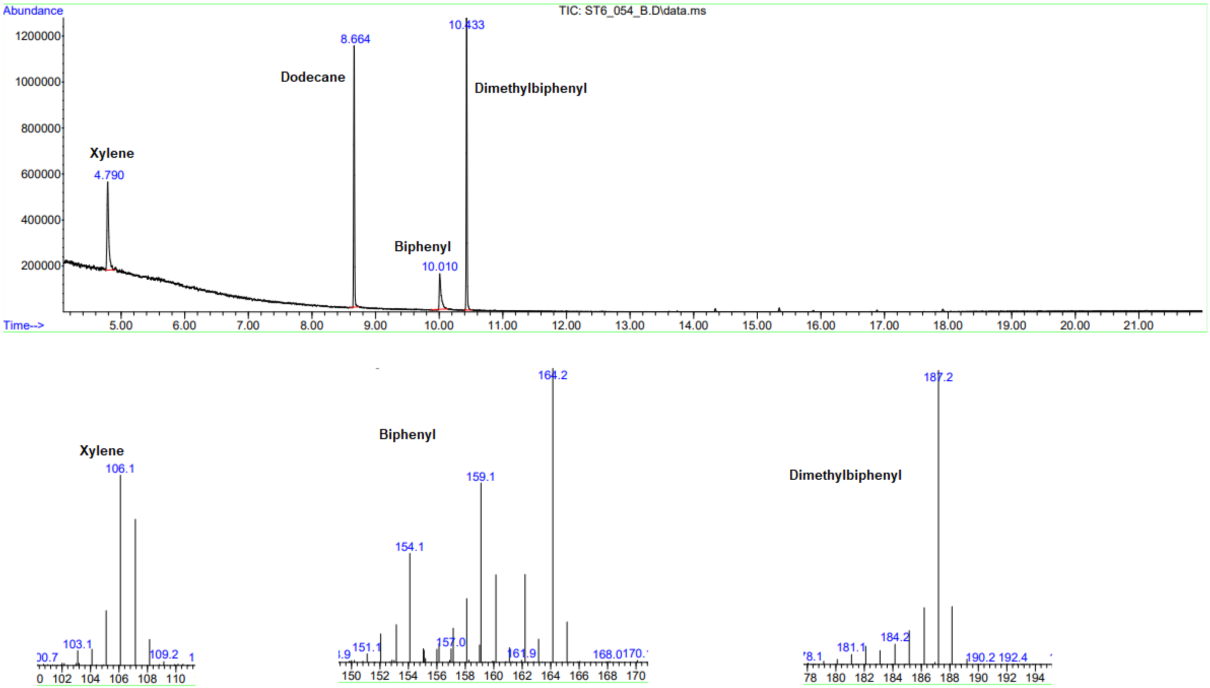

Figure S9 *- GC-MS trace for reaction of (dppf)Pd(xylyl)(I) and Cs_2CO_3 in C_6D_6, accompanied by mass spectra for deuterated compounds* ***6****,* ***7*** *and* ***8.***

Reactions of (*rac*-BINAP)Pd(xylyl)(I) 14b

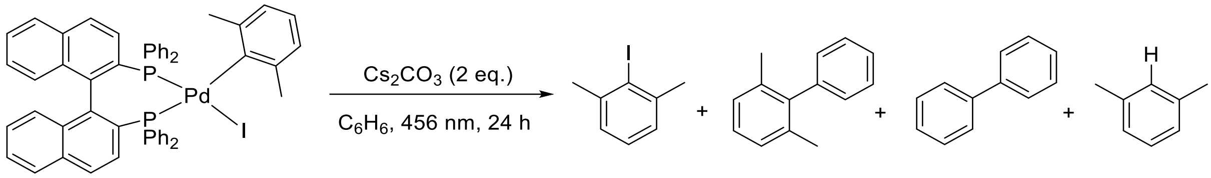

These reactions were carried out according to General Procedure B, using (BINAP)Pd(xylyl)(I) **14b** (33.6 mg, 0.035 mmol, 1 eq.).

Entry	Conditions changed	Dodecane (mg)	% Yield				Ratio 8:7
			6	7	8	9	
1	-	11.0	2.0	10.8	42.9	64.1	4.0
2	No base	10.8	4.8	9.0	42.1	60.8	4.7

Table S9 *–*

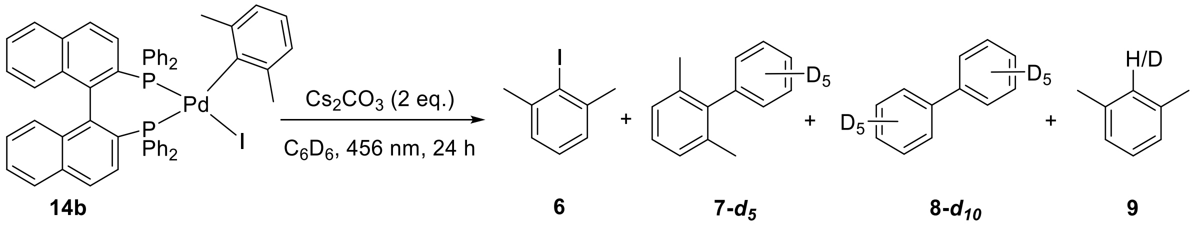

This reaction was carried out according to General Procedure B, using (BINAP)Pd(xylyl)(I) **14b** (33.6 mg, 0.035 mmol, 1 eq.) and C_6D_6 (2.5 mL).

Entry	Dodecane (mg)	% Yield				Ratio 8:7
		6	7-d_5	8-d_{10}	9	
1	10.1	4.2	26.4	10.9	41.0	0.4

Table S10 –

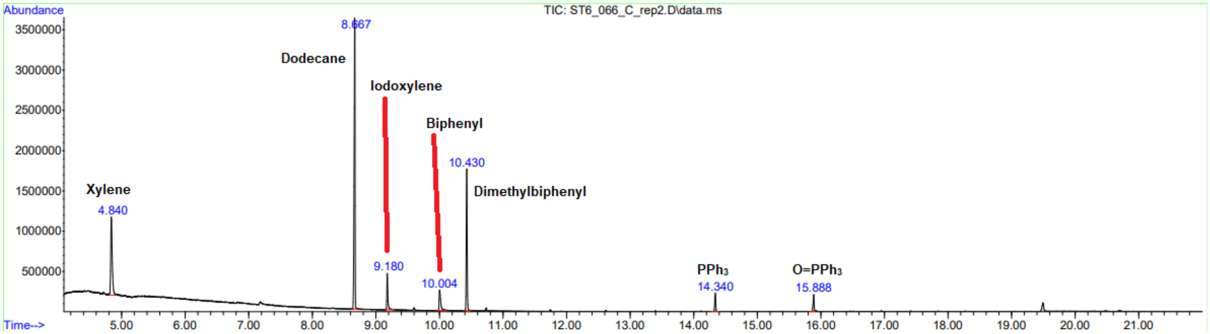

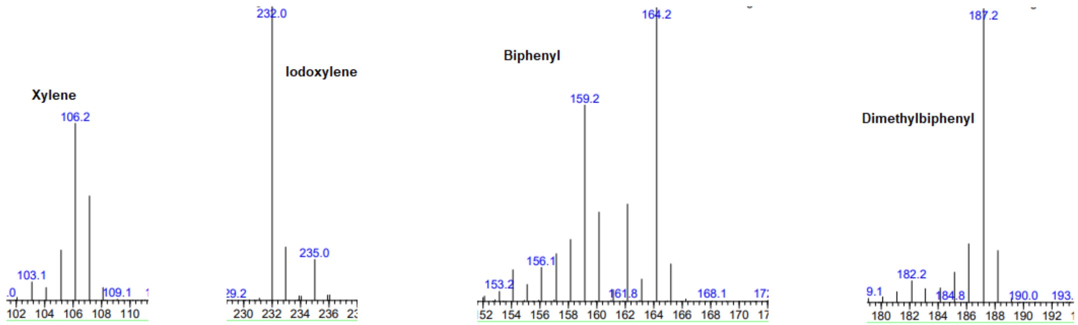

Figure S10 - *GC-MS trace for reaction of (BINAP)Pd(xylyl)(I) and Cs_2CO_3 in C_6D_6, accompanied by mass spectra for deuterated compounds **7, 8** and **9**.*

Reactions of *trans*-(PPh_3)$_2$Pd(xylyl)(I) 14c

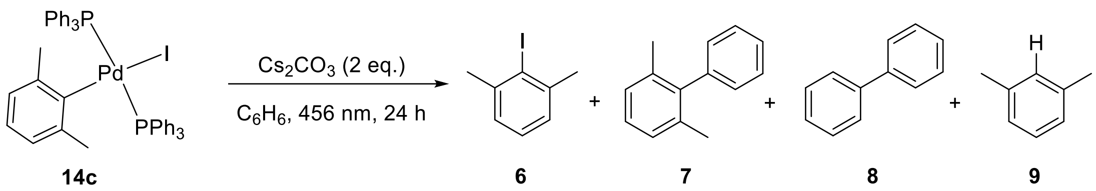

These reactions were carried out according to General Procedure B, using (PPh_3)$_2$Pd(xylyl)(I) **14c** (30.2 mg, 0.035 mmol, 1 eq.).

Entry	Dodecane (mg)	% Yield				Ratio 8:7
		6	7	8	9	
1	8.2	7.2	15.7	45.8	61.1	2.9

Table S11 –

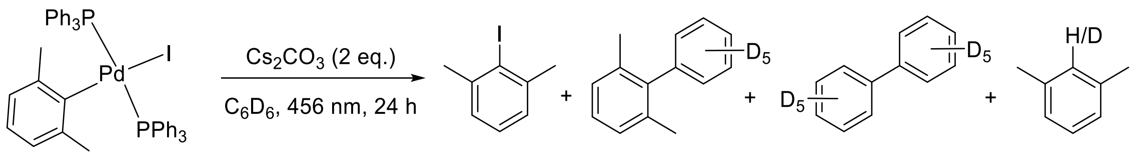

14c **6** **7-*d*$_5$** **8-*d*$_{10}$** **9**

This reaction was carried out according to General Procedure B, using $(PPh_3)_2$Pd(xylyl)(I) **14c** (30.2 mg, 0.035 mmol, 1 eq.) and C_6D_6 (2.5 mL).

Entry	Dodecane (mg)	% Yield				Ratio 3:2
		6	7-*d*$_5$	8-*d*$_{10}$	9	
1	8.4	0.0	52.2	30.5	42.7	0.6

Table S12 –

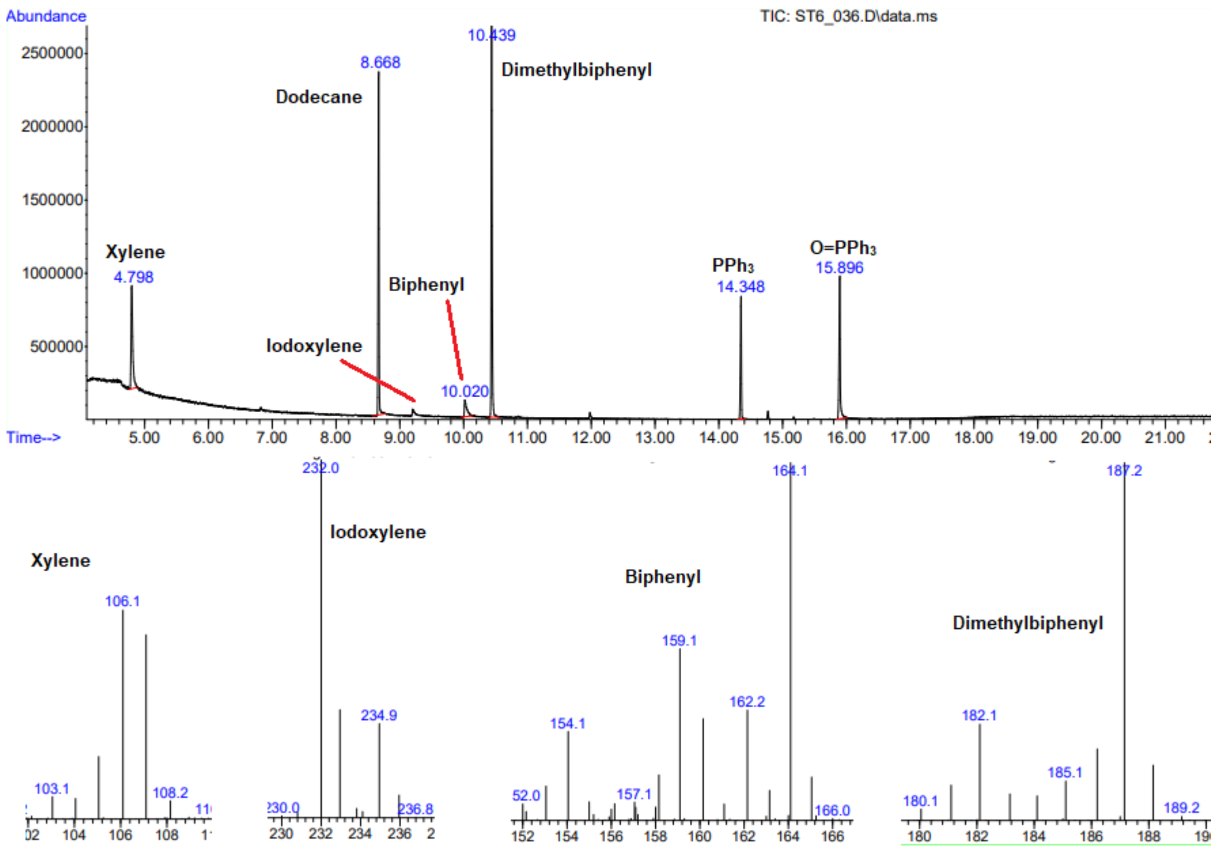

Figure S11 - *GC-MS trace for reaction of $(PPh_3)_2$Pd(xylyl)(I) and Cs_2CO_3 in C_6D_6, accompanied by mass spectra for deuterated compounds **7**, **8** and **9**.*

Reaction of *trans*-$(PCy_3)_2Pd(xylyl)(I)$ 14d

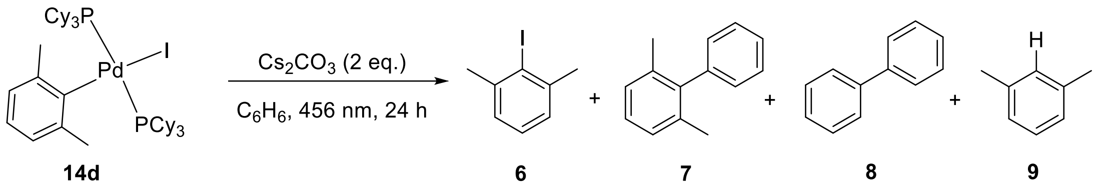

These reactions were carried out according to General Procedure B, using $(PCy_3)_2Pd(xylyl)(I)$ (31.4 mg, 0.035 mmol, 1 eq.).

Entry	Dodecane (mg)	% Yield				Ratio 8:7
		6	7	8	9	
1	7.5	26.2	2.3	9.0	54.1	3.9

Table S13 –

Reaction of *trans*-$(P^iPr_3)_2Pd(xylyl)(I)$ 14e

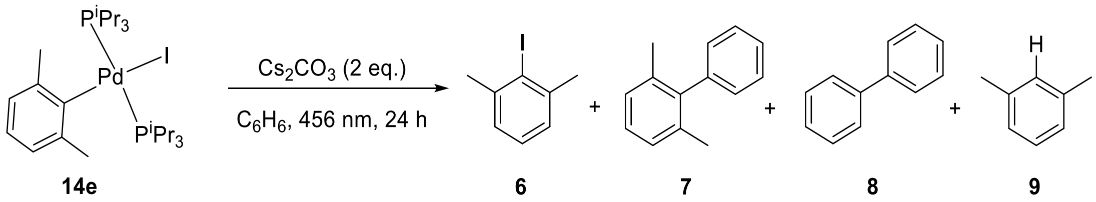

These reactions were carried out according to General Procedure B, using $(P^iPr_3)_2Pd(xylyl)(I)$ **14e** (23.1 mg, 0.035 mmol, 1 eq.).

Entry	Dodecane (mg)	% Yield				Ratio 8:7
		6	7	8	9	
1	7.8	23.6	3.6	12.2	74.3	3.4

Table S14 –

Thermal dark reactions of complexes 14a-e

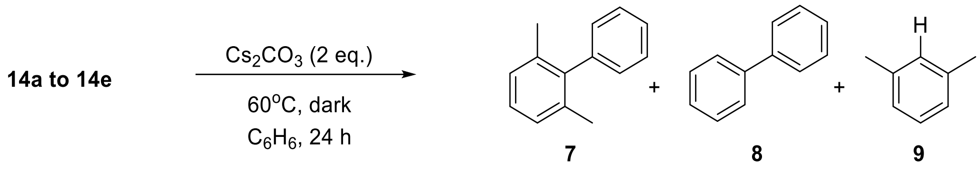

These reactions were carried out according to General Procedure B, using complexes **14a-e** (0.035 mmol, 1 eq.), except the reactions were carried out in the dark with heating at 60°C.

Entry	Complex	Dodecane (mg)	% Yield			Ratio 8:7
			7	8	9	
1	**14a**	5.2	1.4	3.0	2.4	2.2
2	**14b**	5.8	0.1	0.6	0.9	6.5
3	**14c**	5.0	3.8	2.5	7.5	0.7
4	**14d**	5.5	0.1	0.0	24.2	-
5	**14e**	5.4	0.0	0.0	11.2	-

Table S15 –

We see that for all complexes **14a-e**, yields of coupled products when reacted thermally in the dark (Table S15) are far lower than the analogous reactions carried out under 456 nm irradiation. Notably, for complexes **14d** and **14e**, which contain no phenyl groups on their ligands (and thus cannot intramolecularly form dimethylbiphenyl **7** or biphenyl **8**), products **7** and **8** are virtually absent. This experiment highlights the role of visible light in the homolysis of the Pd–Ar bond.

11.0 UV-Visible absorption spectra of oxidative addition complexes

In a glovebox, solutions of complexes **14a-e** were made up in benzene (2.5 mM). Then 200 μL of these solutions were diluted to 3 mL with benzene (0.167 mM) and analysed by UV-Visible spectroscopy in 1 cm quartz cuvettes.

For complexes (Dppf)Pd(xylyl)(I) **14a** and (BINAP)Pd(xylyl)(I) **14b**, significant overlap of their absorption spectra with the output of the Kessil lamp is observed (Figure S12). By contrast, complexes **14c-e** with monodentate ligands show much poorer spectral overlap, suggesting a shorter wavelength may be more appropriate to excite these complexes and promote homolysis of the Pd–Ar bond.

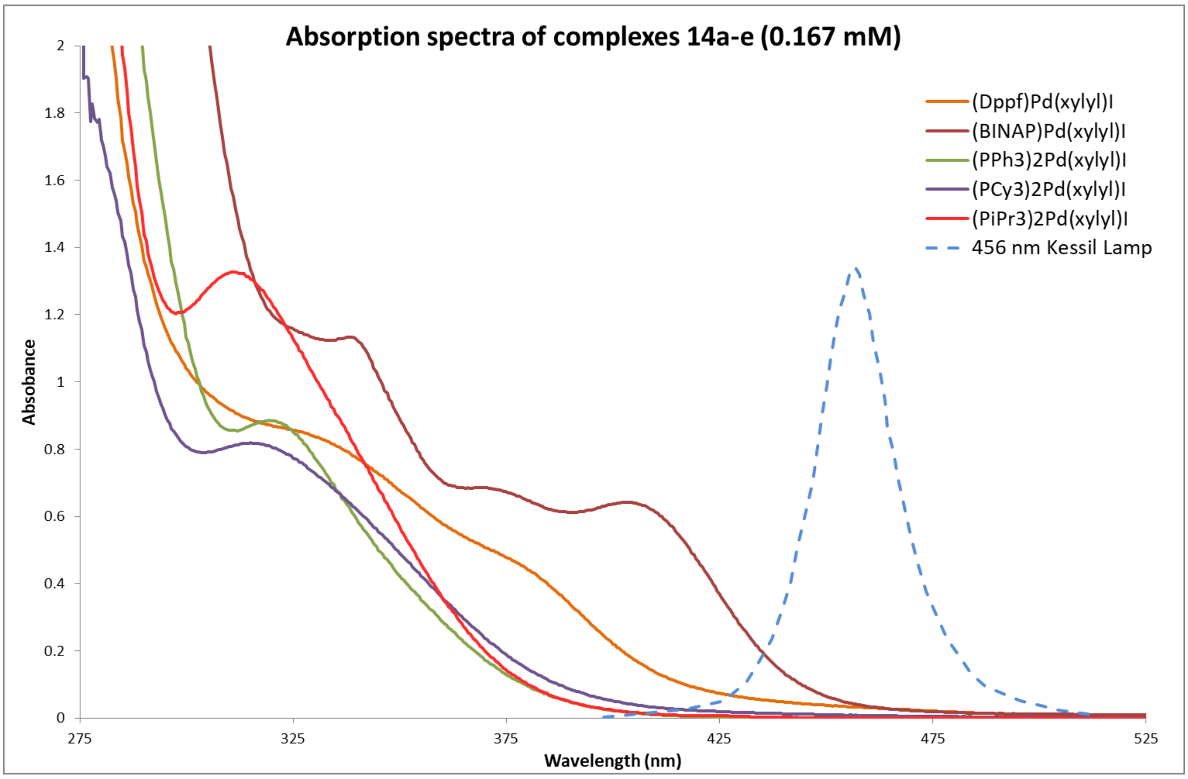

Figure S12 – Absorption spectra of complexes 14a-e in benzene (0.167 mM), overlaid with the emission of the 456 nm Kessil lamp used (spectrum acquired from the manufacturer's webpage).

12.0 Stern-Volmer quenching studies

Quenching using $Pd(PPh_3)_4$

To carry out Stern-Volmer quenching studies, initially samples of a constant concentration of well-defined palladium species $Pd(PPh_3)_4$ were made up with varying concentrations of 2-iodo-*m*-xylene within a glovebox, as shown in the below table. The solutions were transferred to air-tight cuvettes and analysed on a fluorometer (Excitation 380 nm, Slits 20 nm/20 nm, 400 nm long-pass filter). Emission spectra were taken in order of increasing 2-iodo-*m*-xylene concentration. The emission spectrum observed corresponds to that of $Pd(PPh_3)_3$ formed by loss of one PPh_3 ligand in solution.

Total volume (µL)	Vol 10 mM $Pd(PPh_3)_4$ stock (µL)	Conc. $Pd(PPh_3)_4$ (mM)	Vol 50 mM iodoxylene stock (µL)	Conc. Iodoxylene (mM)	Vol benzene (µL)
3000	200	2	0	0	2800
3000	200	2	40	0.67	2760
3000	200	2	60	1.0	2740
3000	200	2	80	1.3	2720
3000	200	2	120	2.0	2680

Table S16 –

An overlaid emission spectrum is provided below (Figure S13) and a Stern-Volmer plot (Figure S14) of [ArI] vs. I_0/I shows that the excited state of $Pd(PPh_3)_3$ is quenched by increasing concentrations of 2-iodo-*m*-xylene.

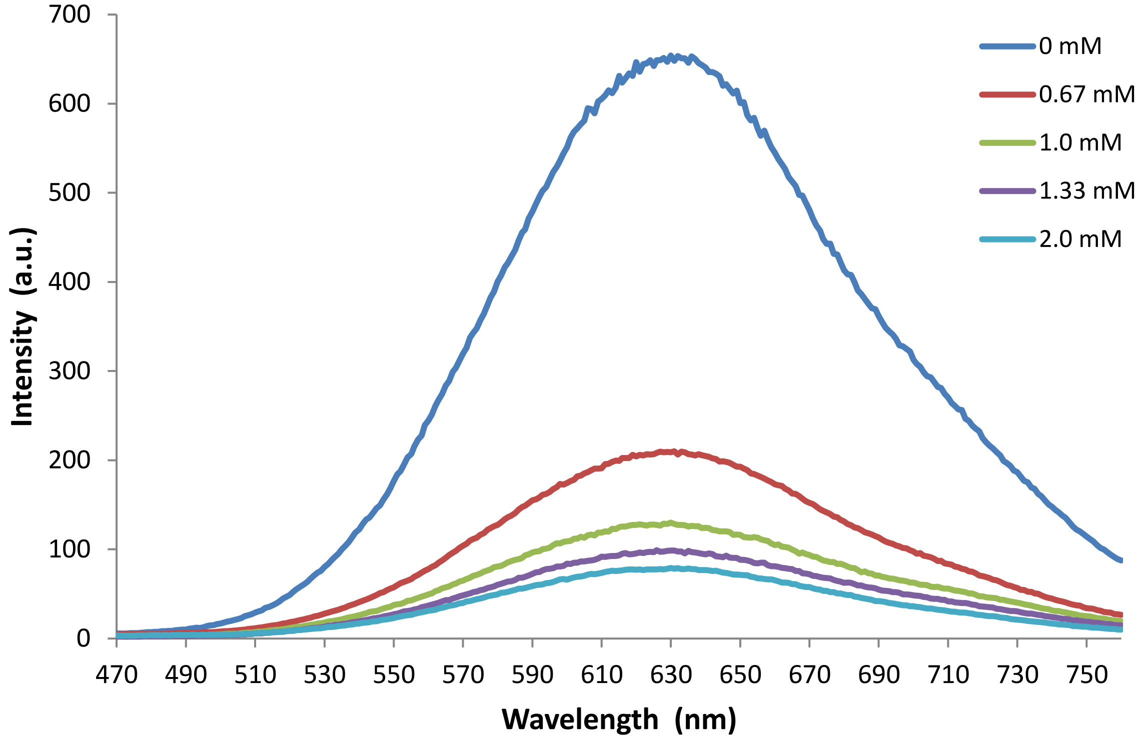

Figure S13 - Overlaid emission spectra of solutions of constant [Pd(PPh$_3$)$_4$] showing emission quenching with increasing [ArI].

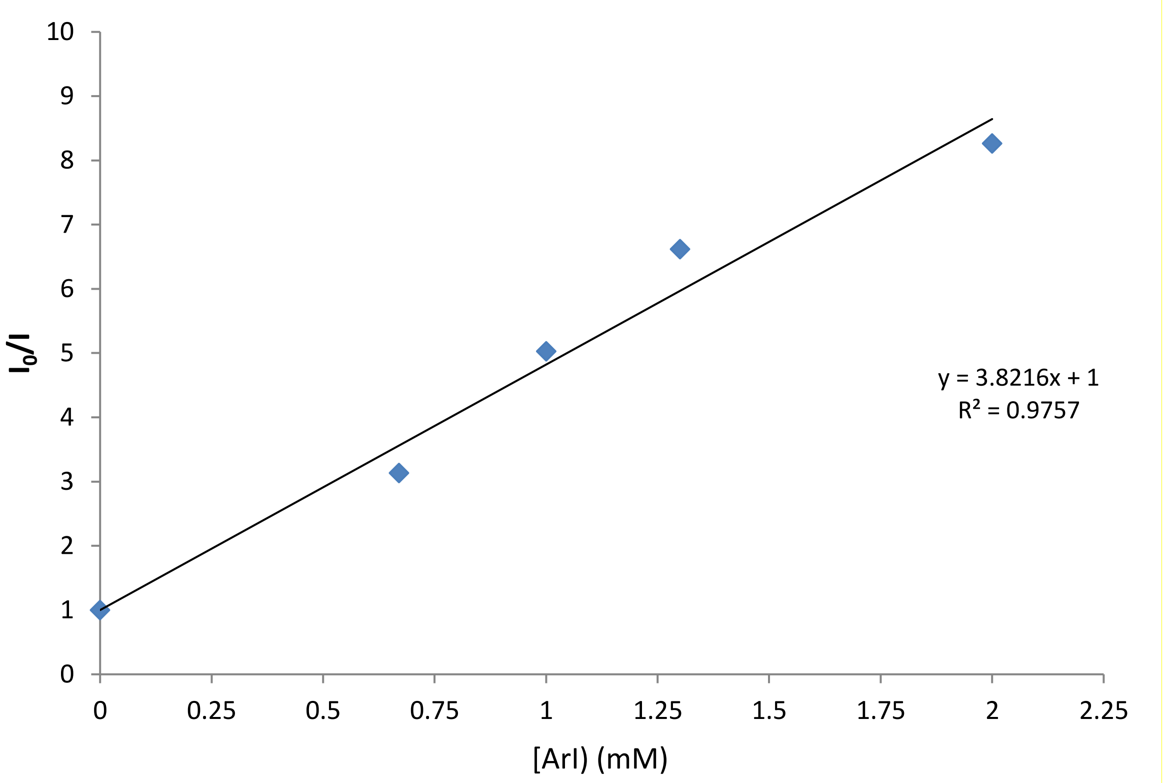

Figure S14 – *Stern-Volmer plot for the emission quenching of Pd(PPh$_3$)$_4$ by ArI*

13.0 Testing for a chain reaction

Formation of DTBB radical anion[3]

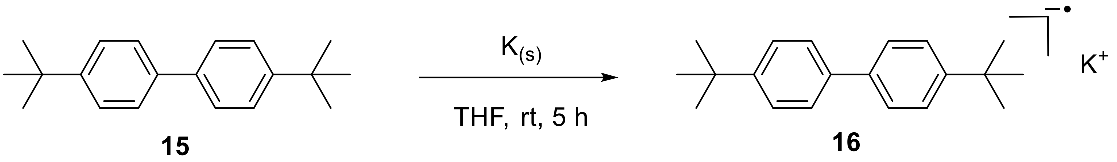

A 10 mL oven-dried microwave vial was cycled into a glovebox. To this was added 4,4'-ditertbutylbiphenyl **15** (79.9 mg, 0.3 mmol) and potassium metal (11.7 mg, 0.3 mmol, 1 eq.). The vial was capped and removed from the glovebox, after which dry THF (1 mL) was added by syringe under a flow of argon. With stirring, a deep blue/green solution was formed rapidly. After 5 h, stirring was stopped to allow unreacted potassium metal to settle, and the solution was titrated against an I_2/THF solution giving the concentration of radical anion **16** as 0.144 M (Batch 1). The reaction was repeated as above, with titration determining a concentration of **16** of 0.149 M (Batch 2).

Initiation with DTBB radical anion 16

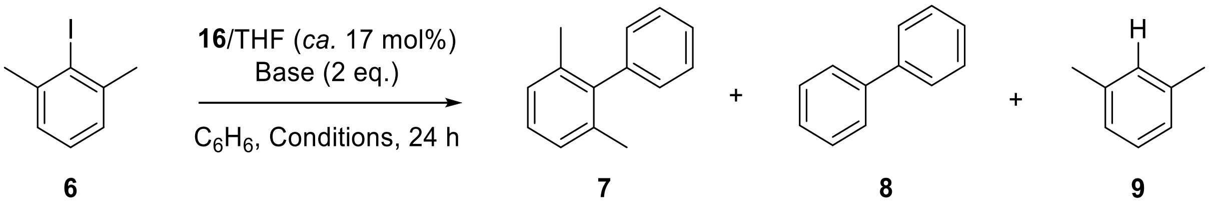

For each reaction a 10 mL oven-dried microwave vial was cycled into a glovebox. To this was added **1** (50.5 μL, 0.35 mmol, 1 eq.), a base [either KO^{t}Bu (78.6 mg, 0.7 mmol, 2 eq.) or Cs_2CO_3 (228.1 mg, 0.7 mmol, 2 eq.)] and benzene (2.5 mL). The vials were removed from the glovebox and either set to stir at 130°C or under irradiation with a 456 nm Kessil lamp. After 5 min under the conditions, the **11**/THF solution was added *via* syringe (0.4 mL, *ca.* 17 mol%), and allowed to react for 24 h. Workup and analysis were as described in general procedure A. *Note: for the irradiated reaction, initiator was added while the solution was being irradiated behind a screen.*

Entry	Initiator batch	Base	Conditions	Dodecane (mg)	% Yield			
					6	7	8	9
1	1	KO^{t}Bu	130°C	10.0	54.7	0.6	1.2	43.1
2	2	Cs_2CO_3	130°C	10.0	77.1	0.1	0.1	18.7
3	2	Cs_2CO_3	456 nm	10.0	76.9	<0.1	0.2	20.1

Table S17 –

Light on/off experiment

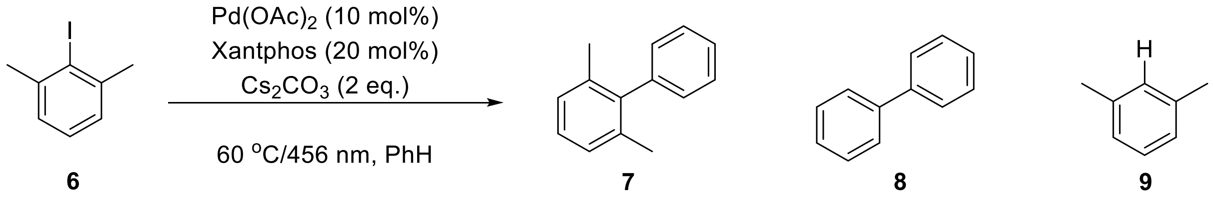

A 20 mL oven-dried microwave vial was cycled into a glovebox. To this was added **6** (101 μL, 0.7 mmol, 1 eq.), $Pd(OAc)_2$ (15.7 mg, 0.07 mmol, 0.1 eq.), Xantphos (81.0 mg, 0.14 mmol, 0.2 eq.), Cs_2CO_3 (456.1 mg, 1.4 mmol, 2 eq.) and benzene (5 mL). The vial was capped, removed from the glovebox and stirred in a oil bath set to 60°C. After stirring for 1 h, stirring was stopped and insolubles were allowed to settle before 50 μL of the solution was removed and quenched into 1 mL of an *n*-dodecane solution (6.5 mg *n*-dodecane in 15 mL of EtOAc, 0.433 mg per sample). The reaction flask was then placed under irradiation for 30 min after which another 50 μL aliquot was removed and quenched. The on/off cycle was repeated for the time intervals given below. Each quenched aliquot was analysed by GC-FID to obtain yields of each product, provided below (Table S18). Columns in blue indicate intervals of irradiation.

The period after 24 hours compares well to the standard assay reaction (Table S1, entry 8), but has been excluded from graphs for clarity of the steps.

		Time (h)									
		**0.00**	**1**	**1.5**	**2**	**2.75**	**3.5**	**4.25**	**5**	**5.75**	**24**
**Compound % yield**	**Iodoxylene 6**	100.0	98.9	57.5	56.8	45.0	44.1	33.7	33.3	28.9	6.1
	**Dimethylbiphenyl 7**	0.0	0.2	2.5	2.5	3.0	3.0	3.3	3.3	3.5	5.1
	**Biphenyl 8**	0.0	0.3	9.2	9.1	12.6	12.8	15.0	15.0	16.8	26.5
	**Xylene 9**	0.0	0.3	23.1	23.5	31.4	31.5	35.1	35.7	41.0	63.0
	**Ratio 8:7**		1.4	3.7	3.7	4.2	4.2	4.6	4.6	4.8	5.2

Table S18 –

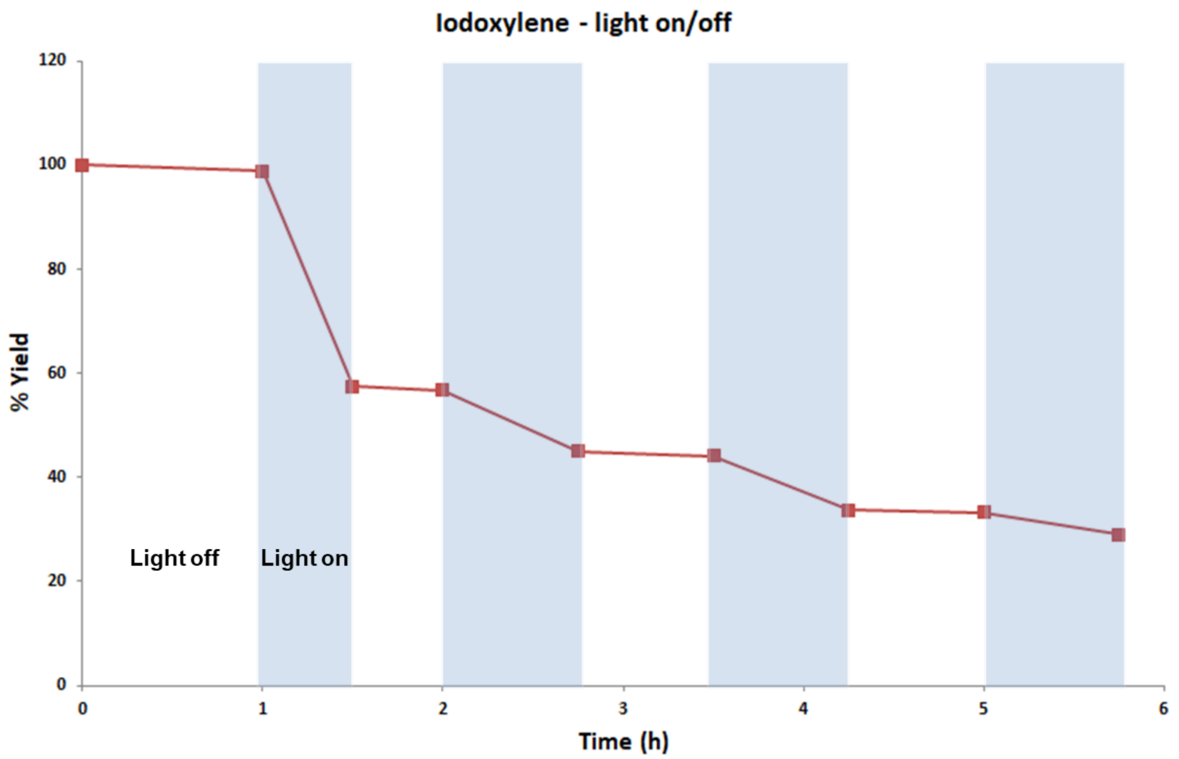

Figure S15

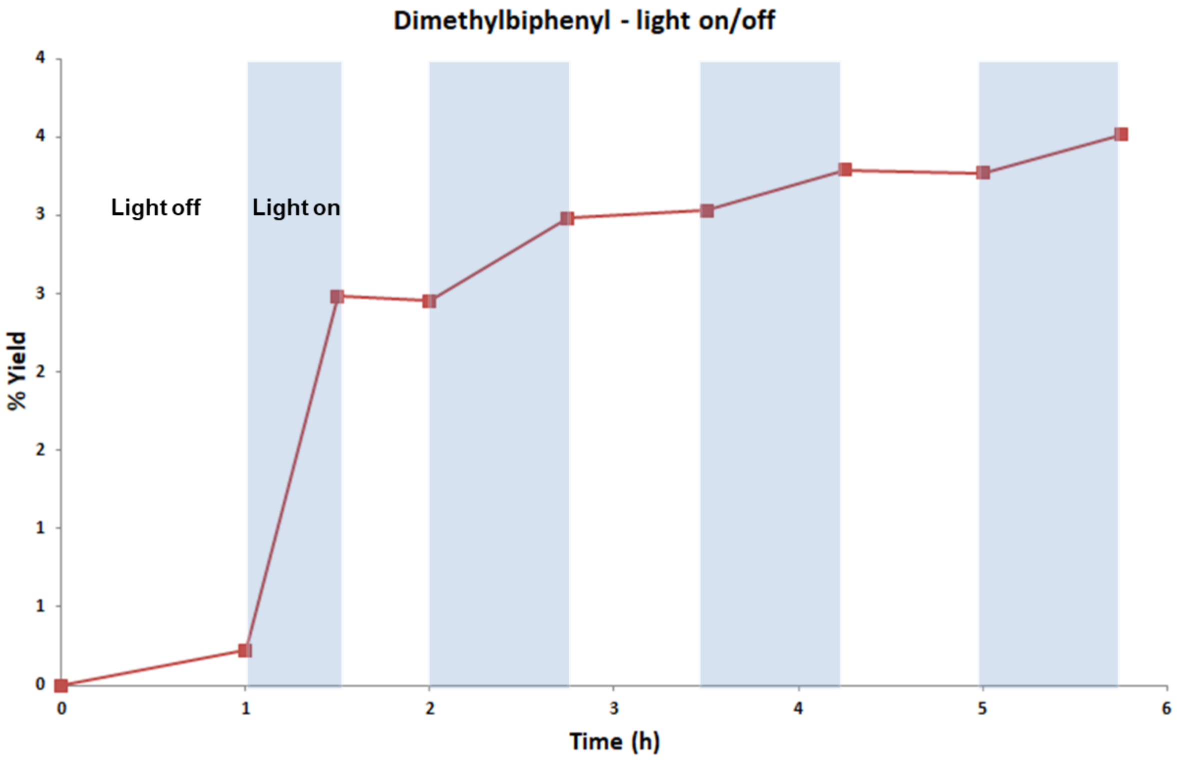

Figure S16

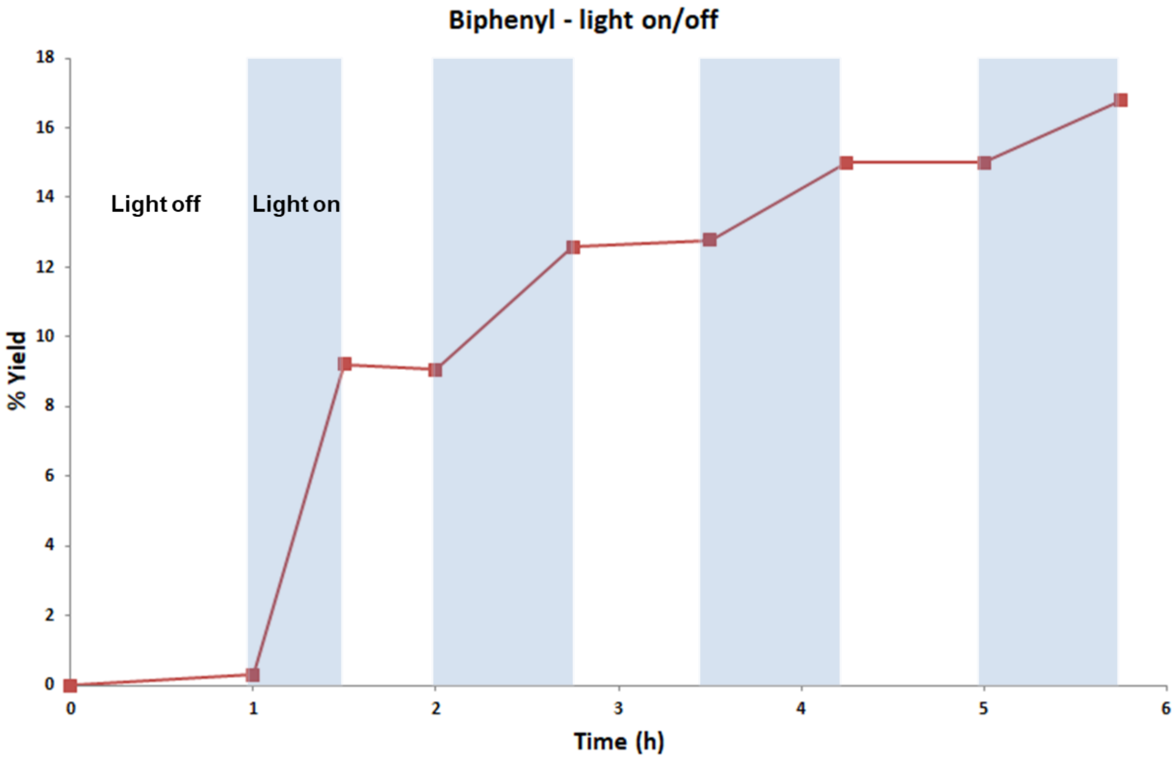

Figure S17

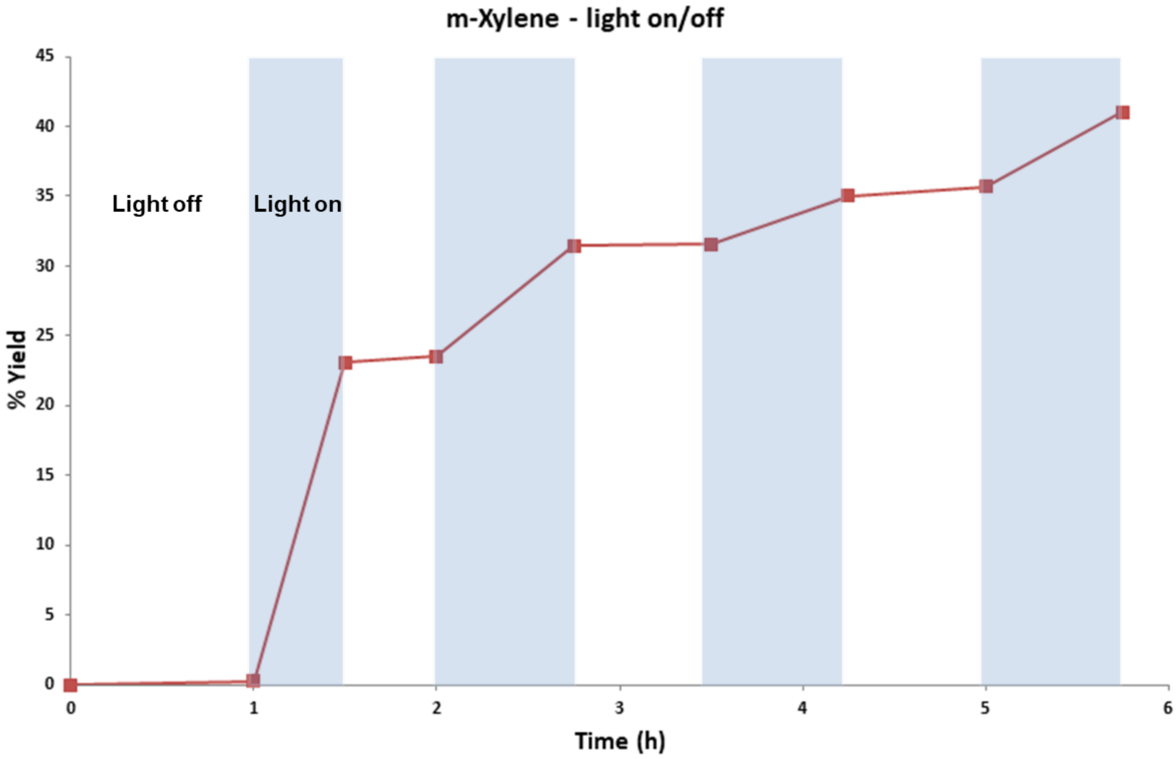

Figure S18

Determining quantum yield for reaction of 2-iodo-*m*-xylene and benzene in the presence of $Pd(OAc)_2$/Xantphos catalyst under 410 nm photoirradiation

Calculation of the photon flux

The photon flux of a Cary Eclipse spectrofluorometer was determined using a previously reported ferrioxalate actinometer.[4-6] A 0.006 M solution of potassium ferrioxalate trihydrate (147.4 mg) dissolved in 0.1 M H_2SO_4 (50 mL) was prepared. Separately, 1,10-phenanthroline (50 mg) and sodium acetate (11.25 g) were dissolved in 0.5 M H_2SO_4 (50 mL) to produce a buffered phenanthroline solution. A portion of the ferrioxalate solution (2 mL) was added to a 1 cm path length quartz cuvette and irradiated at 410 nm (20 nm slit width) for 5 minutes using the Xe lamp of the Cary Eclipse spectrofluorometer. The solution was stirred during irradiation. An aliquot of the buffered phenanthroline solution (0.35 mL) was then added and left for 1 hour to complex the liberated Fe^{2+}. A non-irradiated sample was prepared at the same time. The absorbance spectra were collected and the change in absorbance at 510 nm (*ΔA*) between the irradiated and non-irradiated sample were recorded. This was repeated 3 times and the mean value taken (*ΔA* = 0.2568).

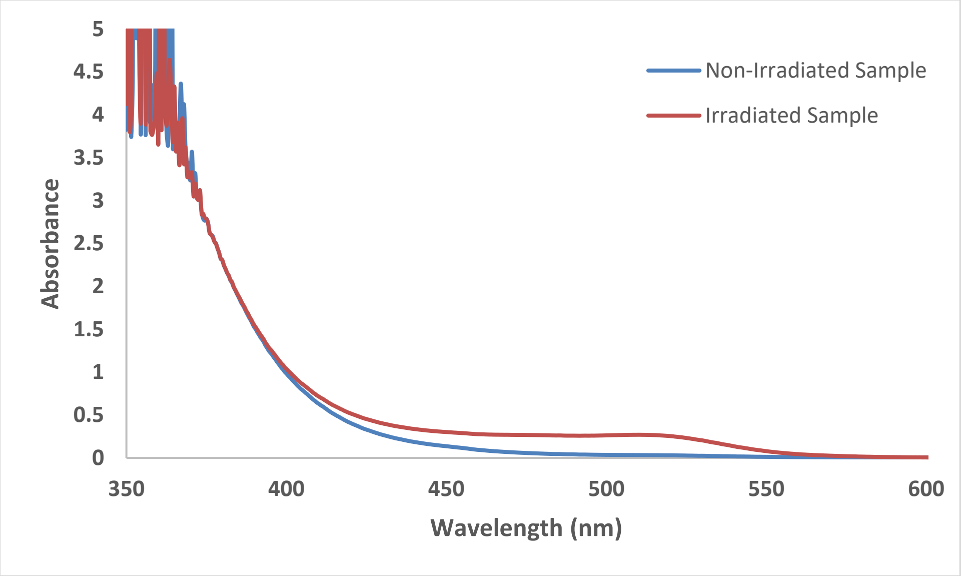

Figure S19 – Example absorption spectra of the irradiated (410 nm) and non-irradiated ferrioxalate actinometer samples

The conversion of ferrioxalate to phenanthroline complex (*amount of Fe*$^{2+}$) was calculated using the equation below:

$$amount\ of\ Fe^{2+} = \frac{V \times \Delta A}{\mathrm{l} \times \varepsilon} = \frac{0.00235\ \mathrm{L} \times\ 0.2568}{1\ \mathrm{cm}\ \times\ 11100\ \mathrm{L\ mol^{-1}\ cm^{-1}}} = 5.44\ \times 10^{-8}\ \mathrm{mol}$$

Where, *V* is the volume (0.00235 L), ΔA is the difference in absorbance at 510 nm between the irradiated and non-irradiated solutions, *l* is the path length (1 cm), and *ε* is the molar absorptivity at 510 nm of the phenanthroline complex (11,100 L mol^{-1} cm^{-1}).[5]

The absorbance of the dark ferrioxalate solution at 410 nm was measured to be 1.007 (Figure S19). The fraction of light absorbed (*f*) was calculated using the below equation:

$$f = 1 - 10^{-A} = 1 - 10^{-1.007} = 0.9016$$

The photon flux was calculated using the below equation:

$$Photon\ flux = \frac{amount\ of\ Fe^{2+}}{\Phi_{standard} \times t \times f} = \frac{5.44 \times 10^{-8}\ \mathrm{mol}}{1.13 \times 300 \times 0.9016} = 1.78 \times 10^{-10}\ \mathrm{einstein\ s^{-1}}$$

Where $\Phi_{Standard}$ is the quantum yield for the ferrioxalate actinometer (1.13 for a 0.006 M solution at λ = 405 nm, approximated to be the same at 410 nm),[5] *t* is the time (300 s), and *f* is the fraction of light absorbed at λ = 410 nm (0.9016). The photon flux was calculated to be 1.78×10^{-10} einstein s^{-1} .

Determination of quantum yield

In a glovebox, solutions of $Pd(OAc)_2$ with 2 equivalents of Xantphos were made up in benzene at two different concentrations, 1.4 mM (0.1x reaction conc.) and 0.14 mM (0.01x reaction conc.), and the absorbance measured. At 1.4 mM the absorbance at 410 nm is >4, thus giving an *f* value of *ca.* 0.9999 making it suitable for determination of quantum yield (Figure S20).

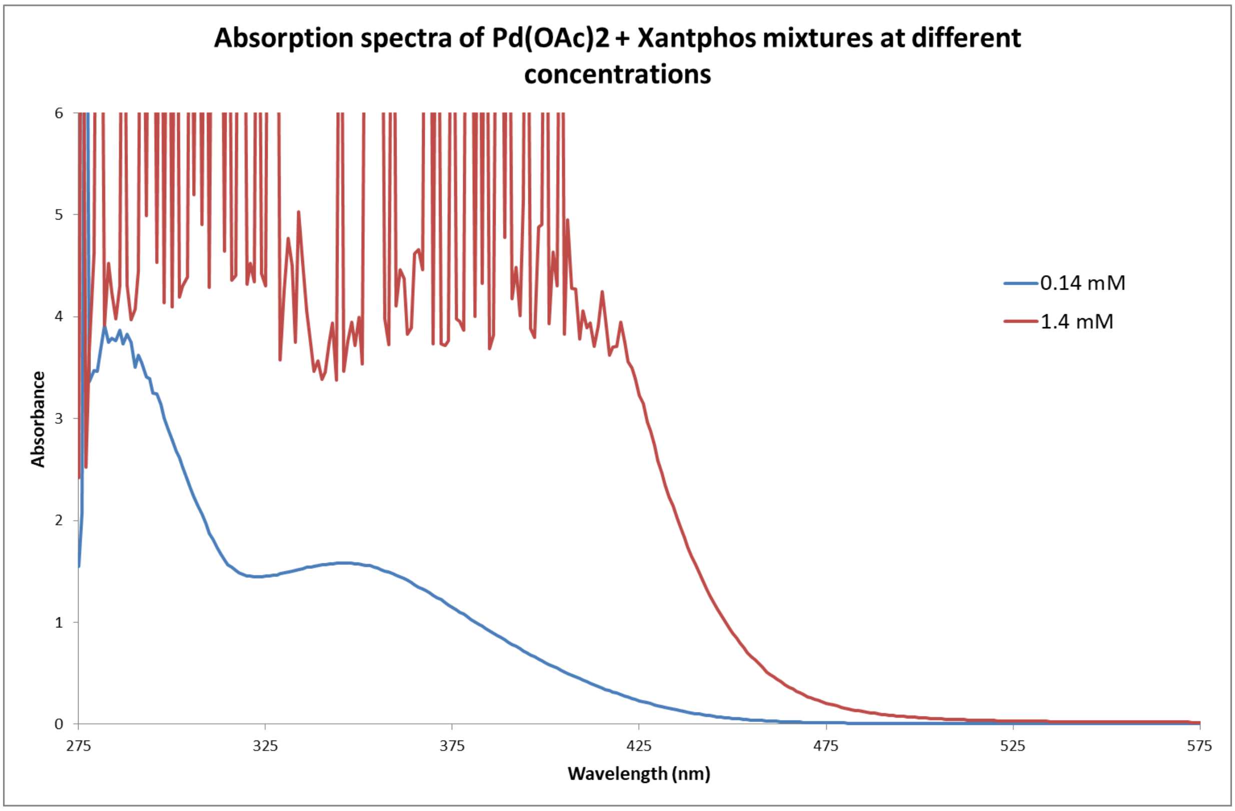

Figure S20 – Absorption spectra of mixtures of $Pd(OAc)_2$ and Xantphos at different concentrations of palladium.

To measure the quantum yield of the reaction, in the glovebox a vial was charged with $Pd(OAc)_2$ (1.6 mg, 0.007 mmol, 0.1 eq.), Xantphos (8.1 mg, 0.014 mmol, 0.2 eq.), iodoxylene **6** (10.1 μL, 0.07 mmol, 1 eq.) and benzene (5 mL). After mixing, half of this solution (2.5 mL) was added to a screw-top cuvette charged with Cs_2CO_3 (22.8 mg, 0.14 mmol, 2 eq.) and a small 'V-shaped' stirbar. The reaction was then irradiated at 410 nm (20 nm slit width) for 3 hours under identical conditions to the ferrioxalate actinometer whilst stirring. After this time, the cuvette was opened to air and *n*-dodecane (4.6 mg) in EtOAc (2 mL) was added. The solution was mixed thoroughly, then an aliquot diluted in EtOAc and analysed by GC-FID. The combined yield of 2,6-dimethylbiphenyl (**7**) and *m*-xylene **9** was calculated as 0.000508 mmol.

The quantum yield *Φ* of this reaction was calculated using the equation below:

$$\phi = \frac{mol\,product}{photon\,flux \times t \times f} = \frac{0.508\ \times 10^{-6}\,\mathrm{mol}}{1.78 \times 10^{-10}\,\mathrm{einstein\ s^{-1}}\ \times 10800\,\mathrm{s}\ \times 1} =\ 0.26$$

Where *mol product* is the moles of product, *photon flux* is the flux of the lamp at 410 nm (with 20 nm slit widths), *t* is the irradiation time for the reaction (10800 s) and *f* is the fraction of light absorbed (assumed to be 1). A quantum yield of 0.26 makes it unlikely that a chain mechanism is in operation.

14.0 NMR spectra

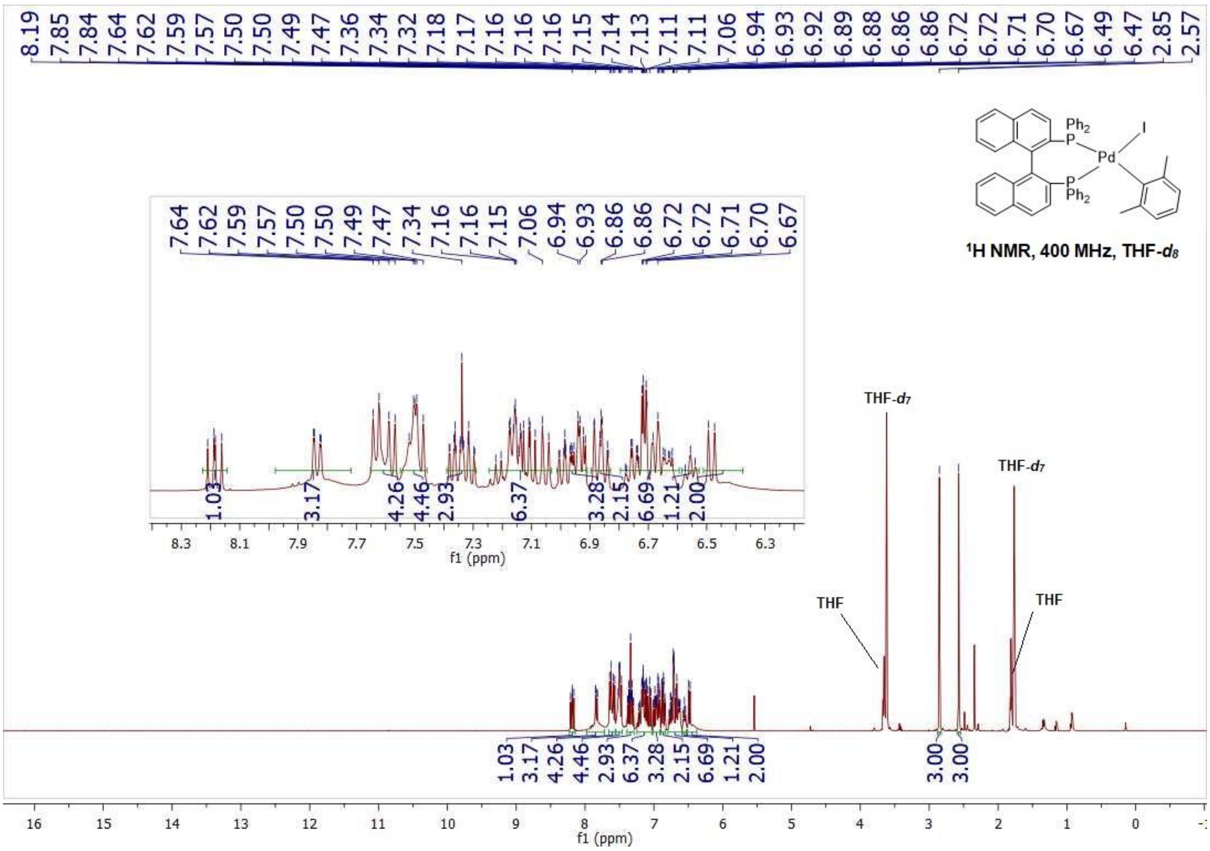

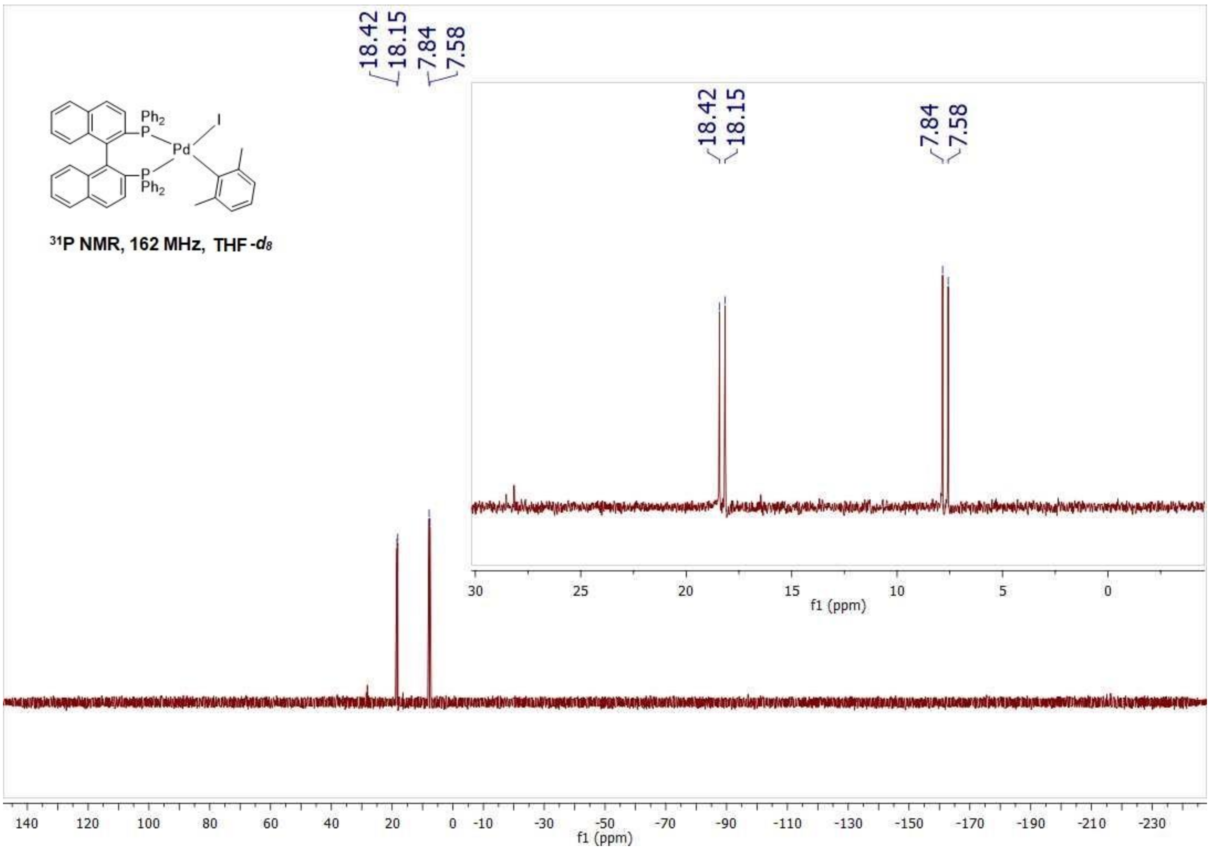

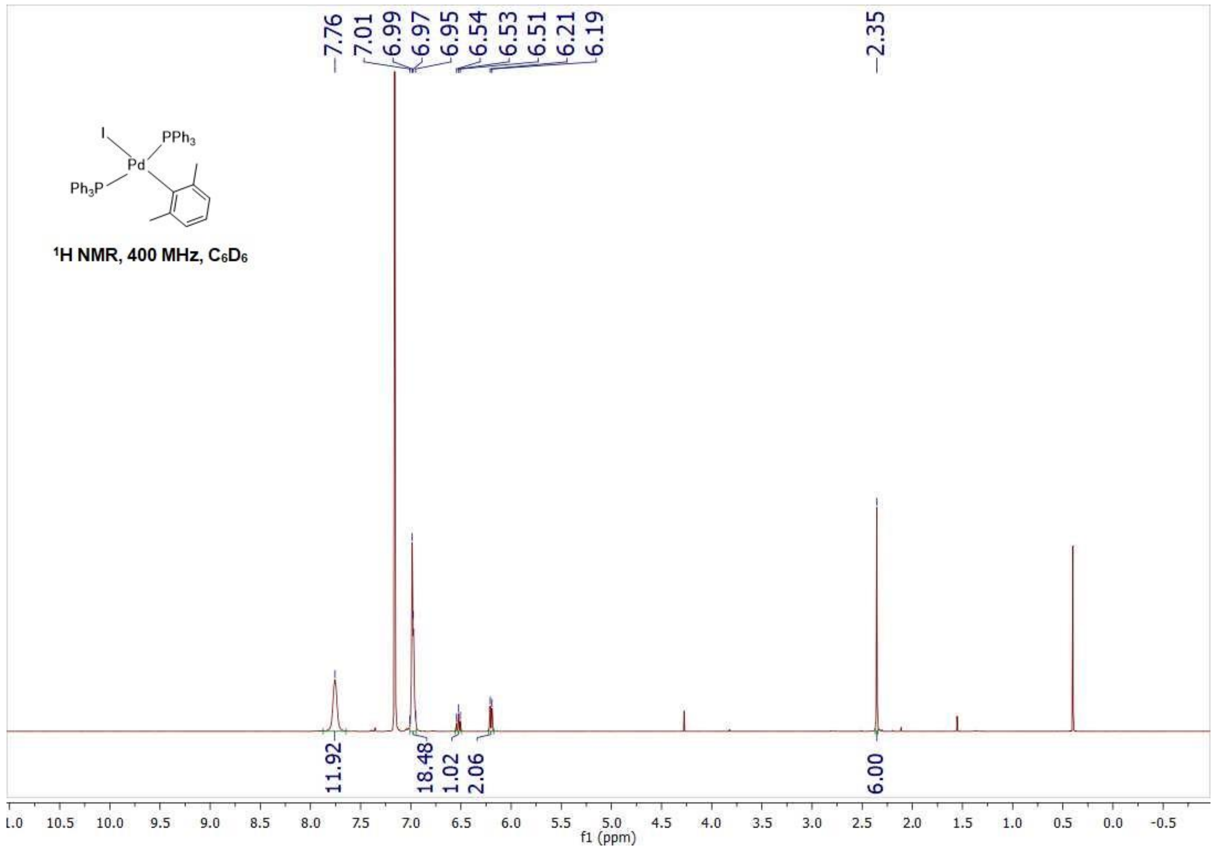
7.76
7.01
6.99
6.97
6.95
6.54
6.53
6.51
6.21
6.19
2.35
I
PPh3
Pd
Ph3P
1H NMR, 400 MHz, C6D6
11.92
18.48
1.02
2.06
6.00
f1 (ppm)

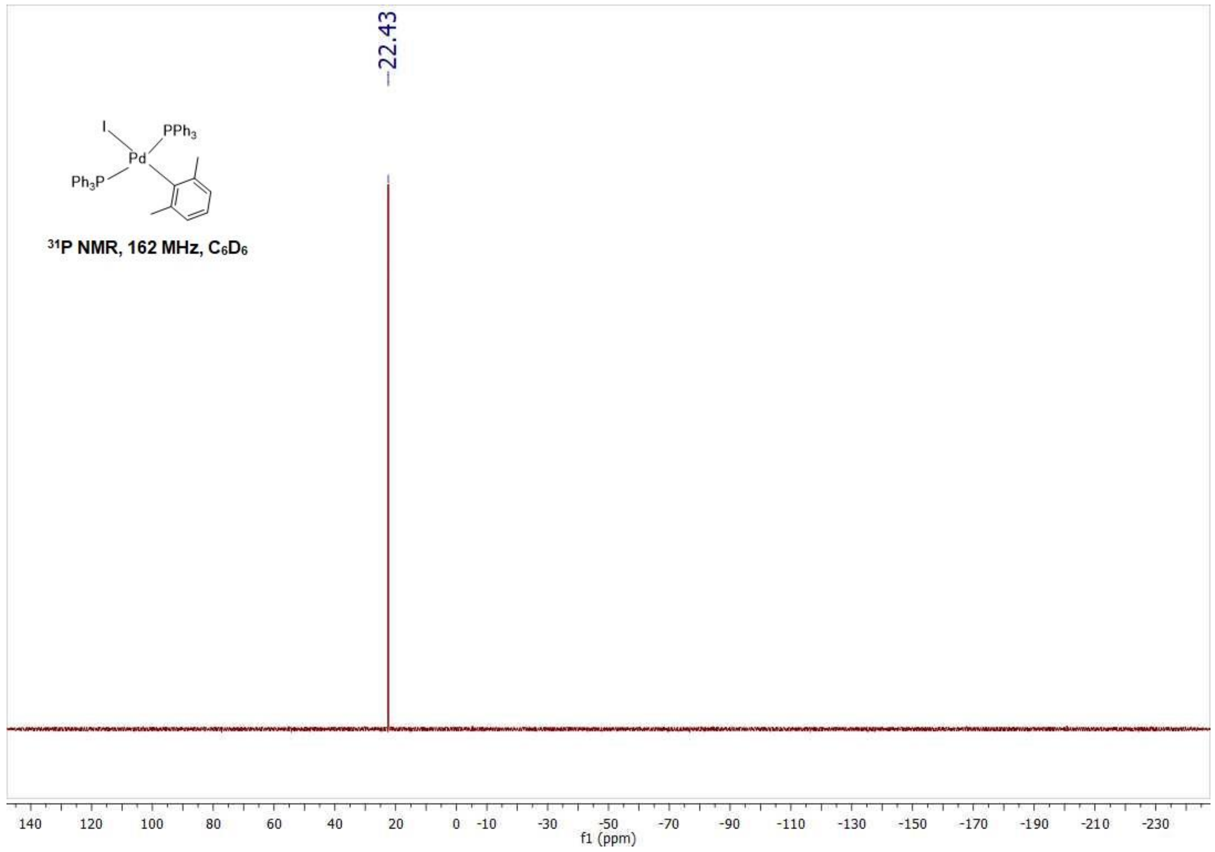
22.43
I
PPh3
Pd
Ph3P
31P NMR, 162 MHz, C6D6
f1 (ppm)

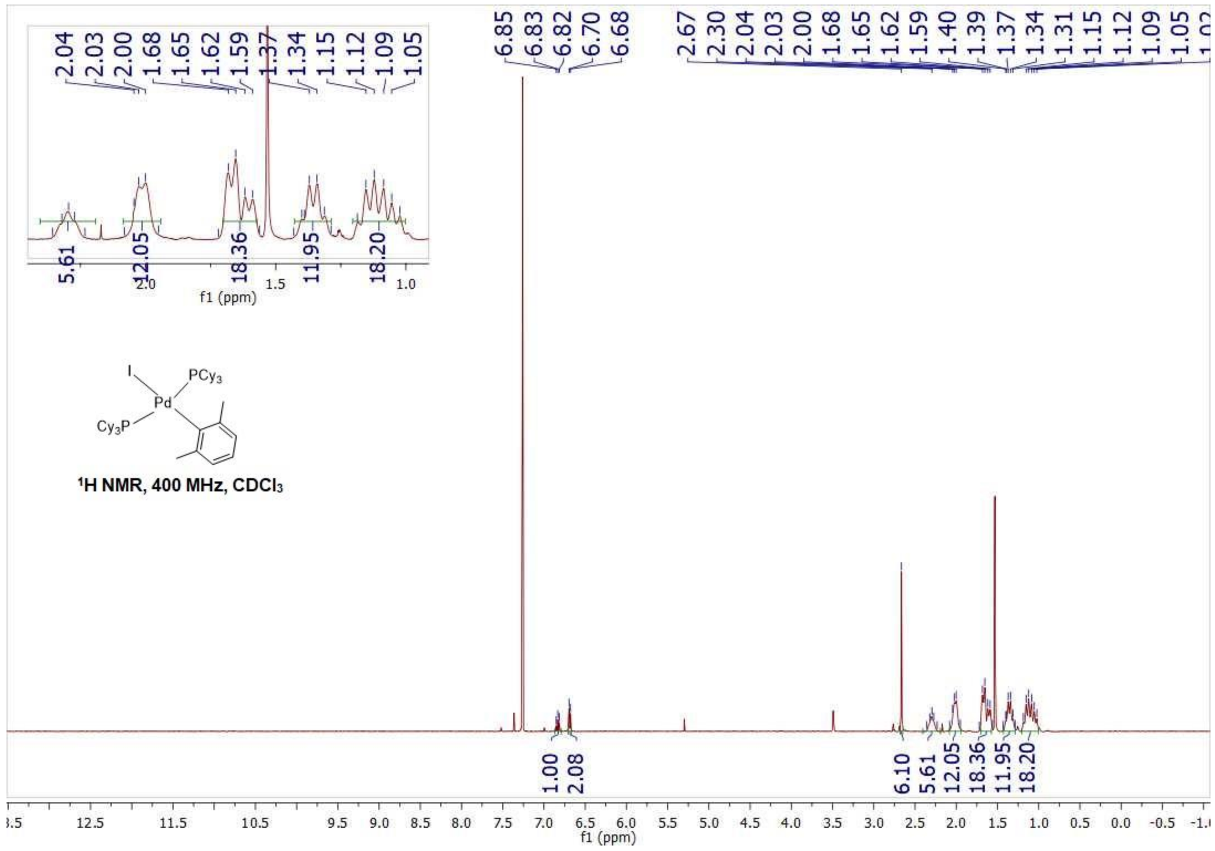
2.04 2.03 2.00 1.68 1.65 1.62 1.59 1.37 1.34 1.15 1.12 1.09 1.05
5.61 12.05 18.36 11.95 18.20
f1 (ppm)
6.85 6.83 6.82 6.70 6.68
2.67 2.30 2.04 2.03 2.00 1.68 1.65 1.62 1.59 1.40 1.39 1.37 1.34 1.31 1.15 1.12 1.09 1.05
I
PCy3
Pd
Cy3P
¹H NMR, 400 MHz, CDCl3
1.00 2.08
6.10 5.61 12.05 18.36 11.95 18.20
f1 (ppm)

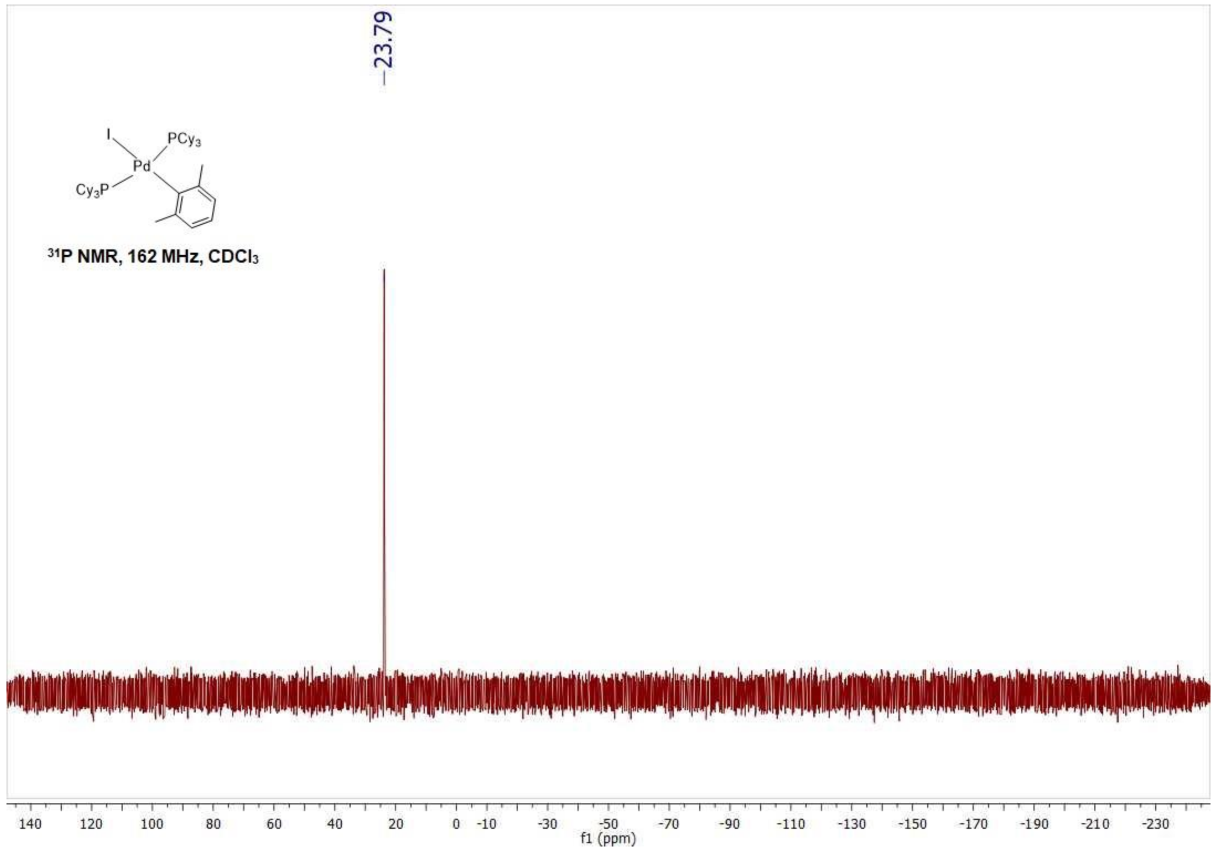
23.79
I
PCy3
Pd
Cy3P
³¹P NMR, 162 MHz, CDCl3
f1 (ppm)

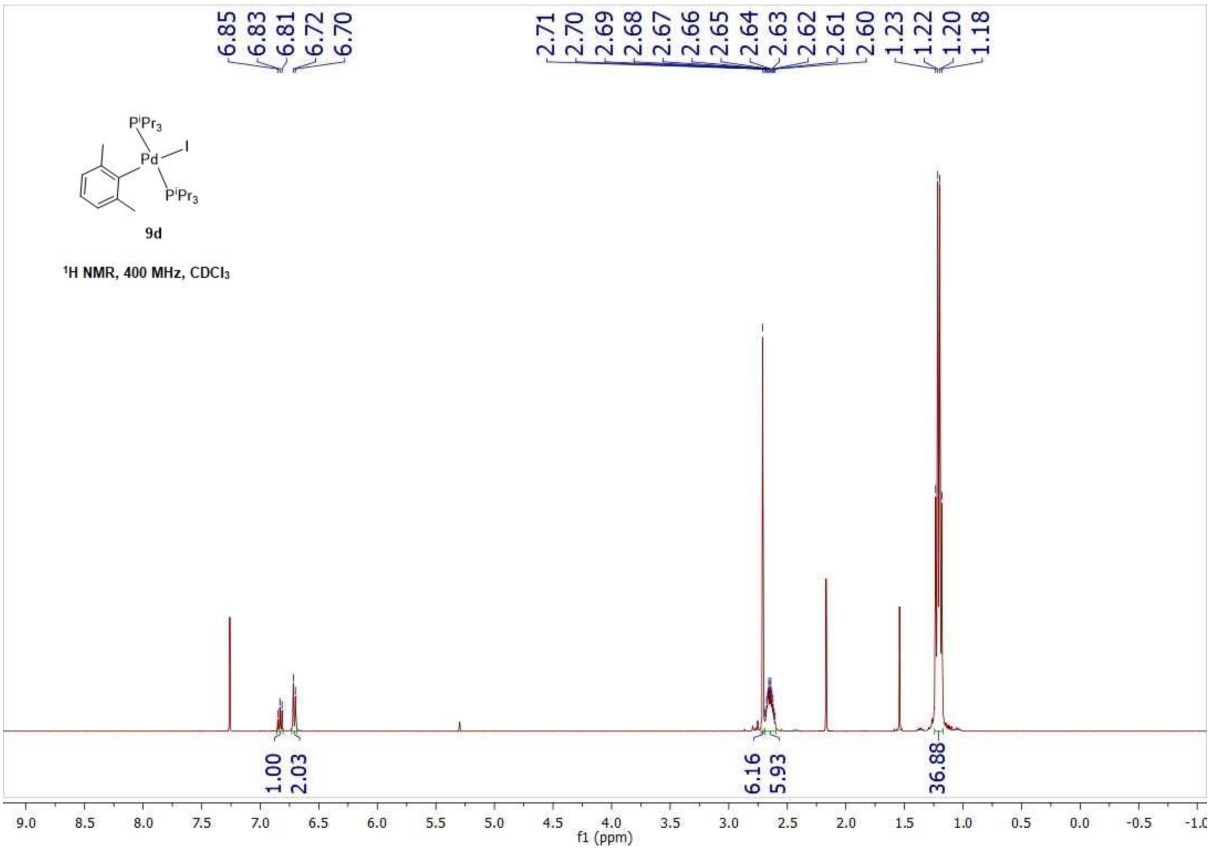
6.85 6.83 6.81 6.72 6.70
2.71 2.70 2.69 2.68 2.67 2.66 2.65 2.64 2.63 2.62 2.61 2.60 1.23 1.22 1.20 1.18
P^iPr_3
Pd
I
9d
^1H NMR, 400 MHz, $CDCl_3$
1.00 2.03
6.16 5.93
36.88
9.0 8.5 8.0 7.5 7.0 6.5 6.0 5.5 5.0 4.5 4.0 3.5 3.0 2.5 2.0 1.5 1.0 0.5 0.0 -0.5 -1.0
f1 (ppm)

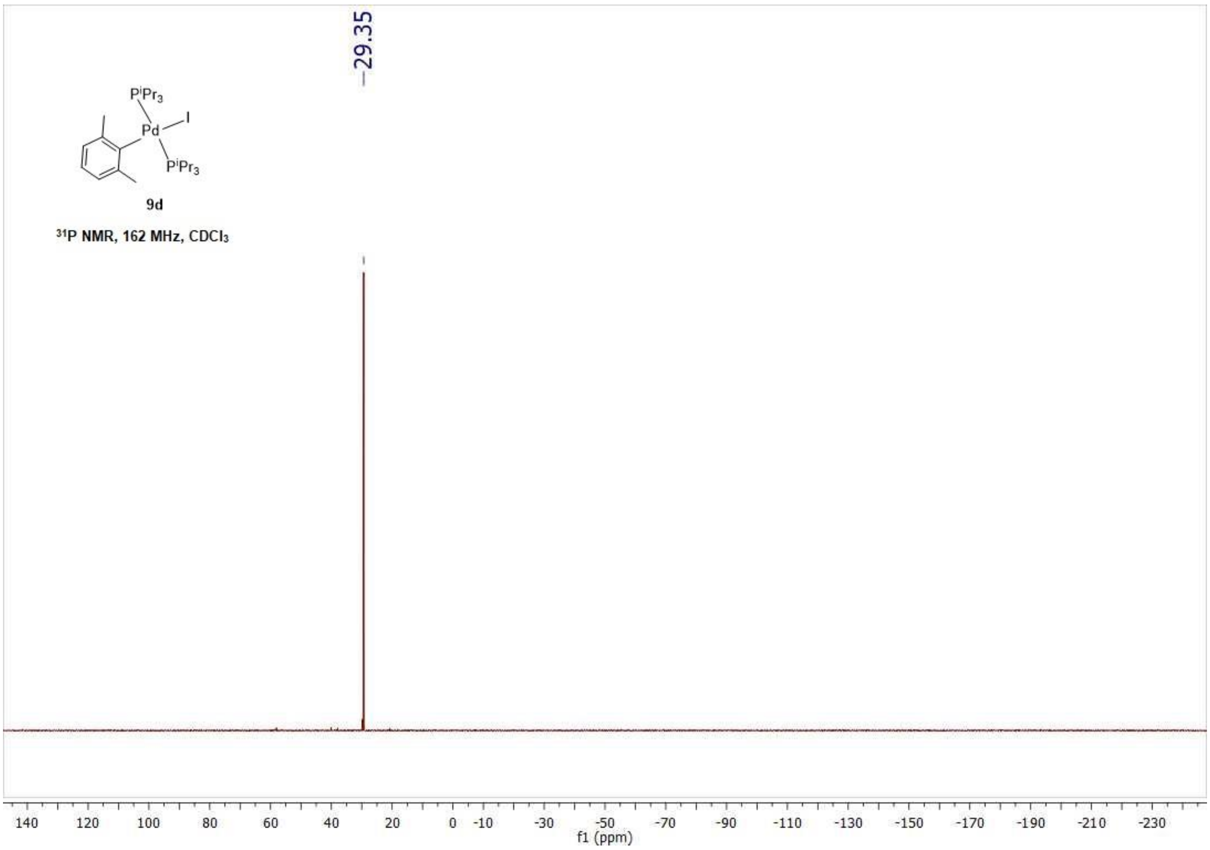
29.35
P^iPr_3
Pd
I
9d
^{31}P NMR, 162 MHz, $CDCl_3$
140 120 100 80 60 40 20 0 -10 -30 -50 -70 -90 -110 -130 -150 -170 -190 -210 -230
f1 (ppm)

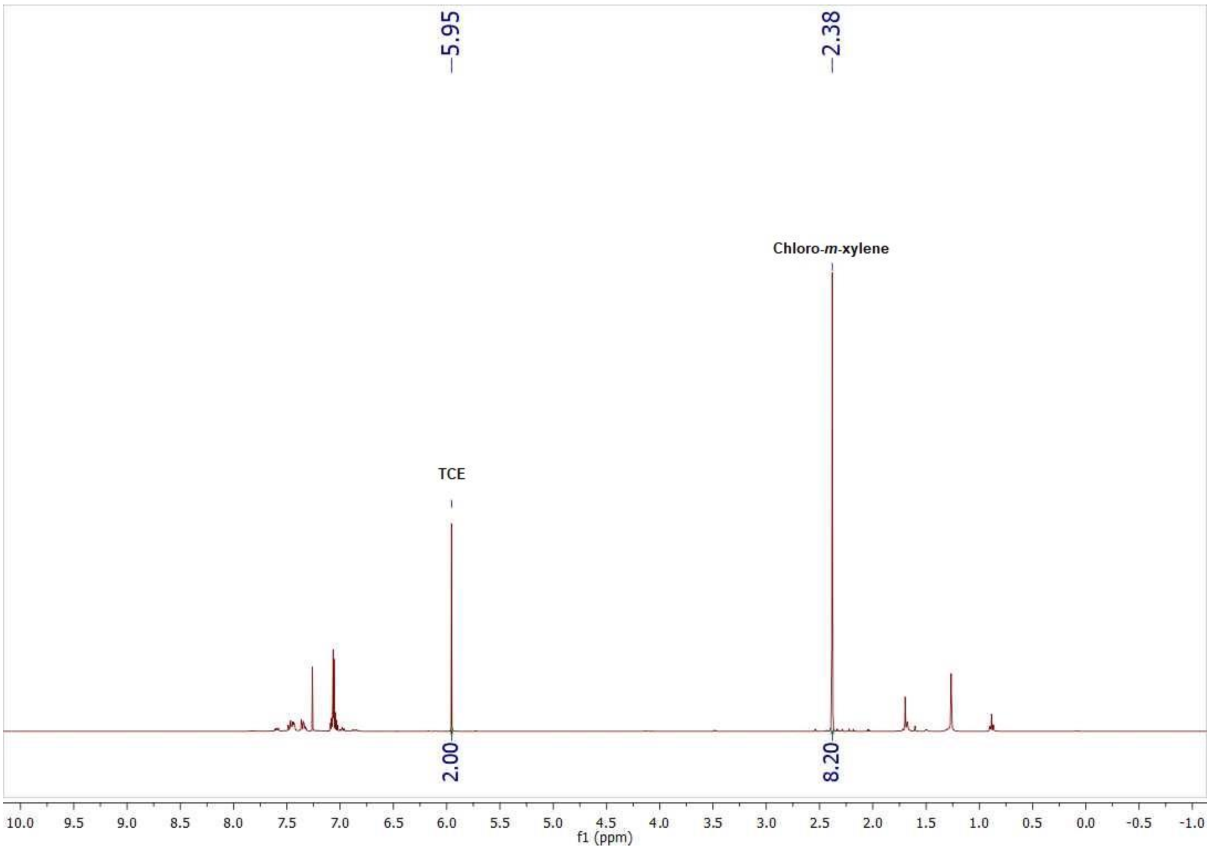

^{1}H NMR spectra to calculate yield of unreacted chloro-m-xylene, Table S4 entry 2, using 38.7 mg of TCE.

15.0 X-Ray data

Crystallographic measurements were made with monochromatic Cu radiation (λ = 1.54184 Å) using a Rigaku Synergy-i diffractometer. Raw data processing utilised the program CrysalisPro.[7] The structure was solved using direct methods and was refined against F^2 to convergence using all unique reflections and the program Shelxl,[8] as implemented within WinGX.[9] The THF solvent of crystallisation was found to be disordered, see deposited cif for details. The disordered solvent was modelled across two sites and appropriate restraints and constraints were added to ensure approximation to normal geometry and displacement behaviour. Selected crystallographic and refinement parameters are given in Table S19 and full information in cif format has been deposited with the CCDC as reference number 2380109.

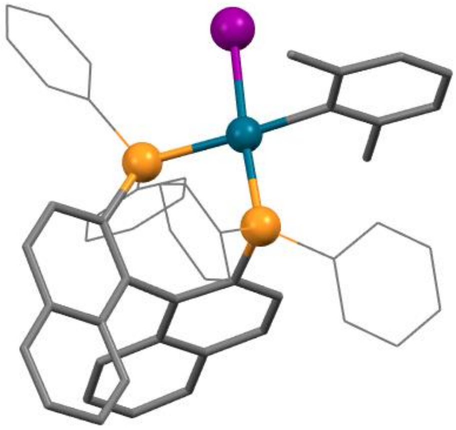

Molecular structure of (*rac*-BINAP)Pd(xylyl)I **14b** from single-crystal X-ray diffraction. Drawn to emphasise the geometry of the BINAP group. H atoms and the disordered THF solvate have been omitted for clarity.

Table S19. Selected Crystallographic Parameters.

**Compound**	**14b**
**CCDC ref. no.**	2380109
**Formula**	$C_{56}H_{49}IOP_2Pd$
**Form. Wt.**	1033.19
**Crystal system**	Monoclinic
**Space group**	$P2_1/n$
**Temp. (K)**	100(2)
**a (Å)**	12.7484(1)
**b (Å)**	17.2481(2)
**c (Å)**	20.4293(2)
**α (°)**	90
**β (°)**	92.348(1)
**γ (°)**	90
**Volume (Å^3)**	4488.34(8)
**Z**	4
**Collected Reflections**	41505
**Independent Reflections**	8464
**Observed Reflections**	7644
**Rint**	0.0860
**2Θmax (°)**	140
**No. Parameters**	559
**S**	1.122
**R [on *F*, obs refs only]**	0.0509
**ωR [on *F*^{2}, all data]**	0.1466

16.0 GCFID Calibration curves

These calibration curves are the same used in our previous study.[10] To obtain yields for mixtures of compounds **6, 7**, **8, 9** and bromo-*m*-xylene, calibration curves were constructed for each compound by GC-FID using *n*-dodecane as an internal standard. Authentic samples of compounds **6, 8**, **9** and bromoxylene were bought from commercial sources, while compound **7** was synthesised and calibrations corrected for homocoupled impurity 2,2',6,6'-tetramethyl-1,1'-biphenyl. To construct each calibration curve, known amounts of each compound (approximately 0.01 mmol – 0.1 mmol) were mixed with a known amount of dodecane (approximately 0.1 mmol). The mixtures were then diluted with EtOAc and analysed on the GC-FID to obtain the peak area for both the sample compound (SC) and dodecane internal standard (IS). Calibration curves were constructed by plotting:

$$\frac{mmol(SC)}{mmol(IS)} \text{ vs. } \frac{area\ (SC)}{area\ (IS)}$$

The plots are forced through the origin, with the gradient representing the response factor (RF) for each compound against *n*-dodecane. To obtain the composition of relevant reaction mixtures from these curves, a known mass of dodecane was added to the reaction mixture, an aliquot of which was then removed, diluted with EtOAc and analysed by GC-FID. The peak areas for each compound were then input into the following equation to obtain the moles of **6, 7, 8, 9** and bromoxylene:

$$\frac{area(SC)*mmol(IS)}{area(IS)*RF} = mmol(SC)$$

Entry	Mass of **9** (mg)	mmol of **9**	Mass dodecane (mg)	mmol dodecane	mmol ratio	Area of **9**	Area Dodecane	Area ratio
1	2.1	0.0198	18.8	0.1100	0.1800	12578.9	98487.2	0.1277
2	8.6	0.0810	17.6	0.1030	0.7864	42401.7	84773.8	0.5001
3	11.0	0.1040	17.9	0.1050	0.9904	18433.6	26890.0	0.6855
4	33.2	0.3130	18.2	0.1070	2.9252	32922.7	16190.1	2.0335
5	84.6	0.7970	17.4	0.1020	7.8137	40651.0	7618.8	5.3356
6	105.8	0.9970	17.2	0.1010	9.8712	36894.7	5652.2	6.5274

Table S20 *– Calibration curve data for m-xylene **9***

Entry	Mass of **8** (mg)	mmol of **8**	Mass dodecane (mg)	mmol dodecane	mmol ratio	Area of **8**	Area Dodecane	Area ratio
1	2.1	0.0136	15.2	0.0892	0.1524	9428.4	68957.4	0.1367
2	7.5	0.0486	14.4	0.0845	0.5751	33761.9	65096.8	0.5186
3	16.0	0.1040	14.7	0.0863	1.2050	19101.4	18326.8	1.0422

4	47.2	0.3060	14.7	0.0863	3.5457	34515.8	11200.1	3.0817
5	109.9	0.7130	15.6	0.0916	7.7838	37752.0	5400.0	6.9911

Table S21 *– Calibration curve data for biphenyl **8**.*

Entry	Mass of sample (mg)	mmol of **7**	Mass dodecane (mg)	mmol dodecane	mmol ratio	Area of **7**	Area Dodecane	Area ratio
1	2.0	0.0104	18.0	0.106	0.0982	6115.4	62881.5	0.0972
2	7.8	0.0406	19.9	0.117	0.3470	12765.3	36554.7	0.3492
3	15.3	0.0796	17.5	0.103	0.7733	16138	20688.2	0.7800
4	44.1	0.2296	18.9	0.111	2.0685	22237.2	10923.1	2.0357
5	94.5	0.4920	17.9	0.105	4.6857	32072.9	6931	4.6274

Table S22 *– Calibration curve data for 2,6-dimethylbiphenyl **7**, with correction for impurity.*

Entry	Mass of **6** (mg)	mmol of **6**	Mass dodecane (mg)	mmol dodecane	mmol ratio	Area of **6**	Area Dodecane	Area ratio
1	6.1	0.0263	16.4	0.0962	0.2734	11457.0	59542.4	0.1924
2	9.3	0.0401	17.1	0.100	0.4010	12907.3	48829.4	0.2643
3	21.7	0.0935	16.9	0.0992	0.9425	16799.3	27416.6	0.6127
4	50.8	0.219	16.4	0.0963	2.2741	27341.3	18276.5	1.4960
5	87.5	0.377	17.4	0.102	3.6961	29666.8	12433.8	2.3860
6	133.1	0.574	15.9	0.0933	6.1522	40482.1	10198.2	3.9695

Table S23 *– Calibration curve data for 2,6-dimethyliodobenzene **6.***

Entry	Mass of **bromoxylene** (mg)	mmol of **bromoxylene**	Mass dodecane (mg)	mmol dodecane	mmol ratio	Area of **bromoxylene**	Area Dodecane	Area ratio
1	7.3	0.0394	17.4	0.1022	0.3855	2752.4	10719.0	0.2568
2	13.8	0.0746	17.0	0.0998	0.7475	5208.3	10519.0	0.4951
3	23.7	0.1281	17.1	0.1004	1.2756	6220.2	7455.5	0.8343
4	55.7	0.3010	17.3	0.1016	2.9626	10238.0	5204.3	1.9672
5	130.2	0.7036	16.6	0.0975	7.2164	13561.0	2865.8	4.7320

Table S24 *– Calibration curve data for 2,6-dimethylbromobenzene.*

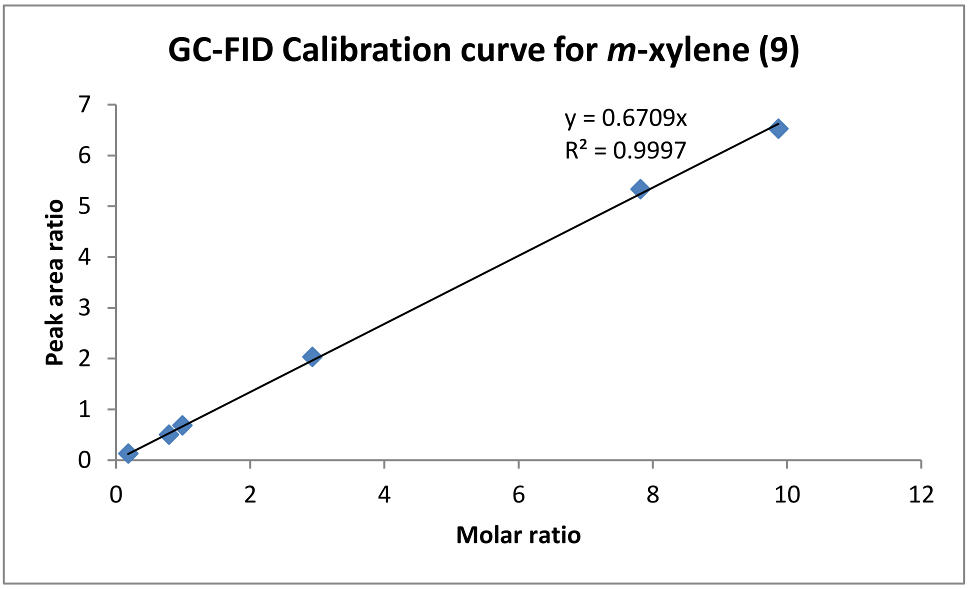

Figure S21 *– GC-FID calibration curve for m-xylene* ***9.***

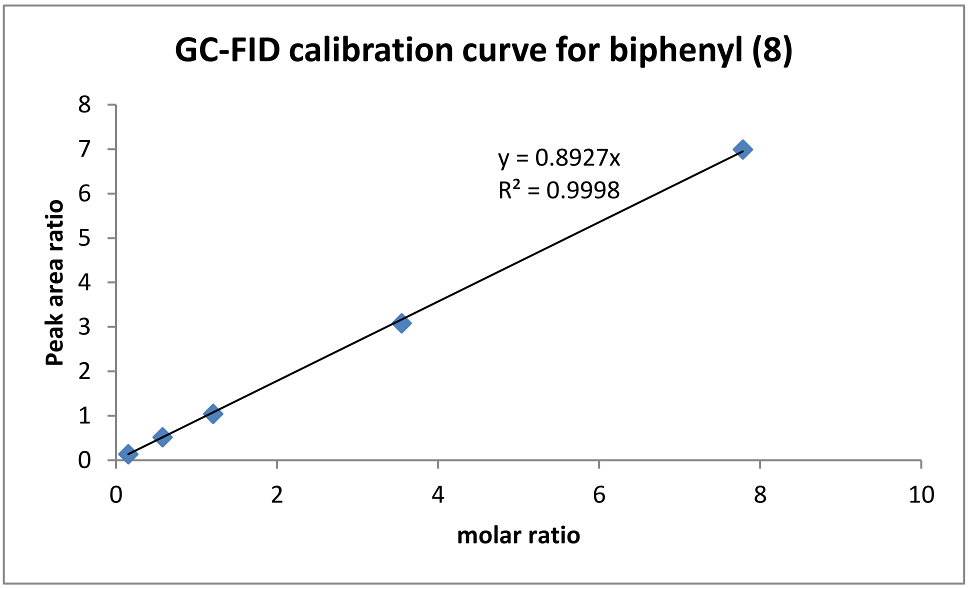

Figure S22 *– GC-FID calibration curve for biphenyl* ***8****.*

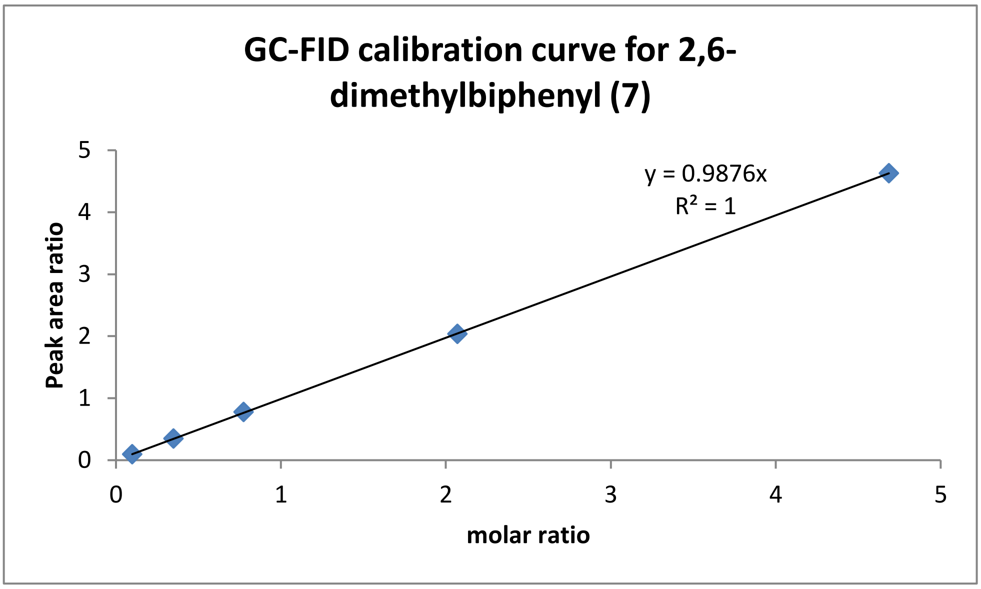

Figure S23 *– GC-FID calibration curve for 2,6-dimethylbiphenyl* ***7.***

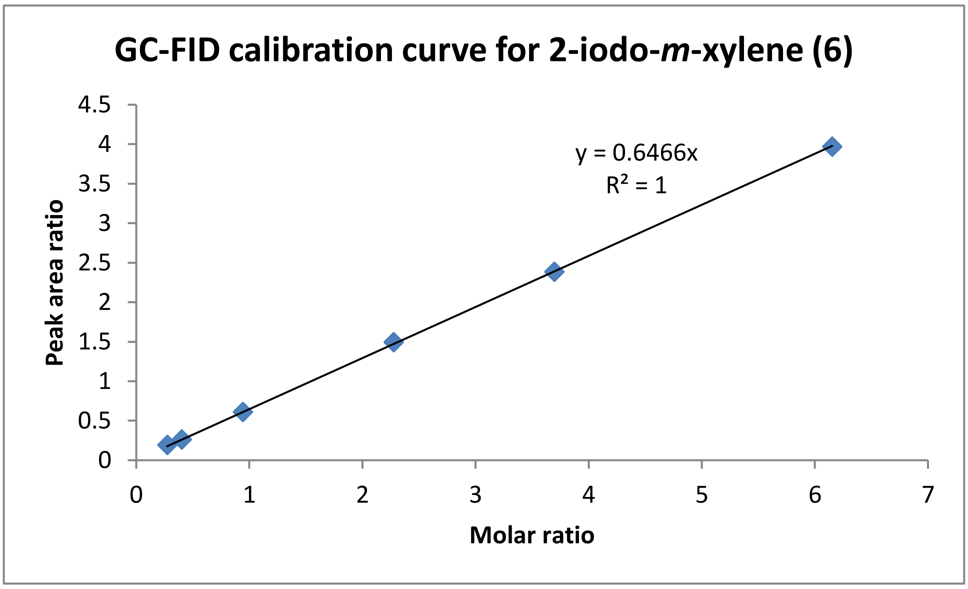

Figure S24*– GC-FID calibration curve for 2-iodo-m-xylene* ***6.***

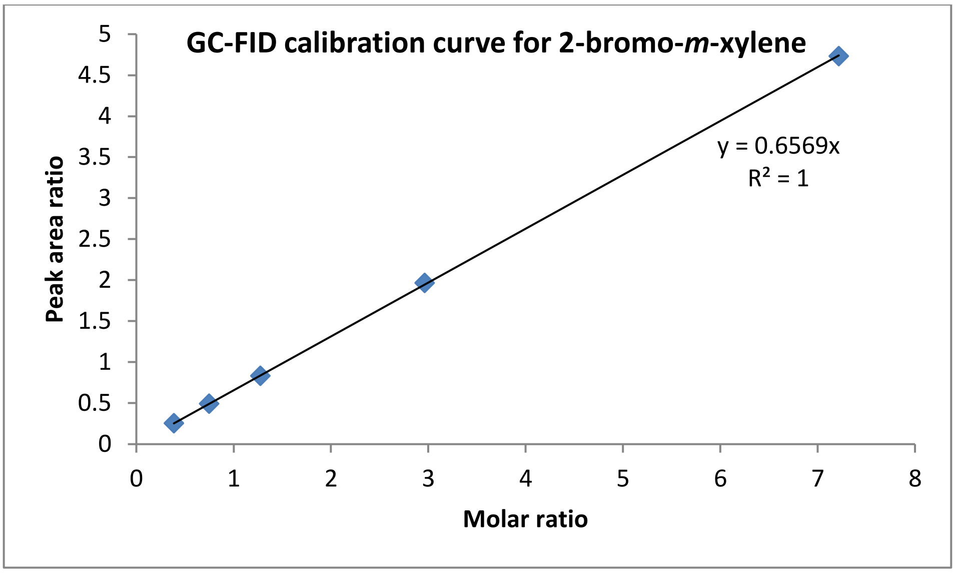

Figure S25– *GC-FID calibration curve for 2,6-dimethylbromobenzene*

16.0 Reaction GC-FID traces

Approximate retention times (RT) are as follows: iodoxylene **6** RT = 11.2 min, 2,6-dimethylbiphenyl **7** RT = 12.4 min, biphenyl **8** RT = 12.0 min, *m*-xylene **9** = 7.0 min, *n*-dodecane = 10.6 min, bromoxylene RT = 10.2 min.

GC-FID traces for Table S1

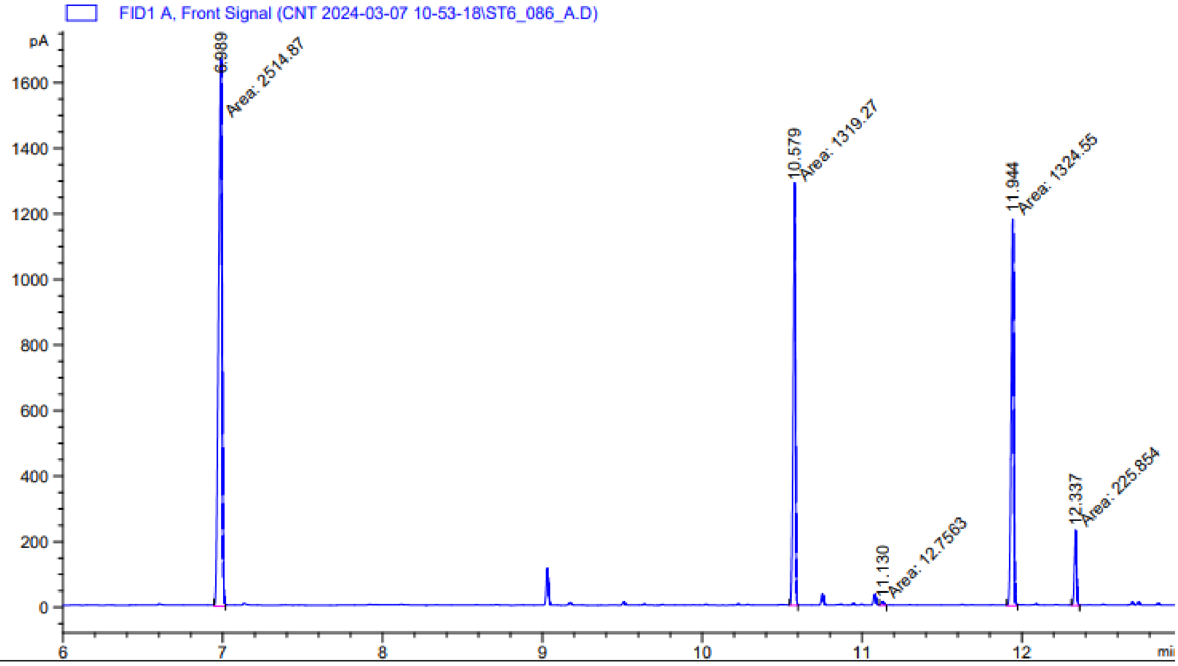

Entry 1

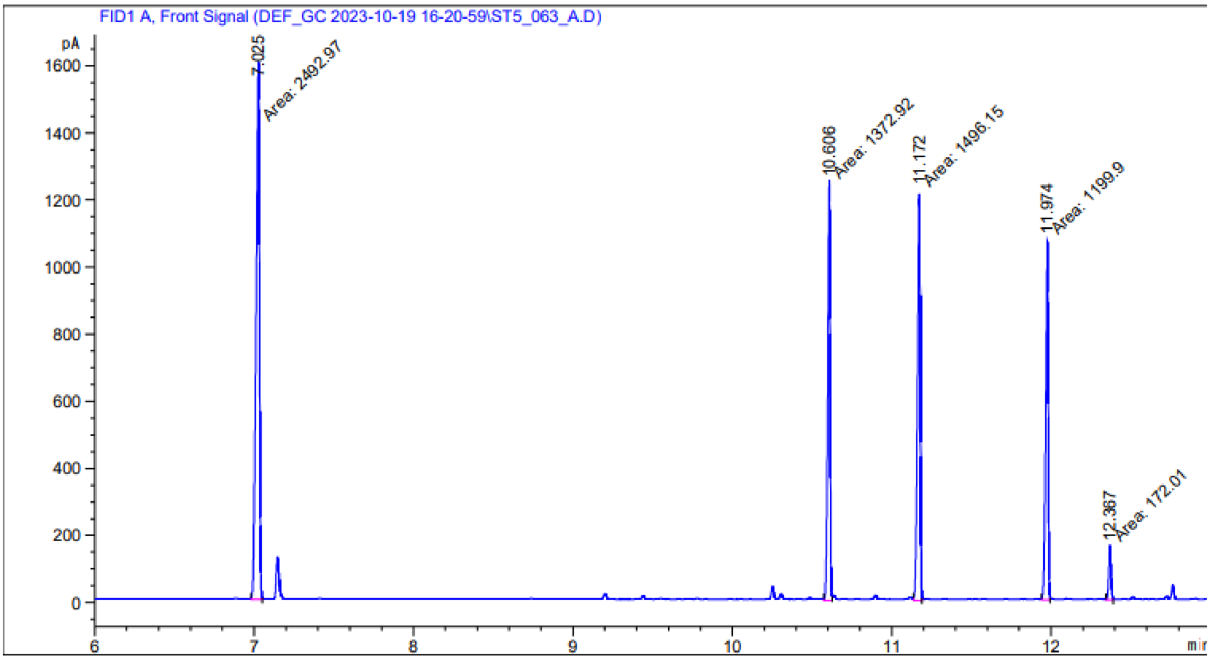

Entry 2 –

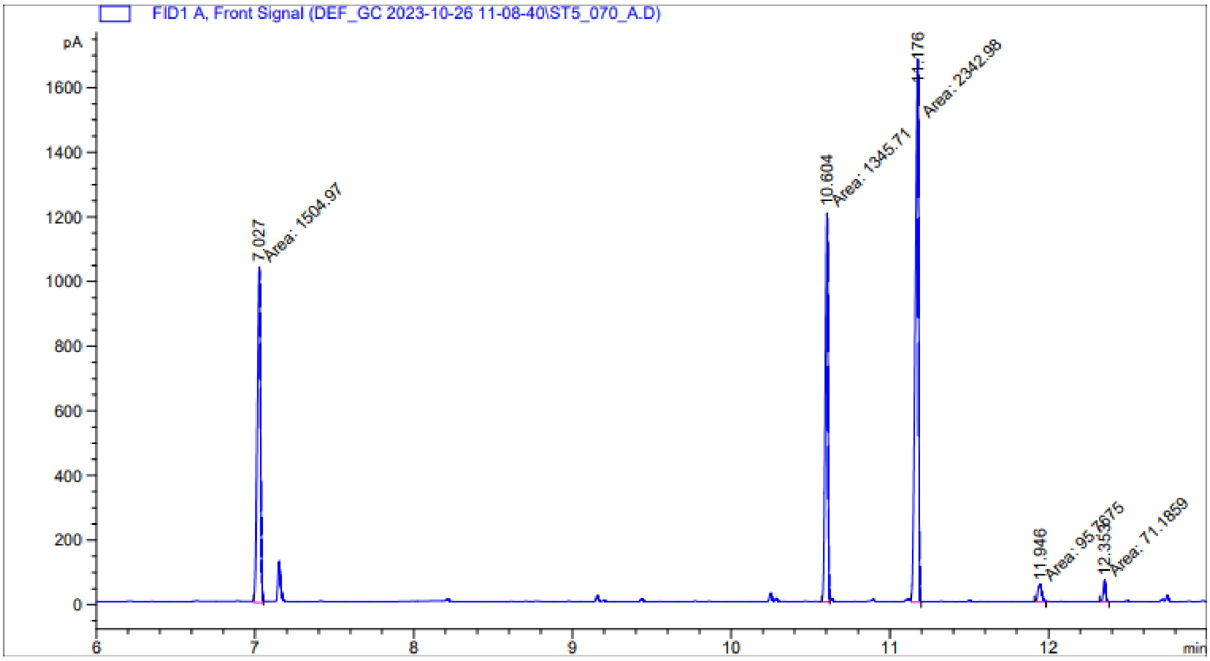

Entry 3 –

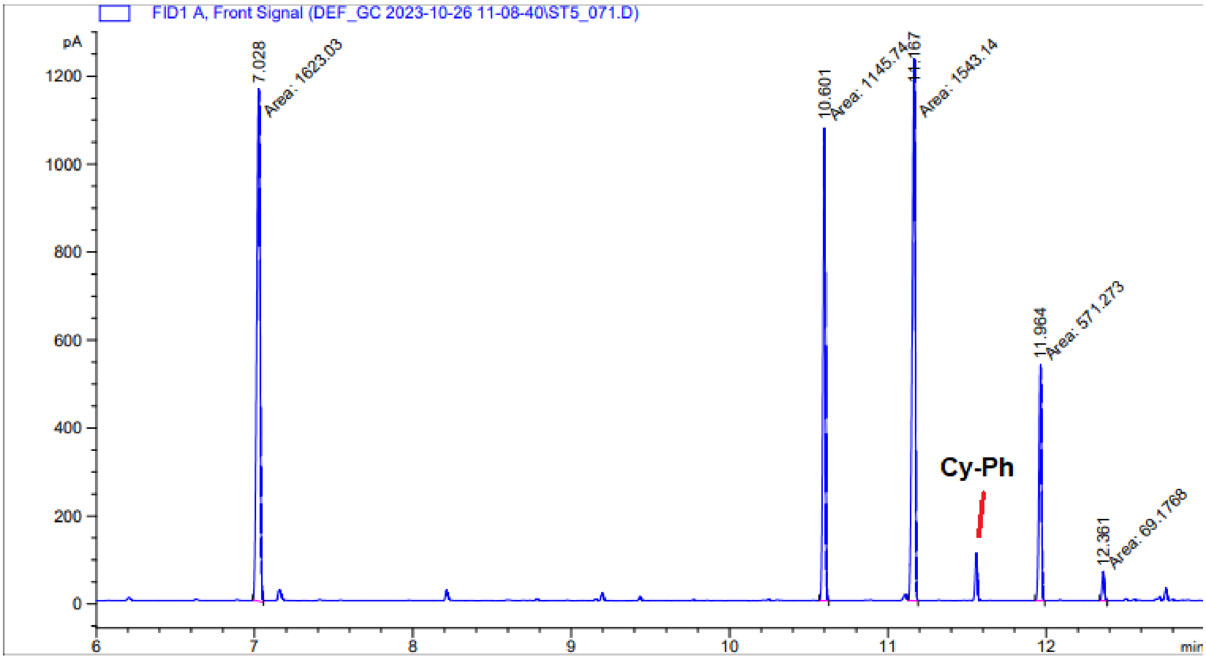

Entry 4 –

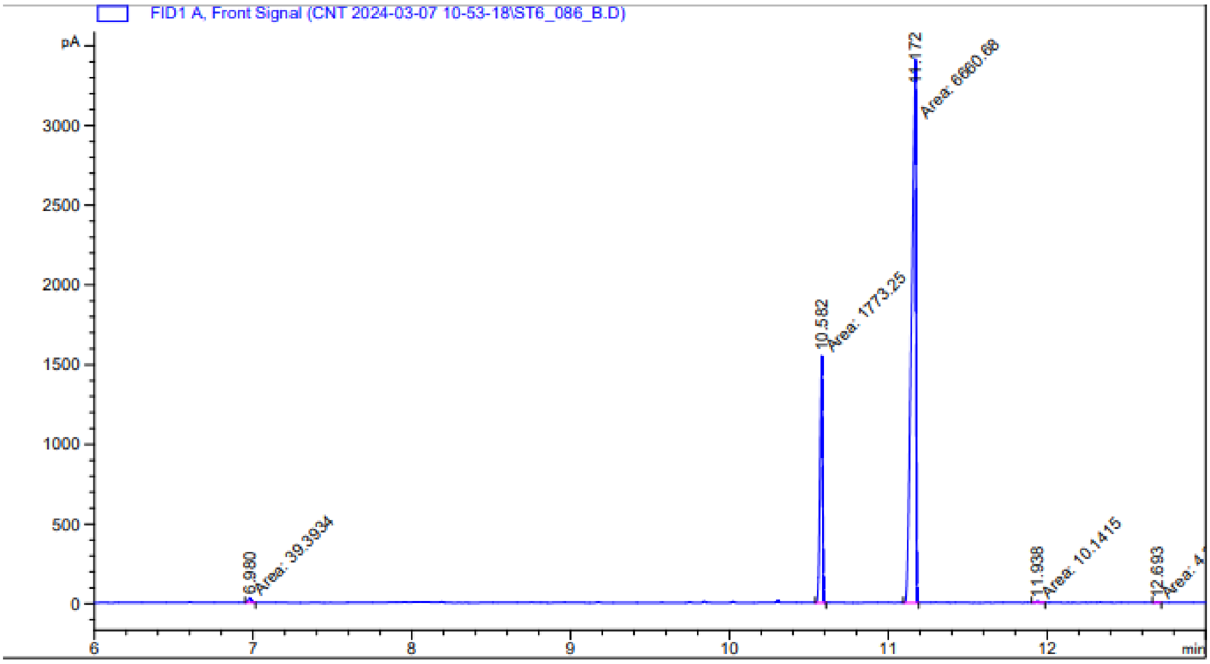

Entry 5 –

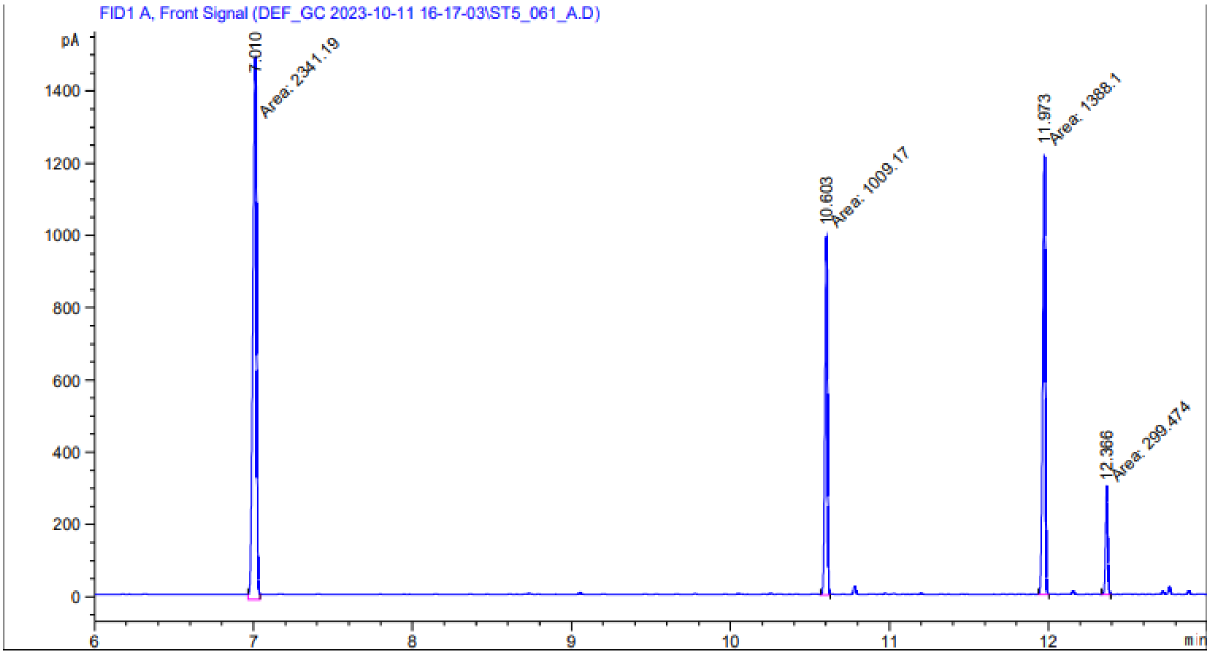

Entry 6 –

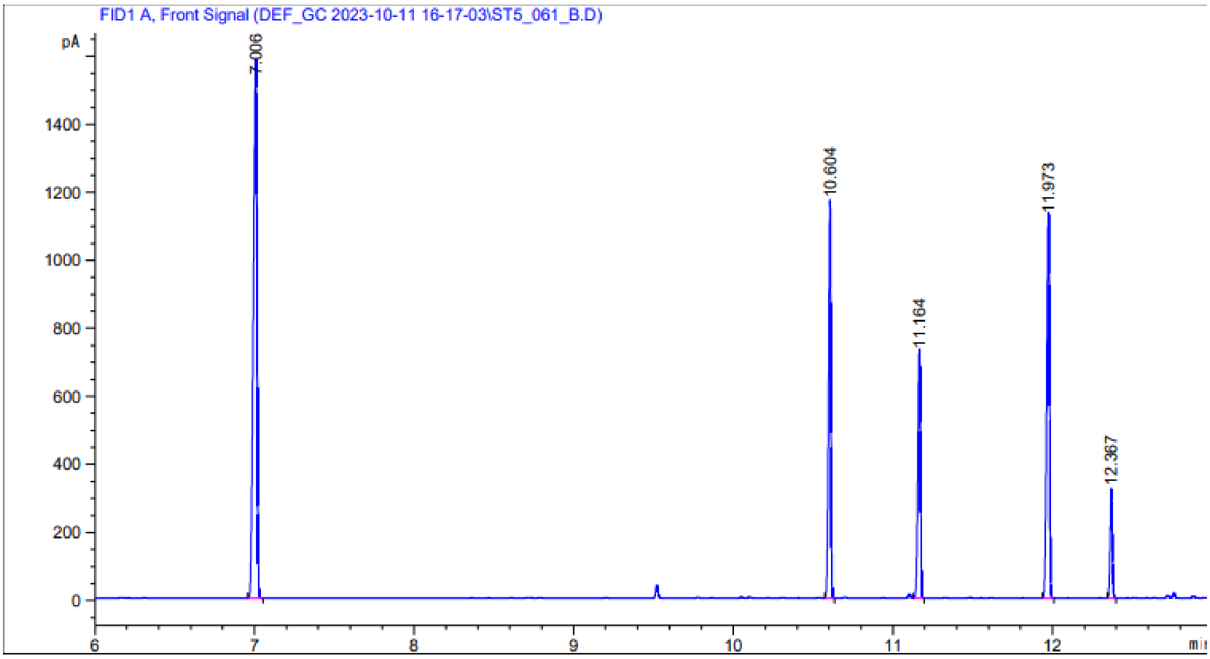

Entry 7 –

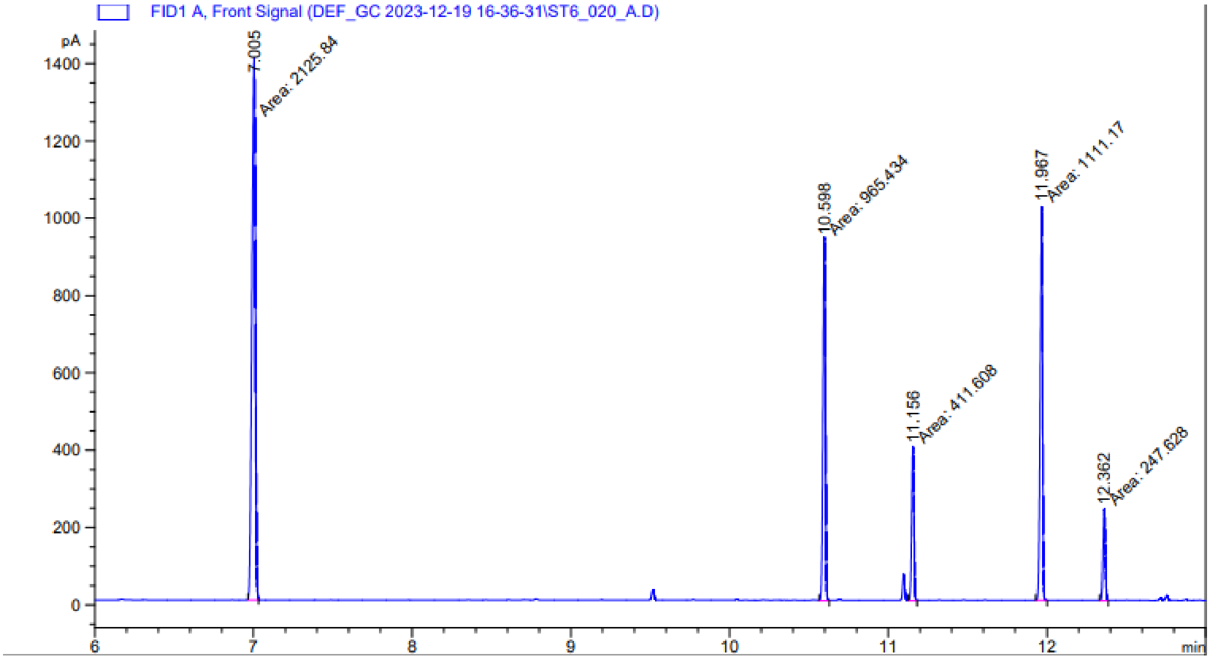

Entry 8 –

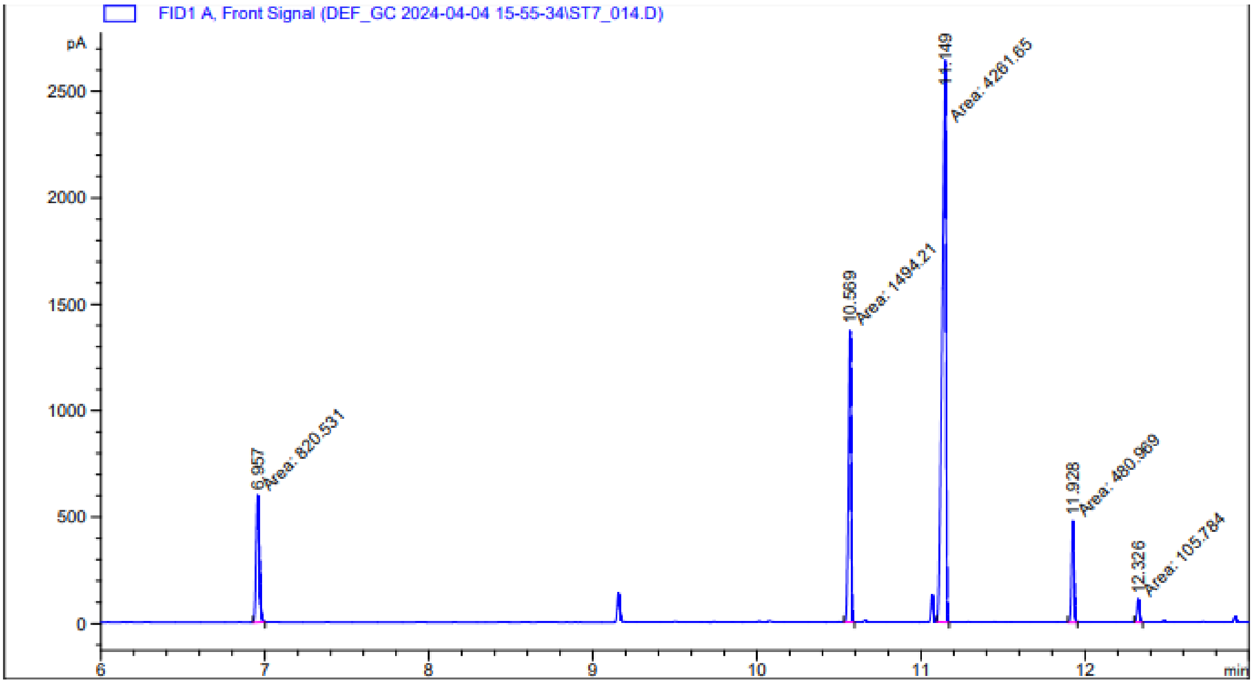

Entry 9 –

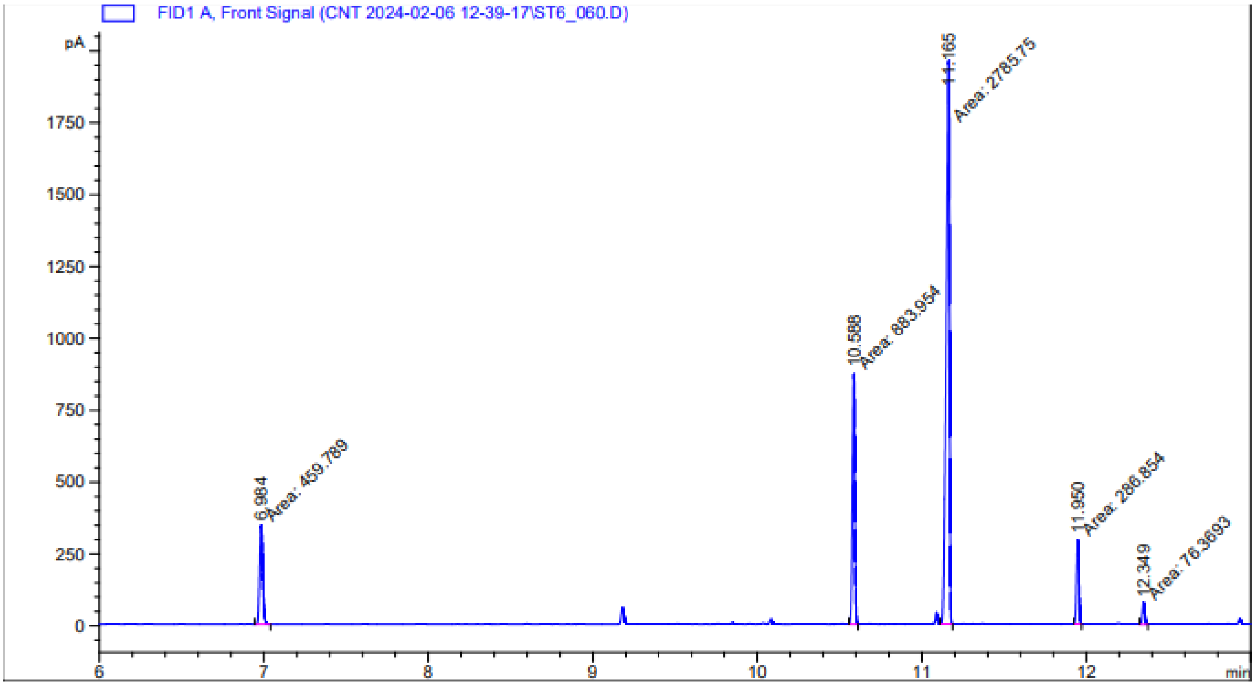

Entry 10 –

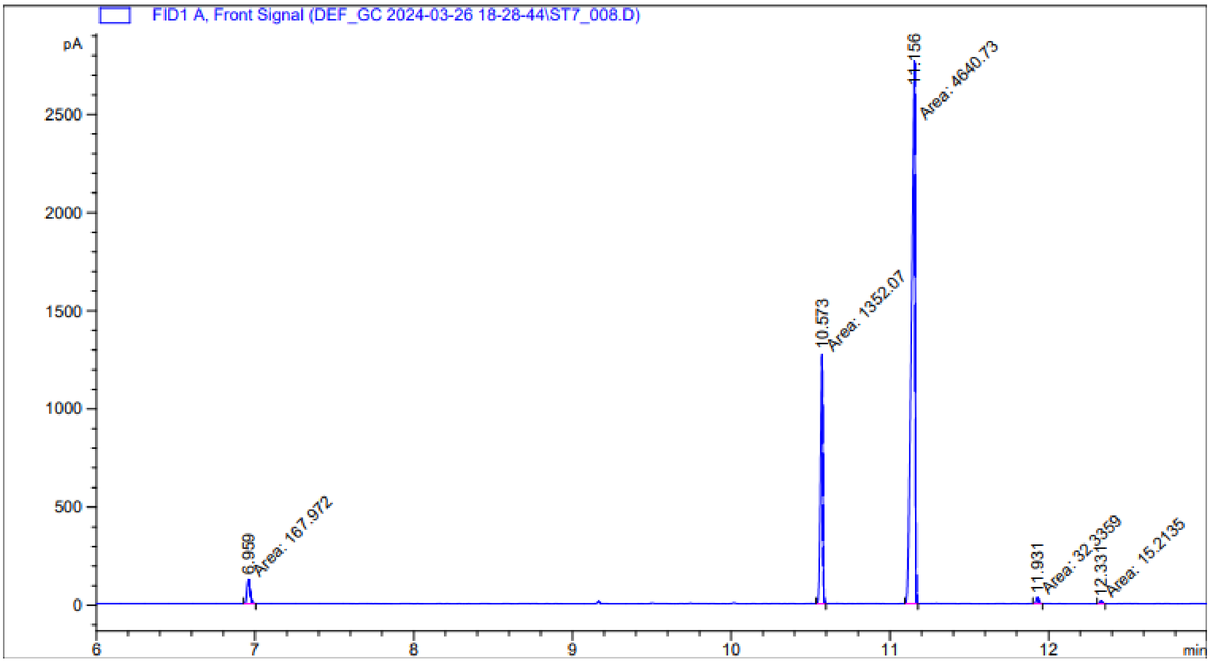

Entry 11 –

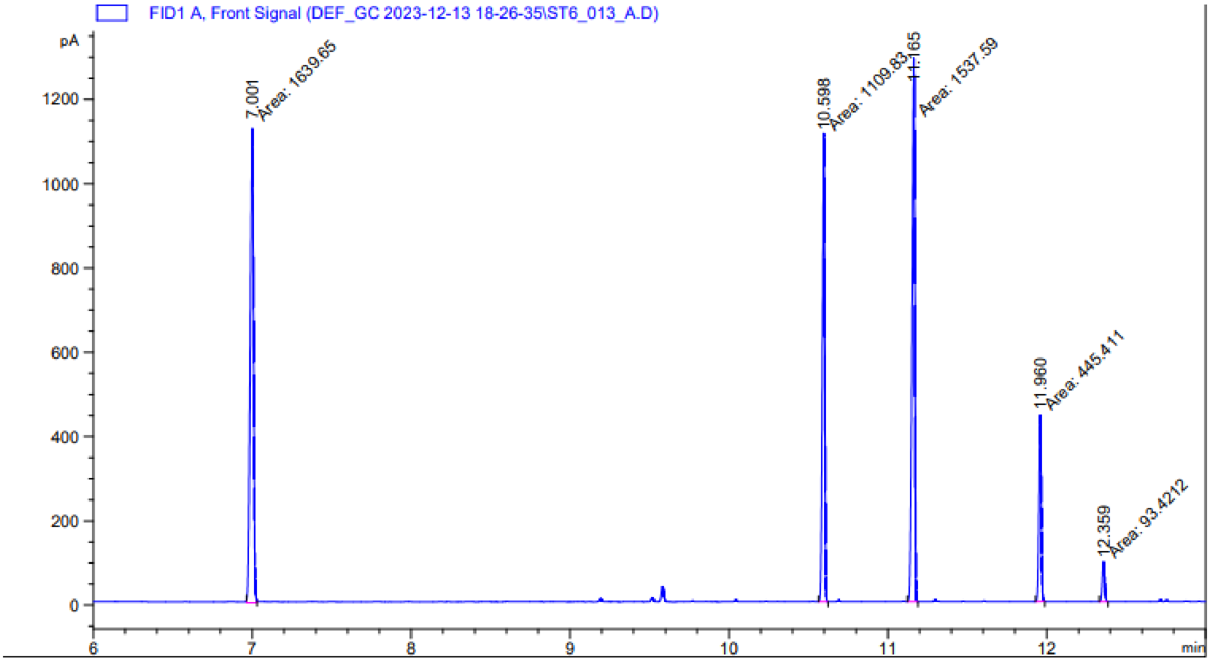

Entry 12 –

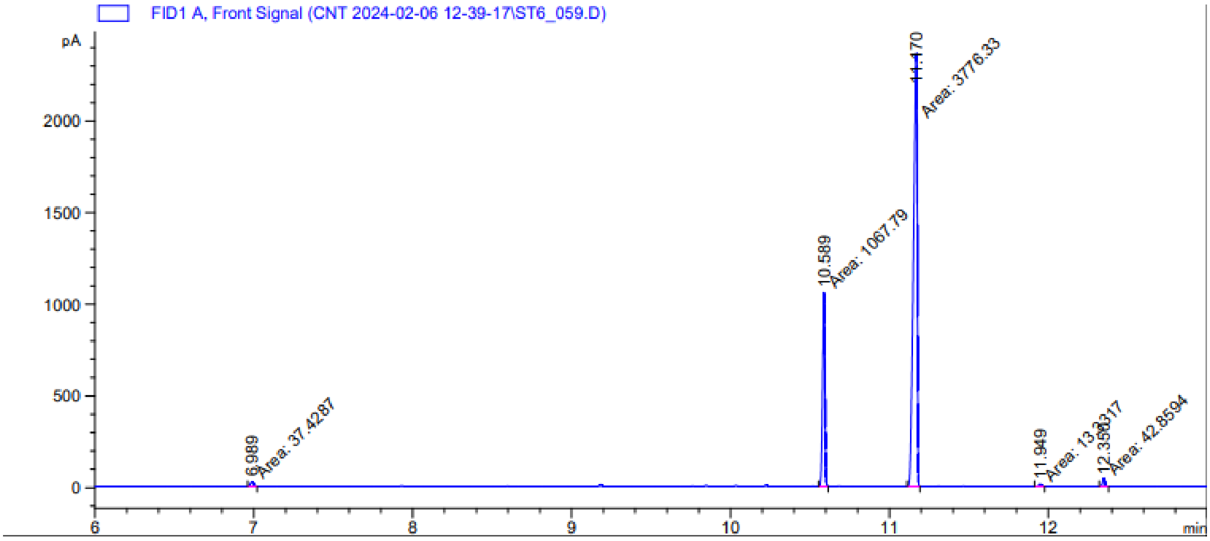

Entry 13 –

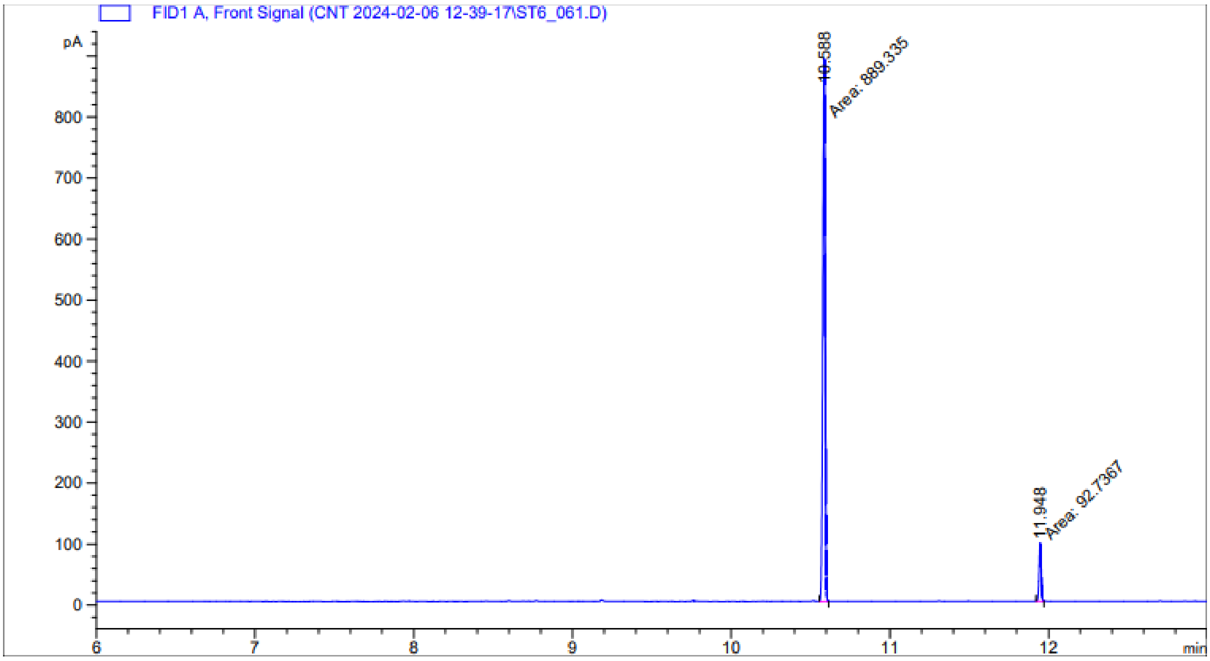

Entry 14 –

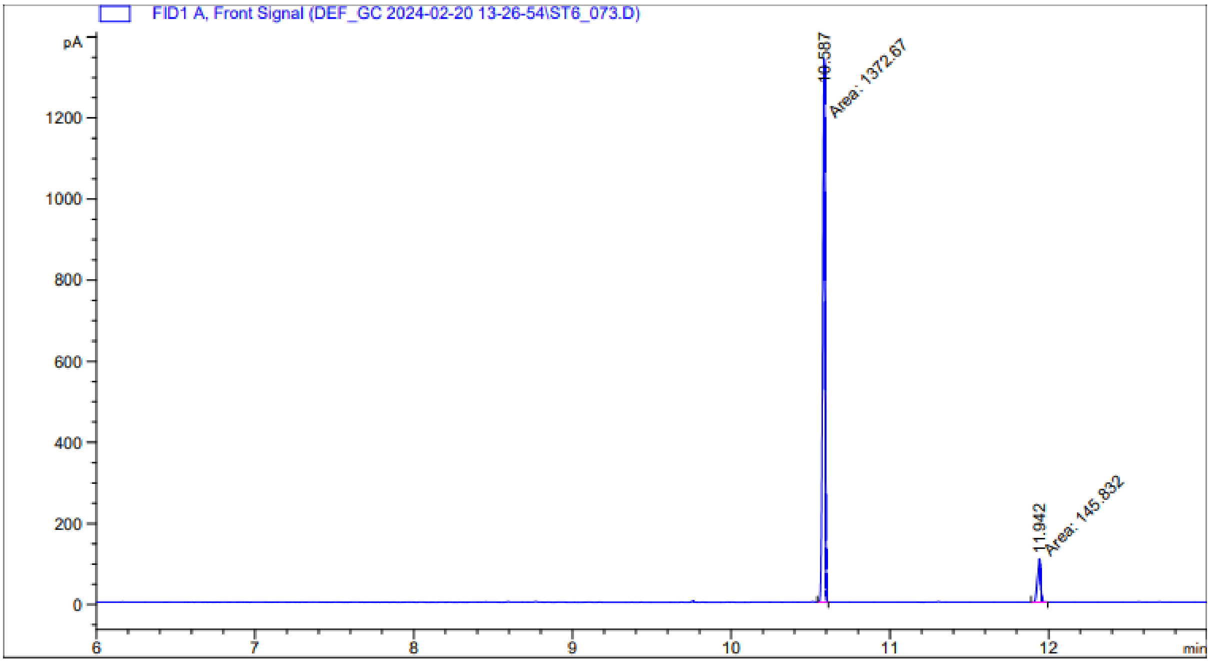

Entry 15 –

GC-FID traces for Table S2

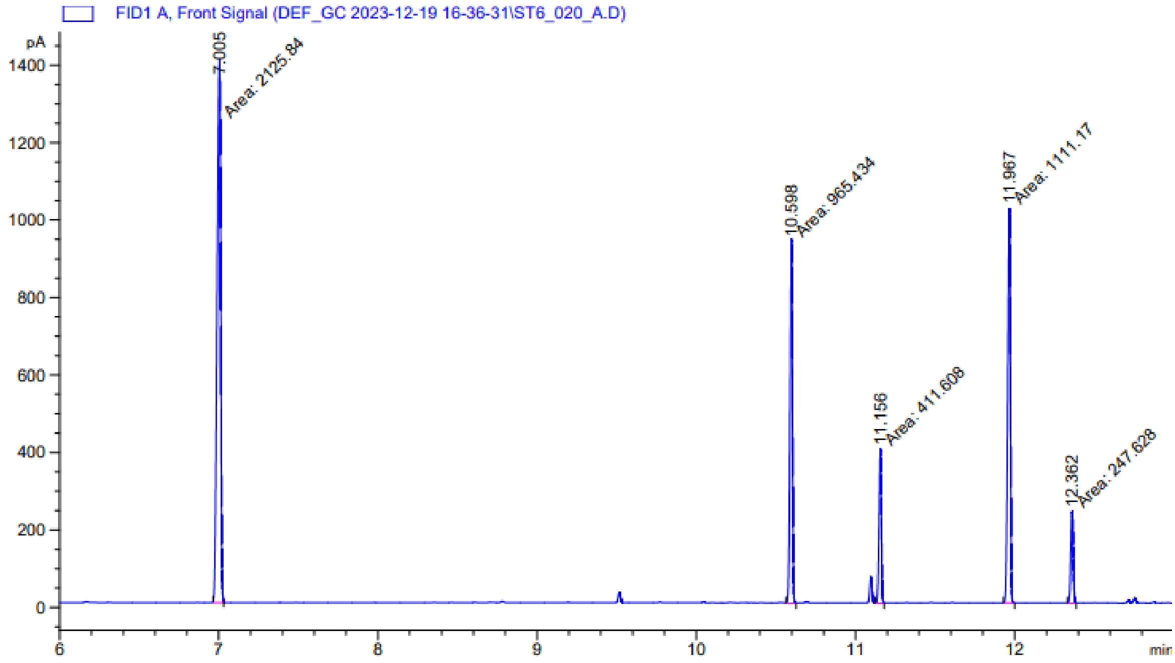

Entry 1

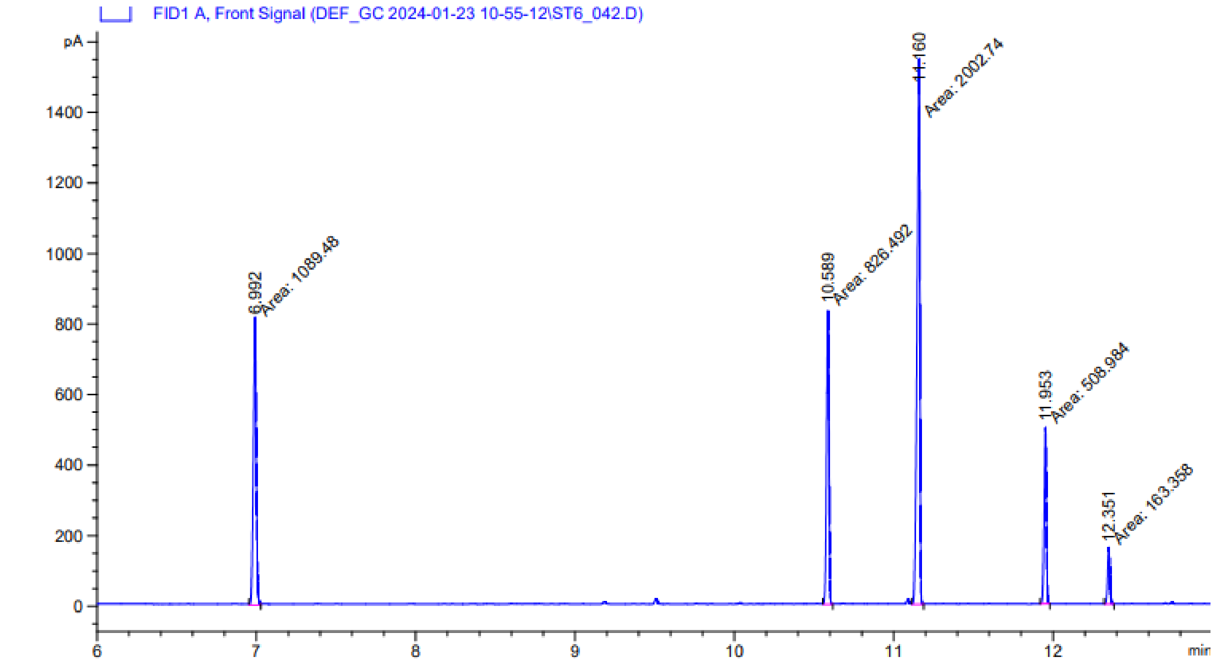

Entry 2

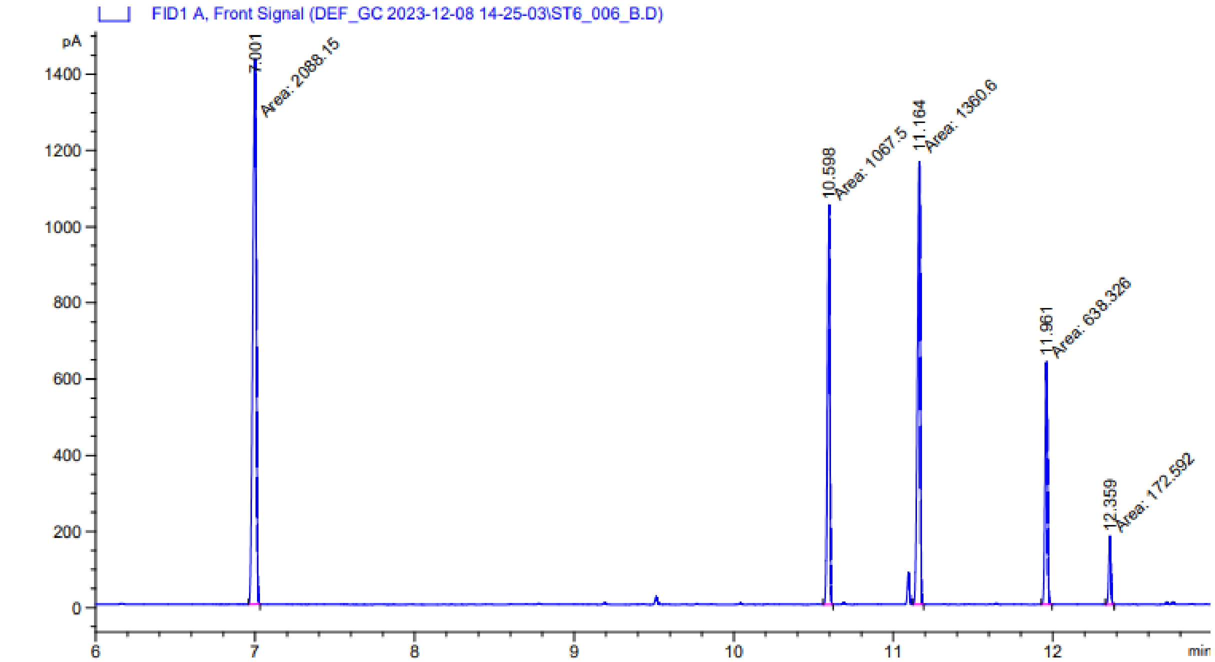

Entry 3

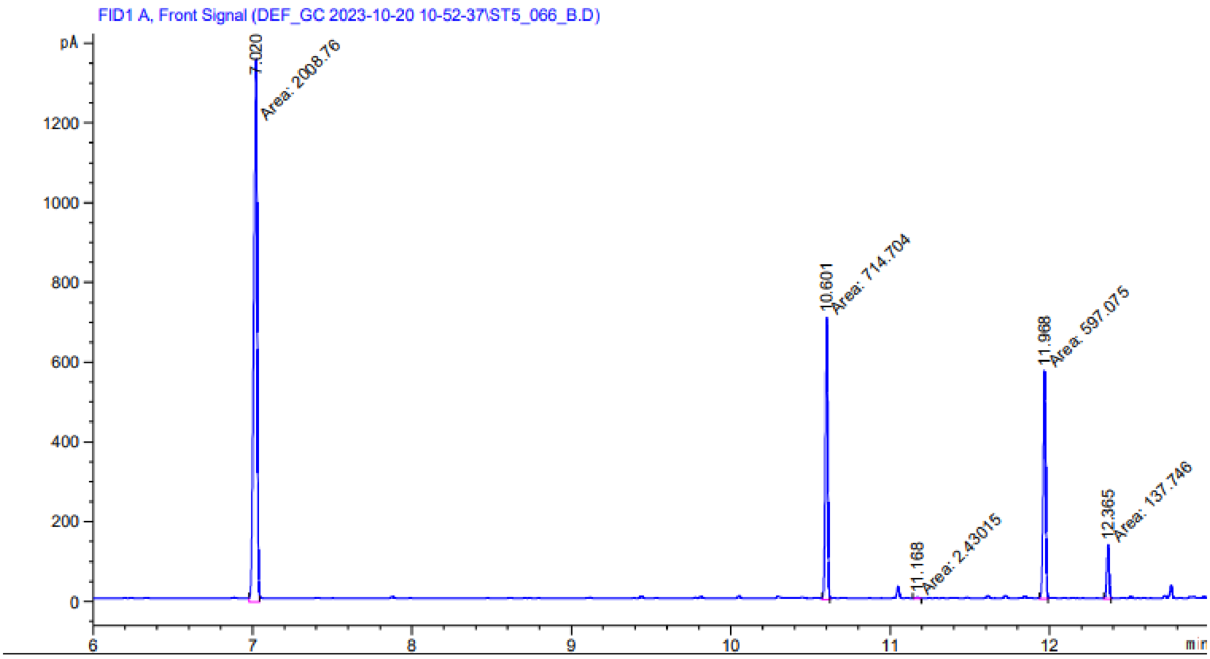

Entry 4

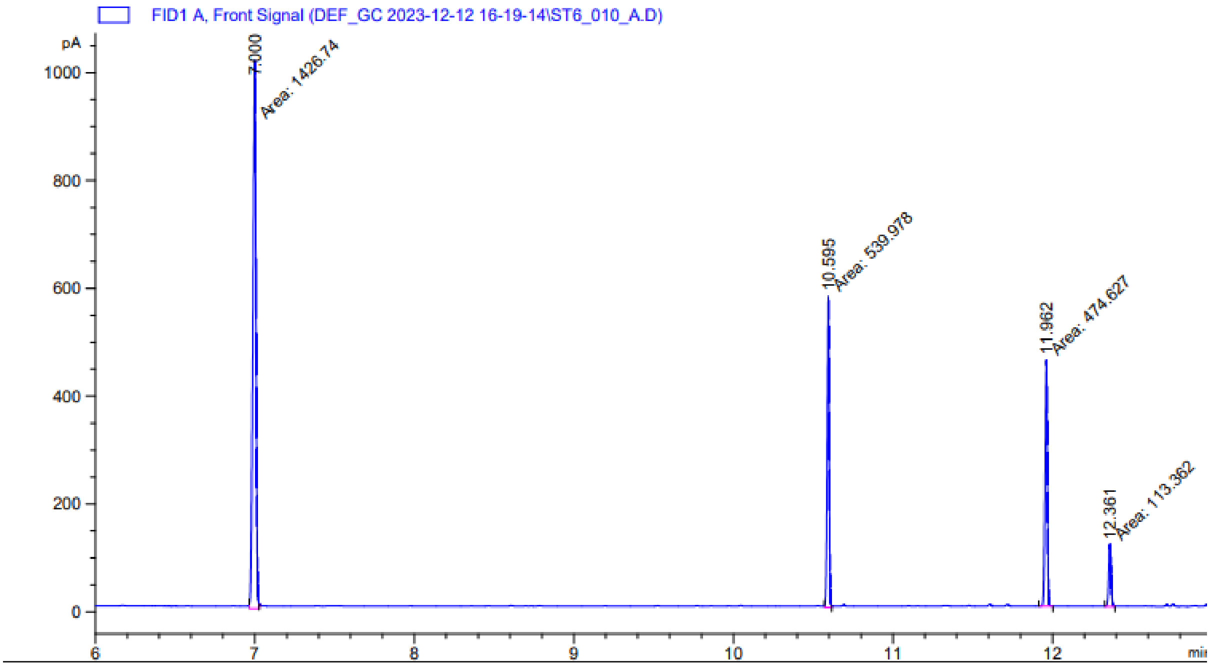

Entry 5

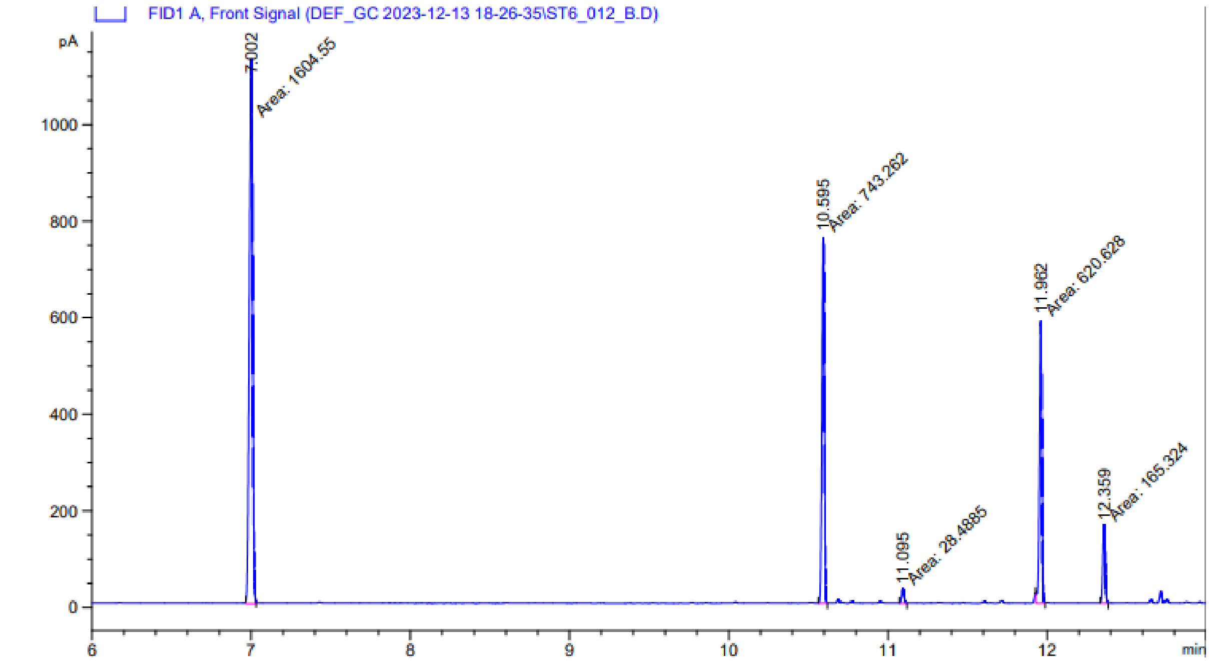

Entry 6

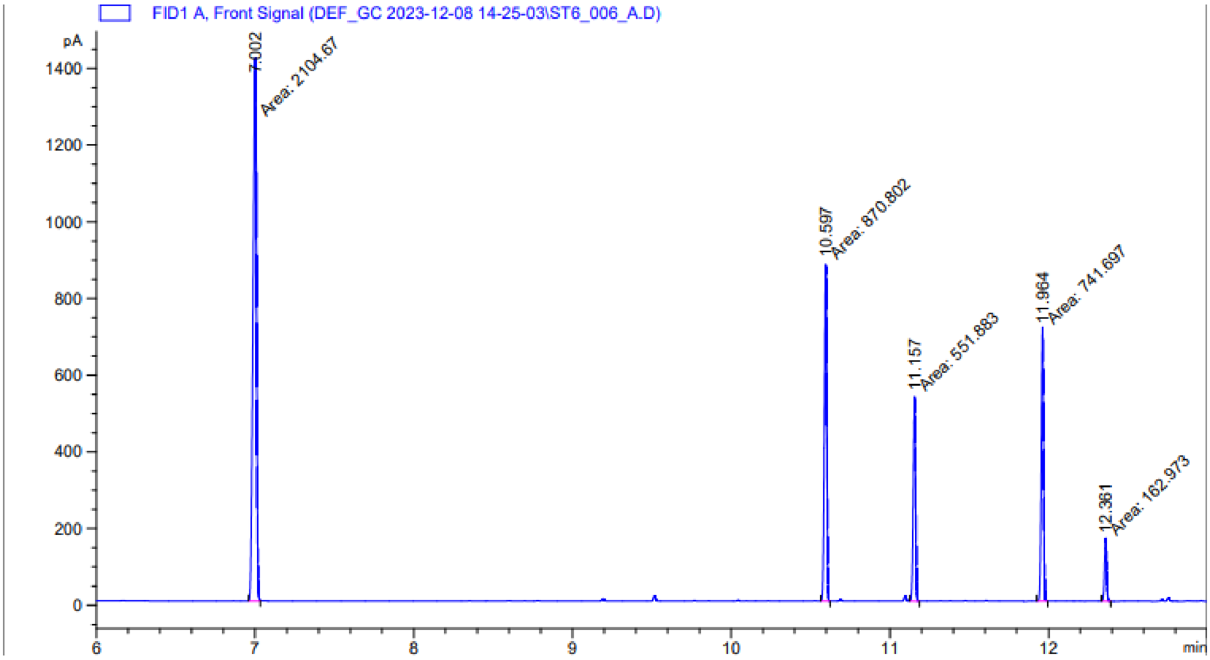

Entry 7

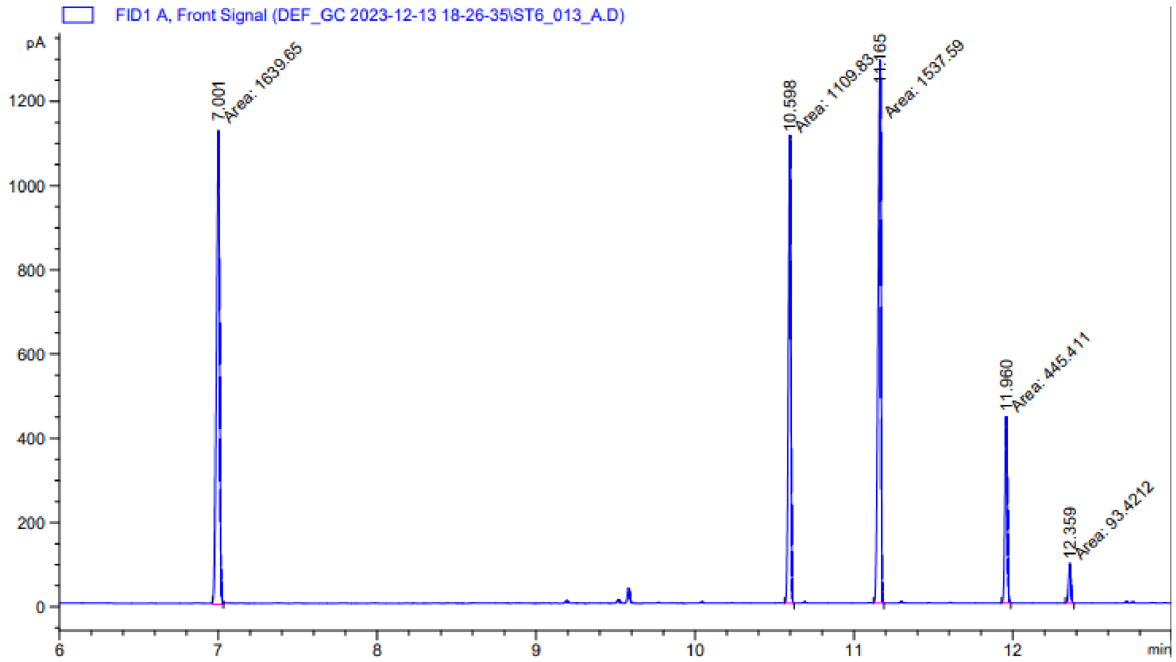

Entry 8

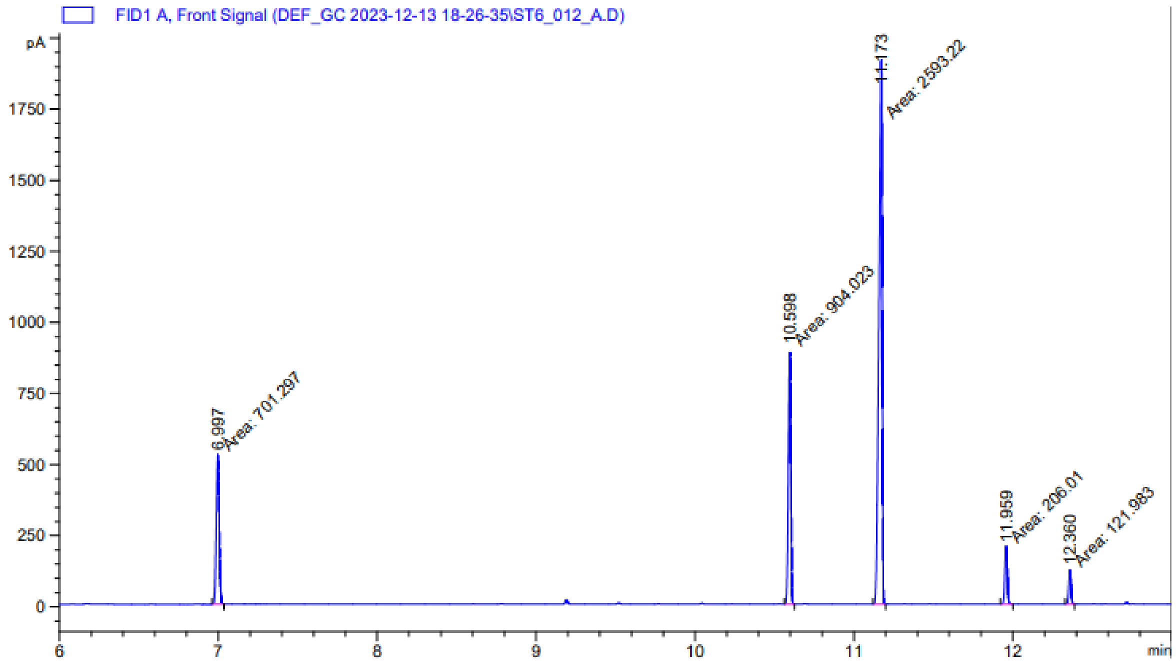

Entry 9

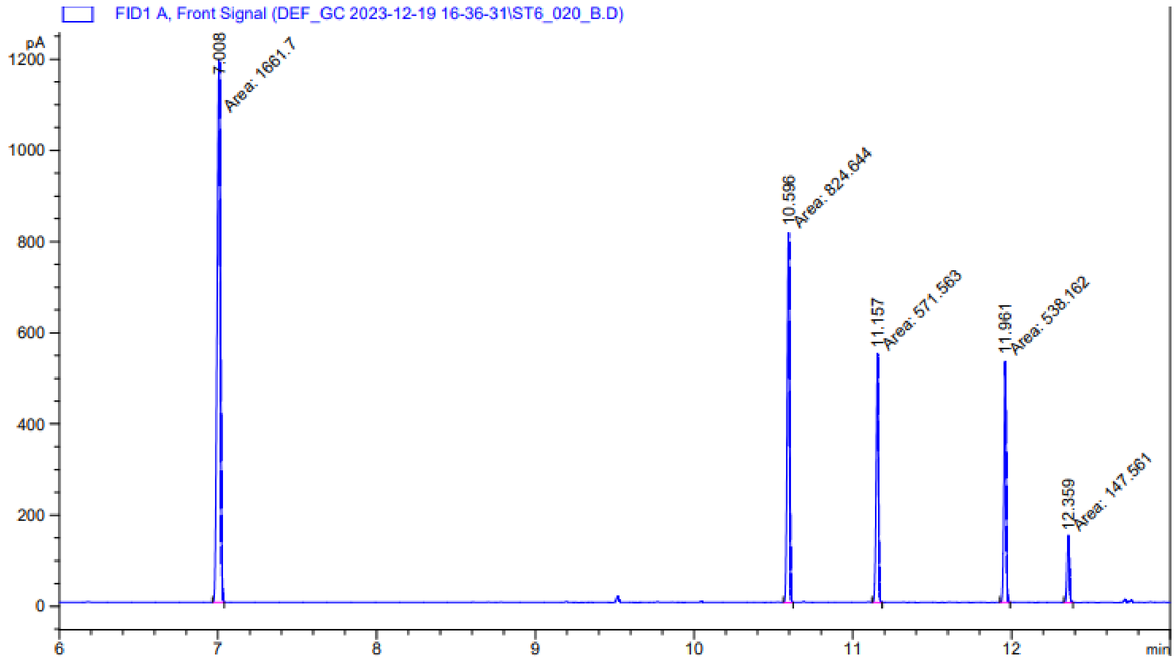

Entry 10

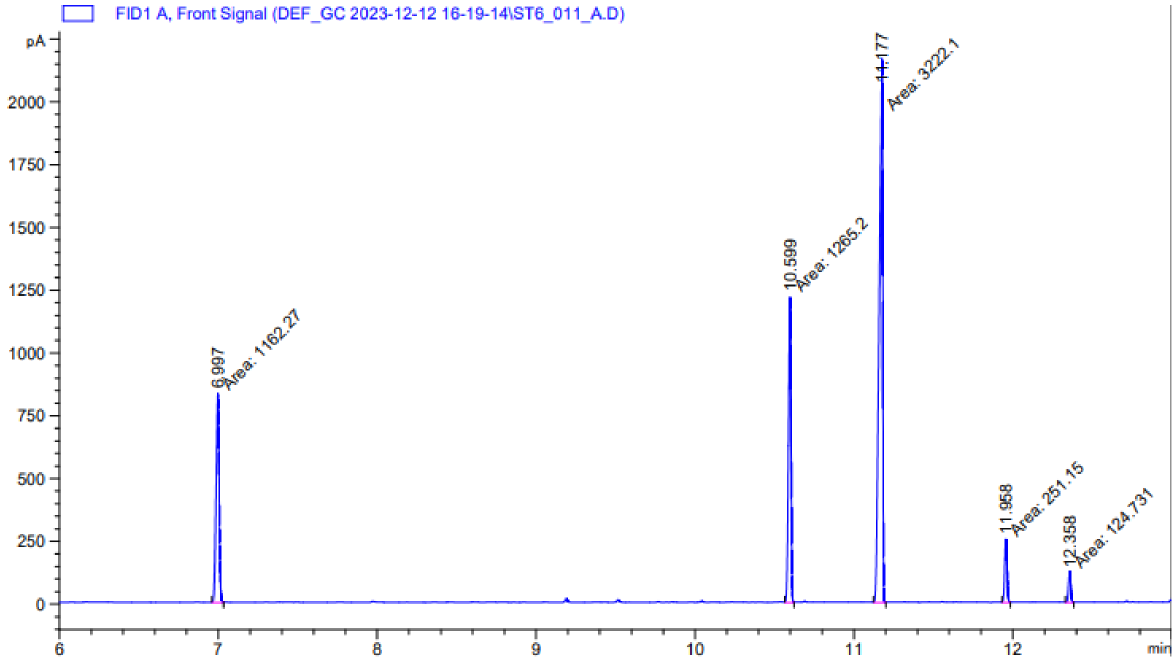

Entry 11

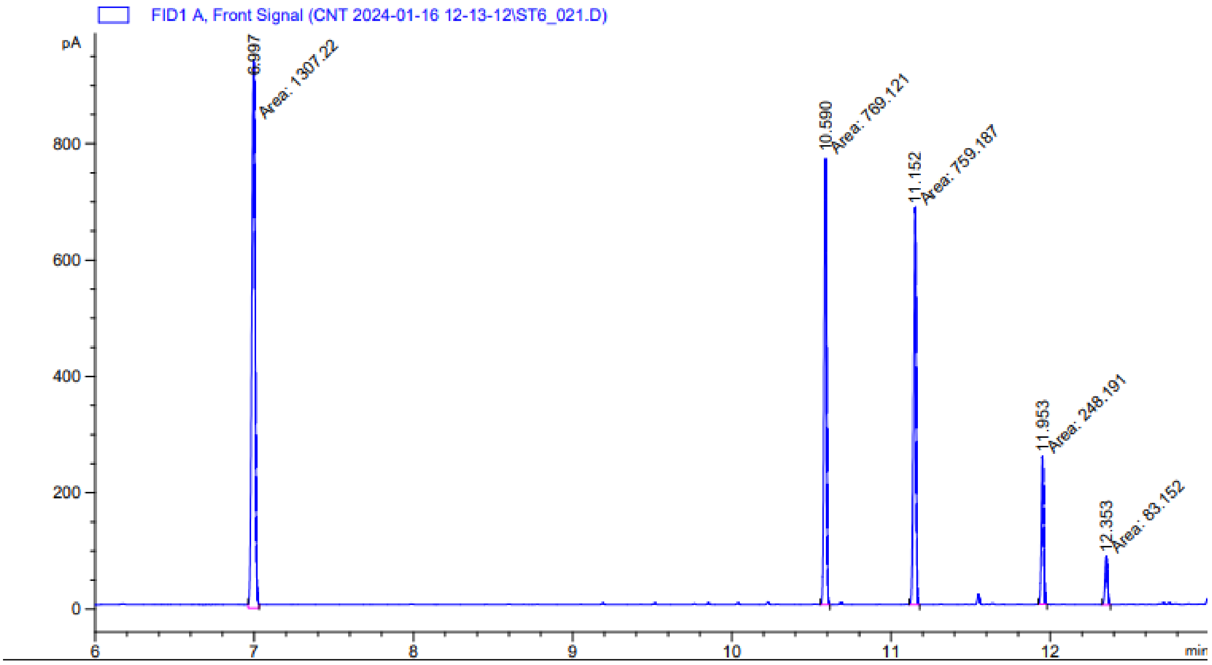

Entry 12

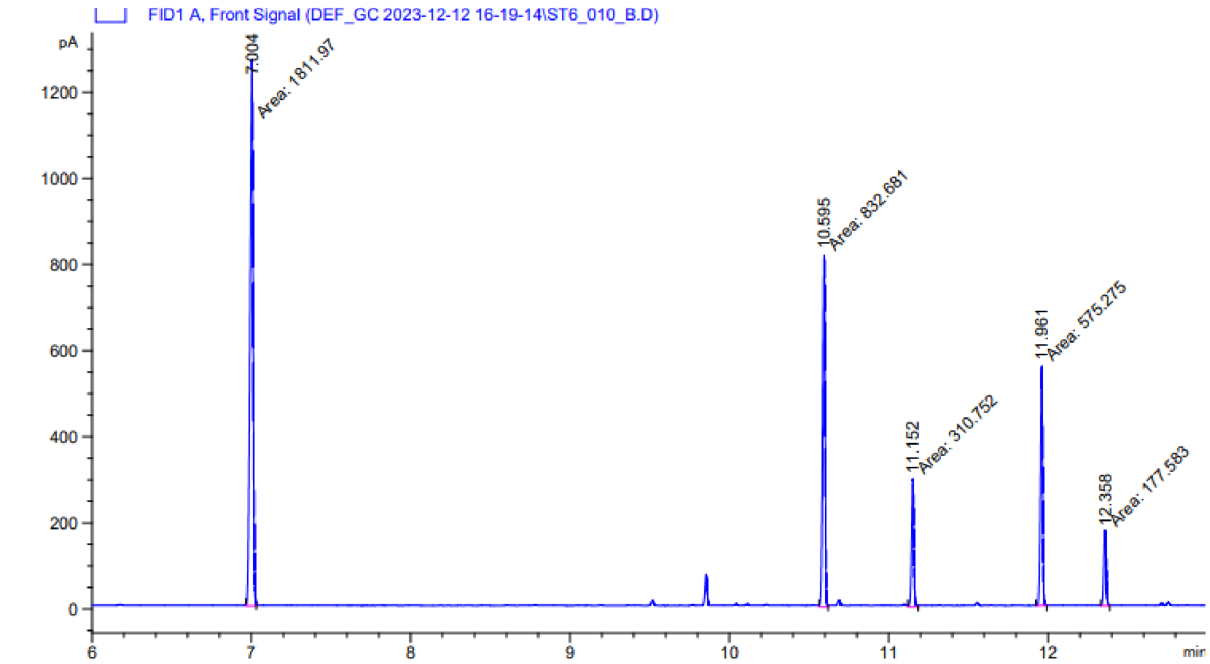

Entry 13

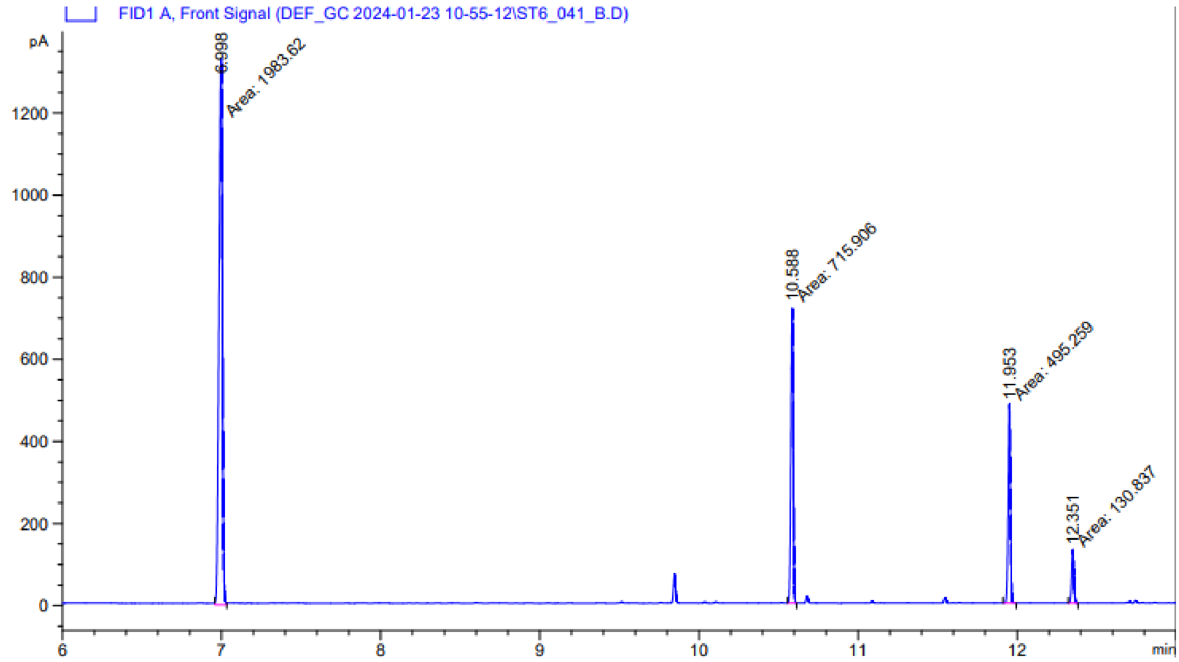

Entry 14

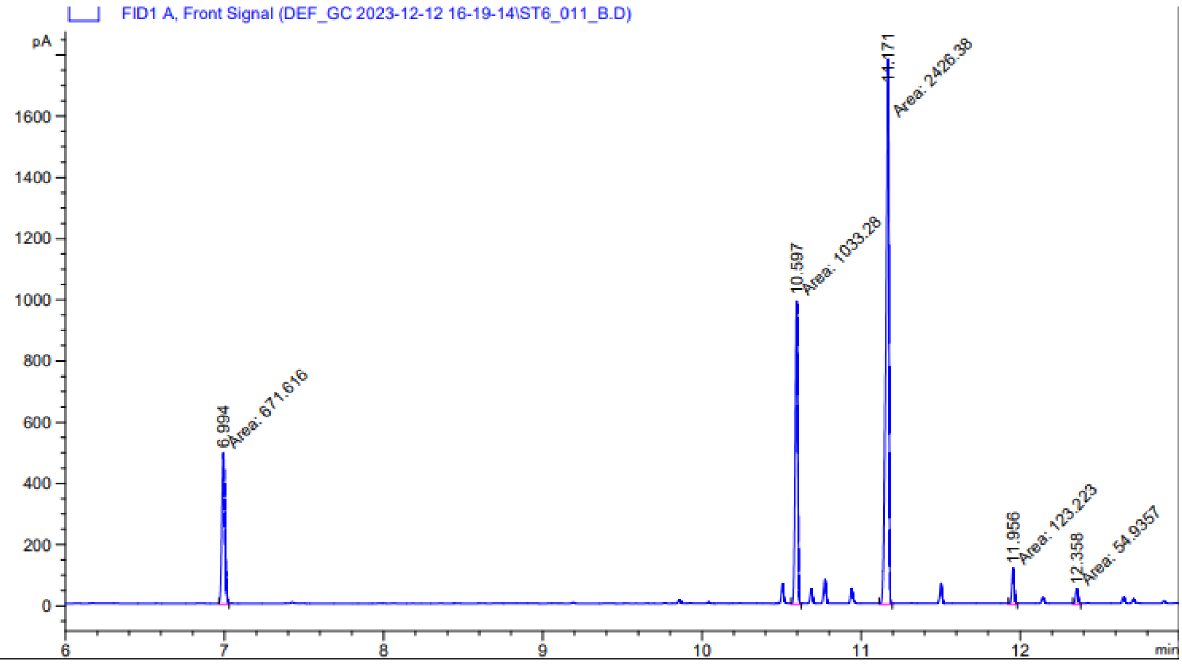

Entry 15

GC-FID traces for Table S3

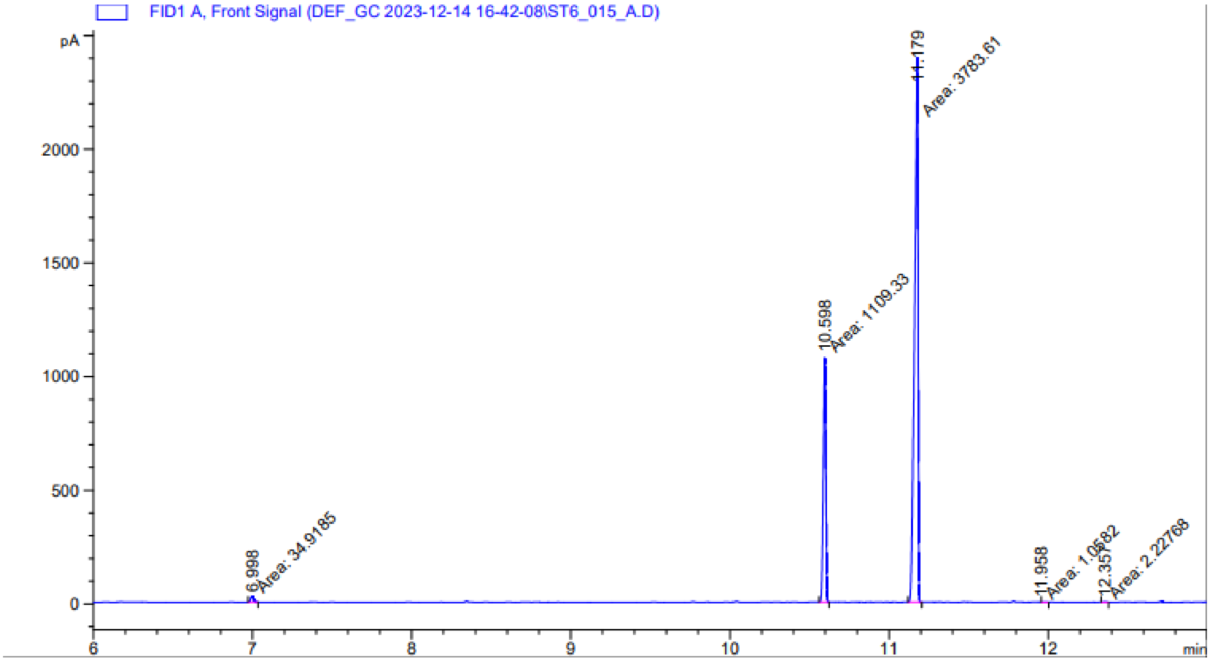

Entry 1

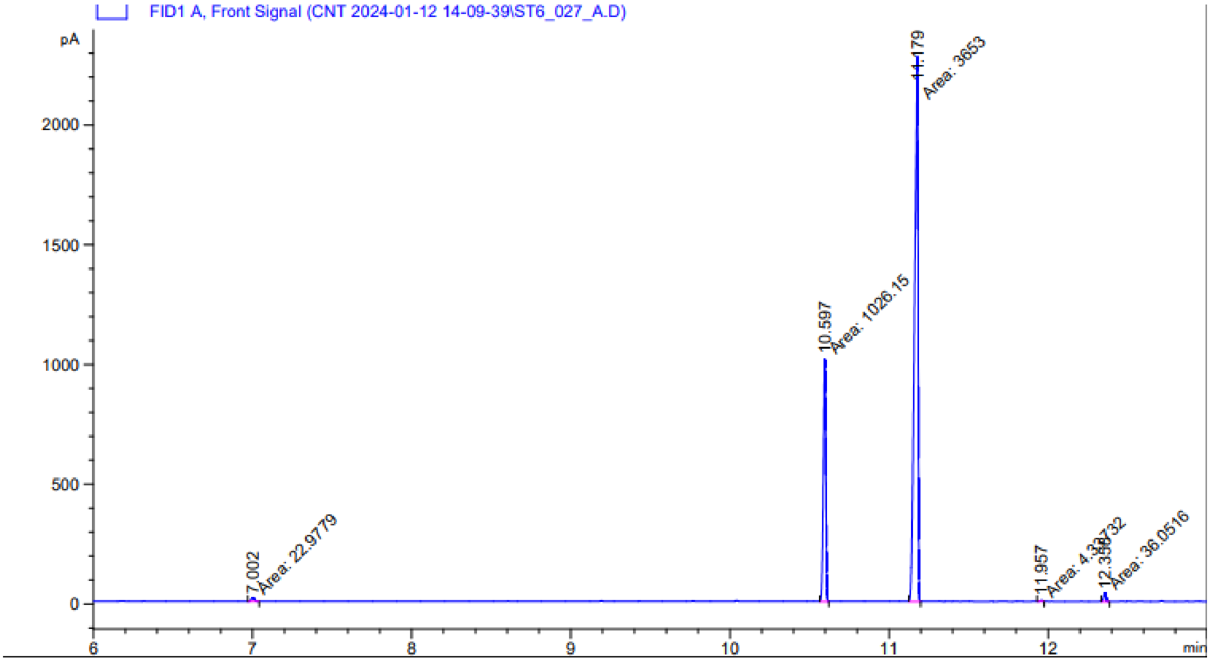

Entry 2

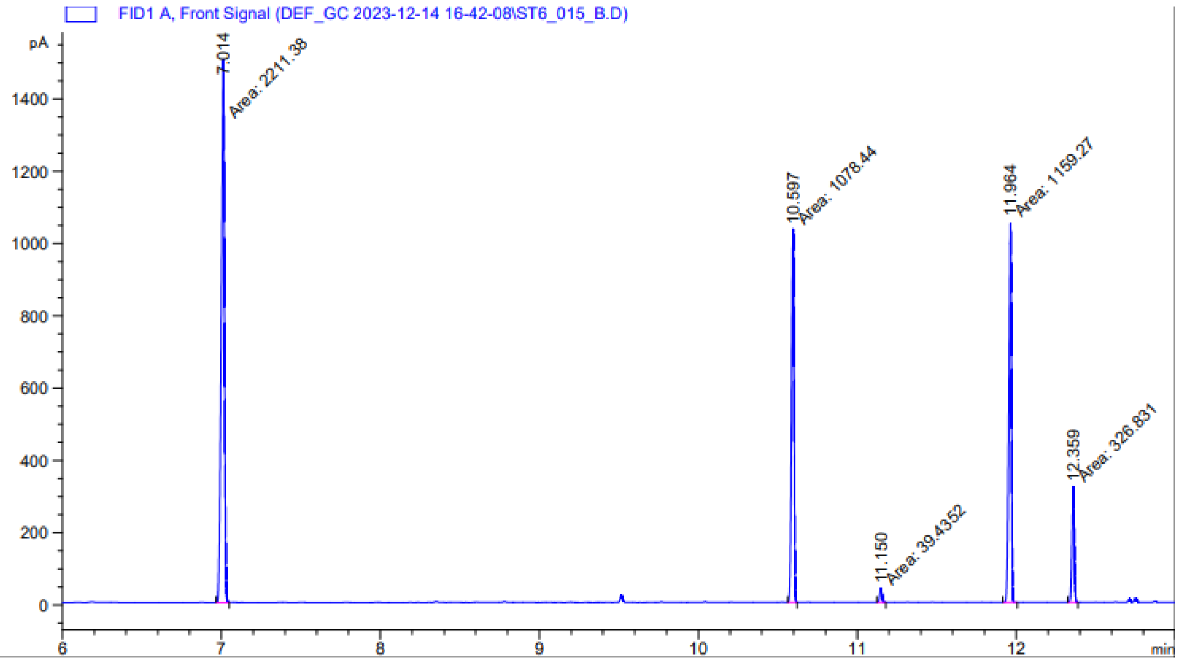

Entry 3

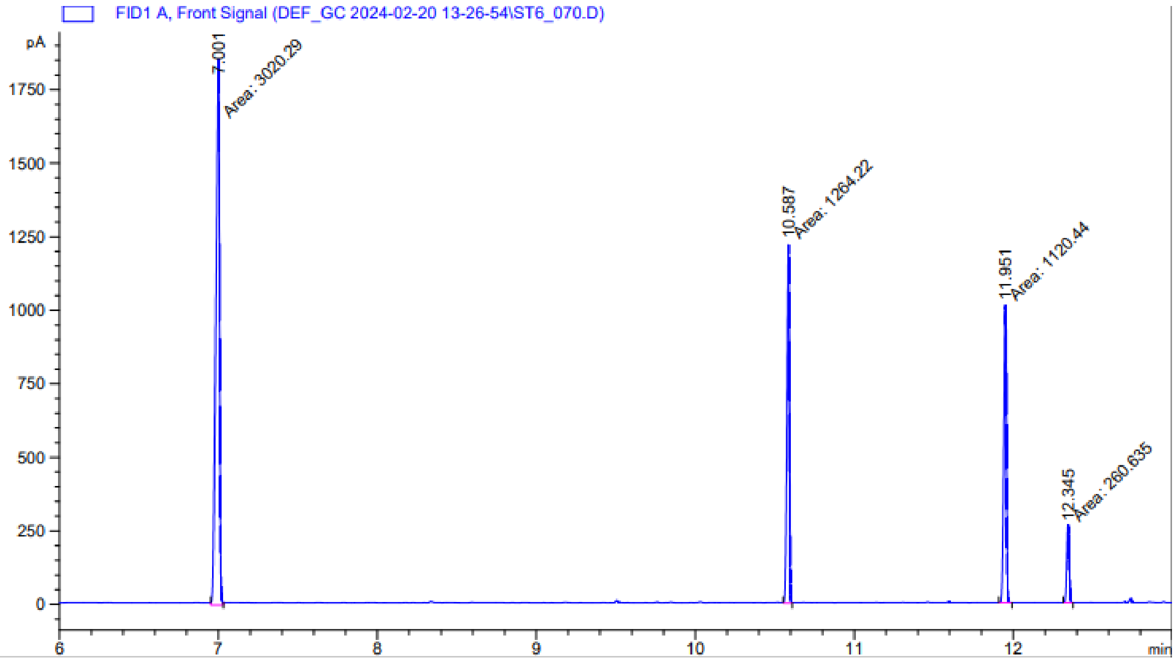

Entry 4

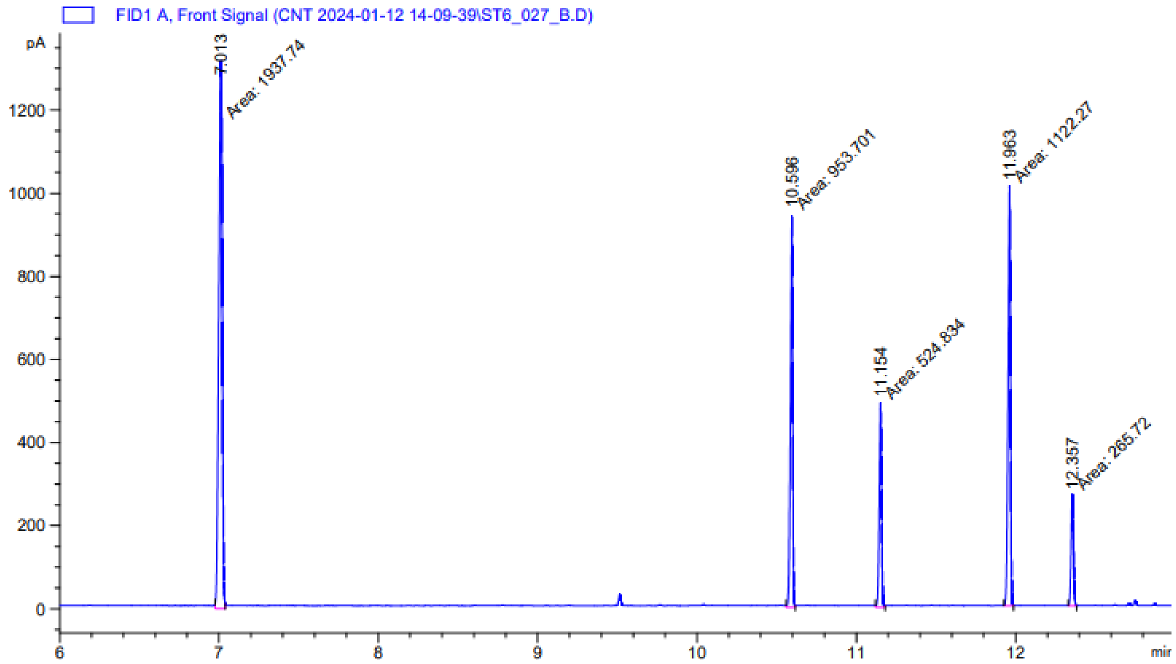

Entry 5

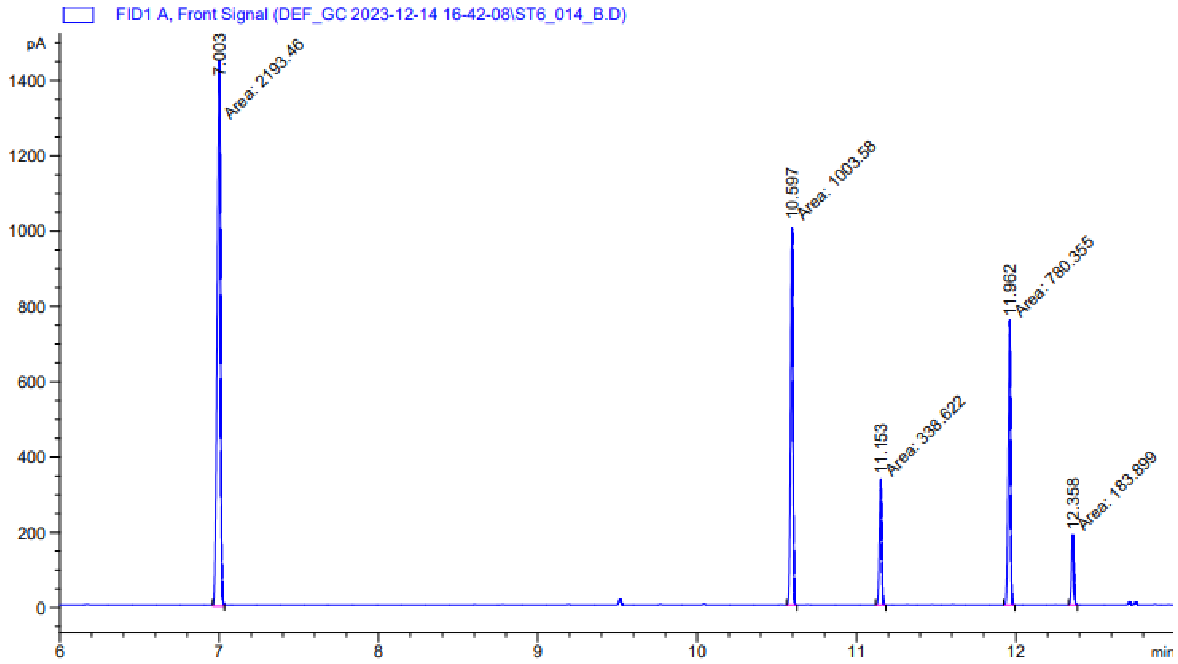

Entry 6

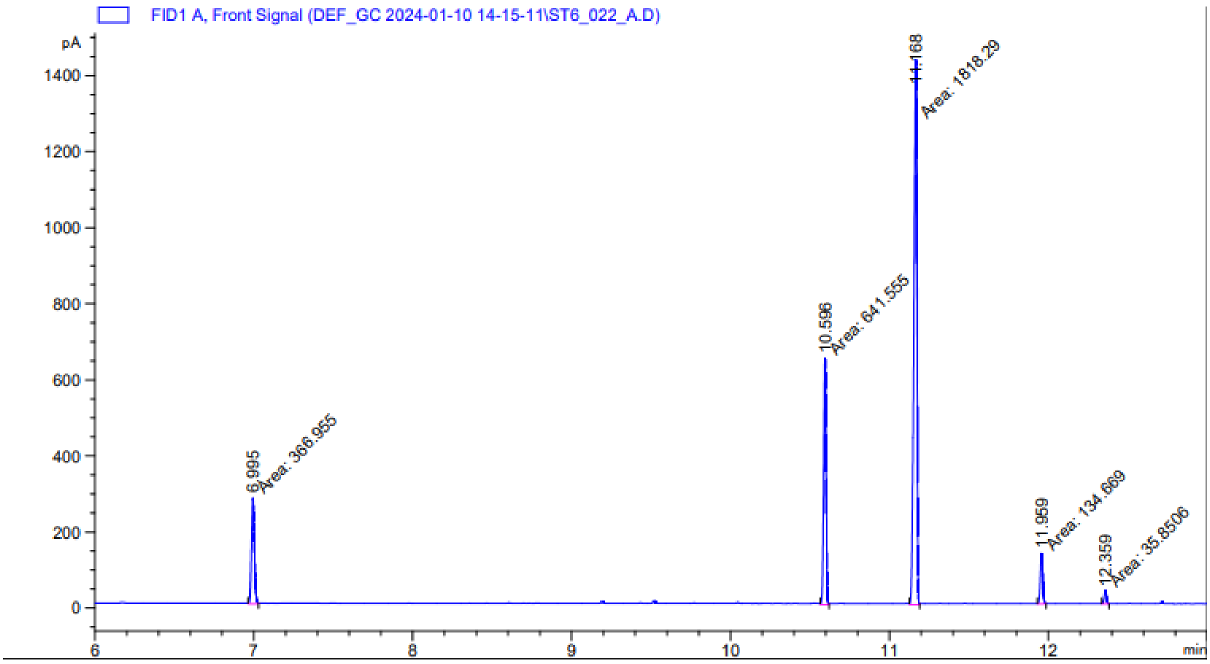

Entry 7

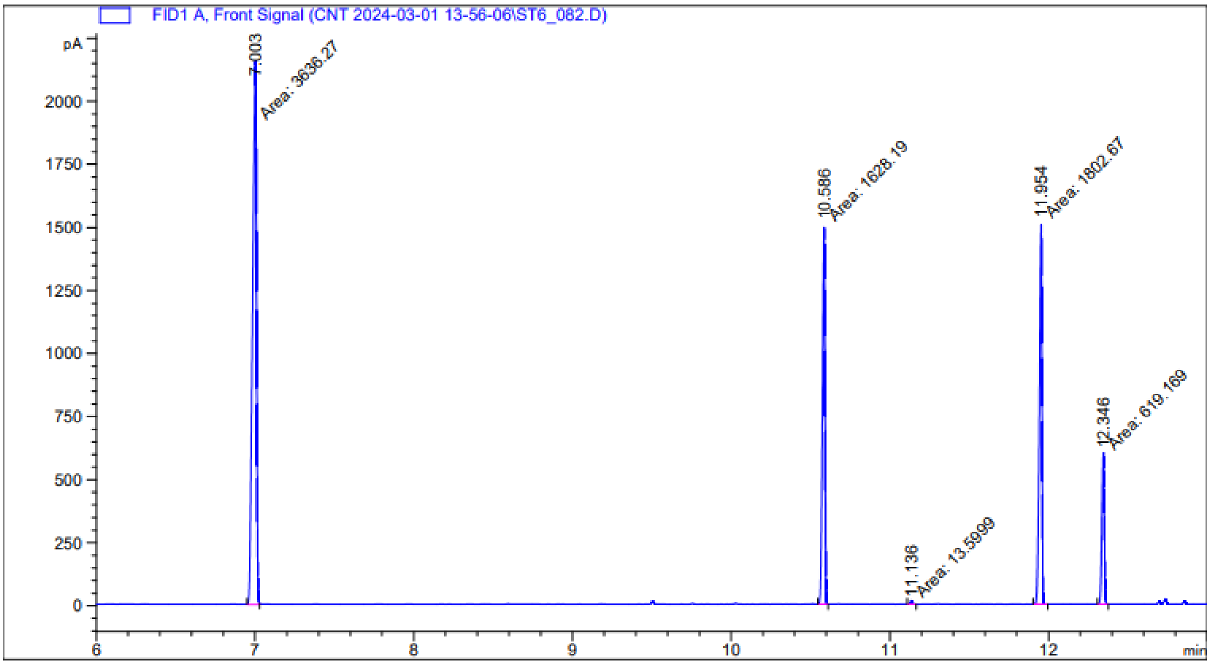

Entry 8

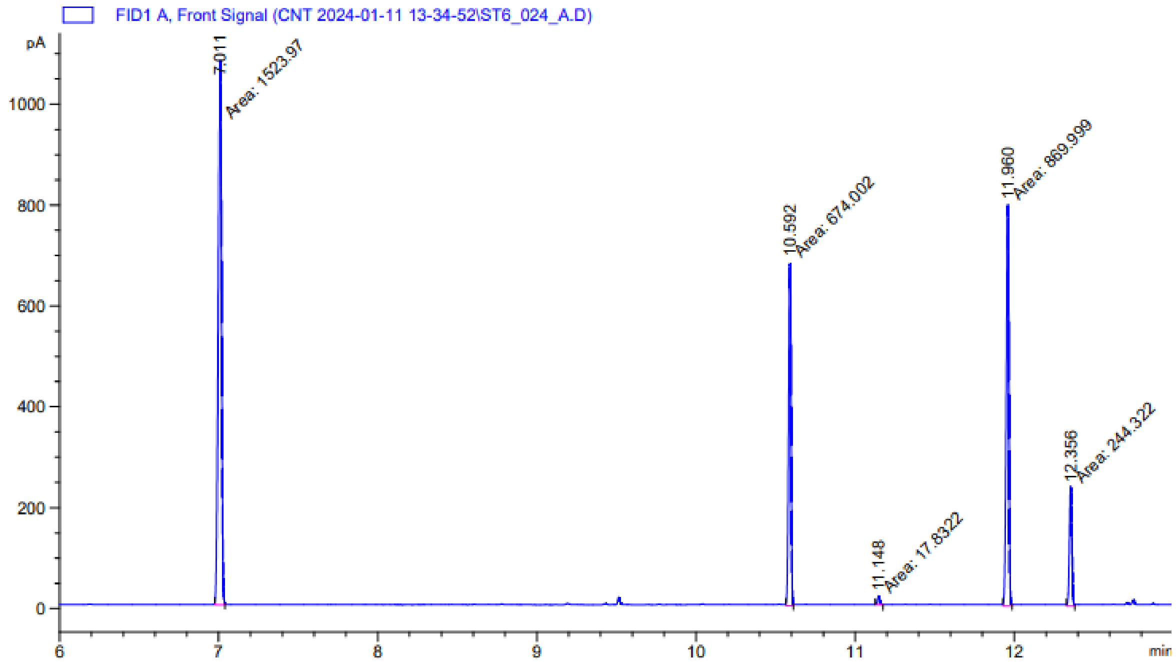

Entry 9

GC-FID traces for Table S4

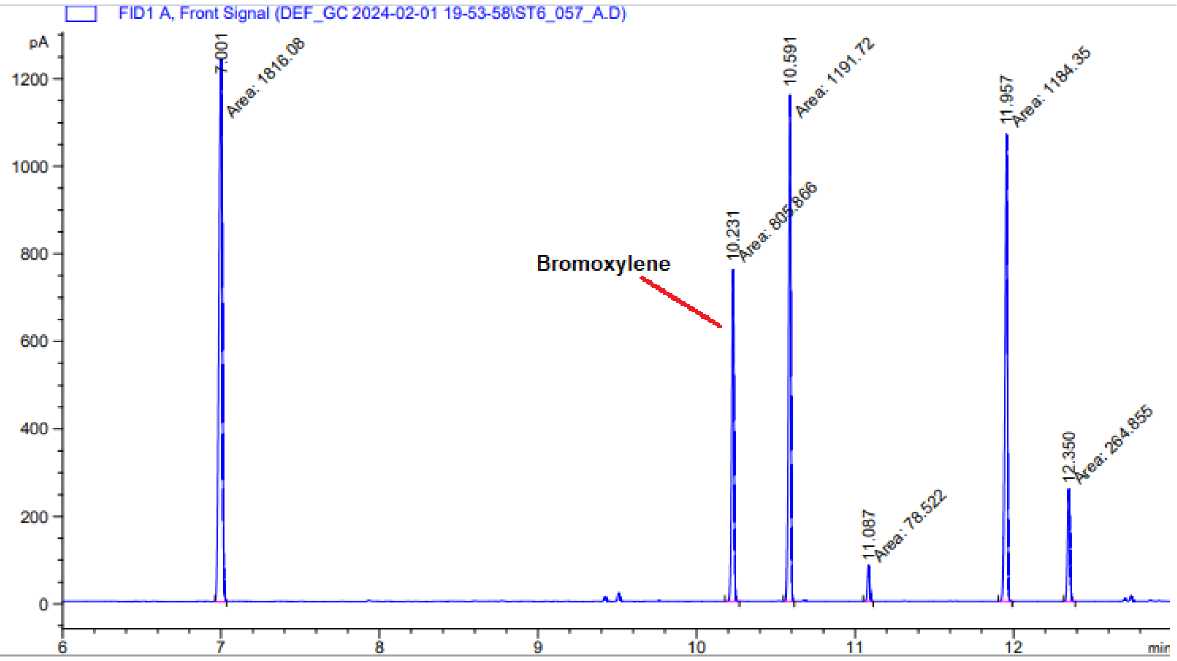

Entry 1 – Peak at 11.09 mins is Xylyl-OAc, not iodoxylene.

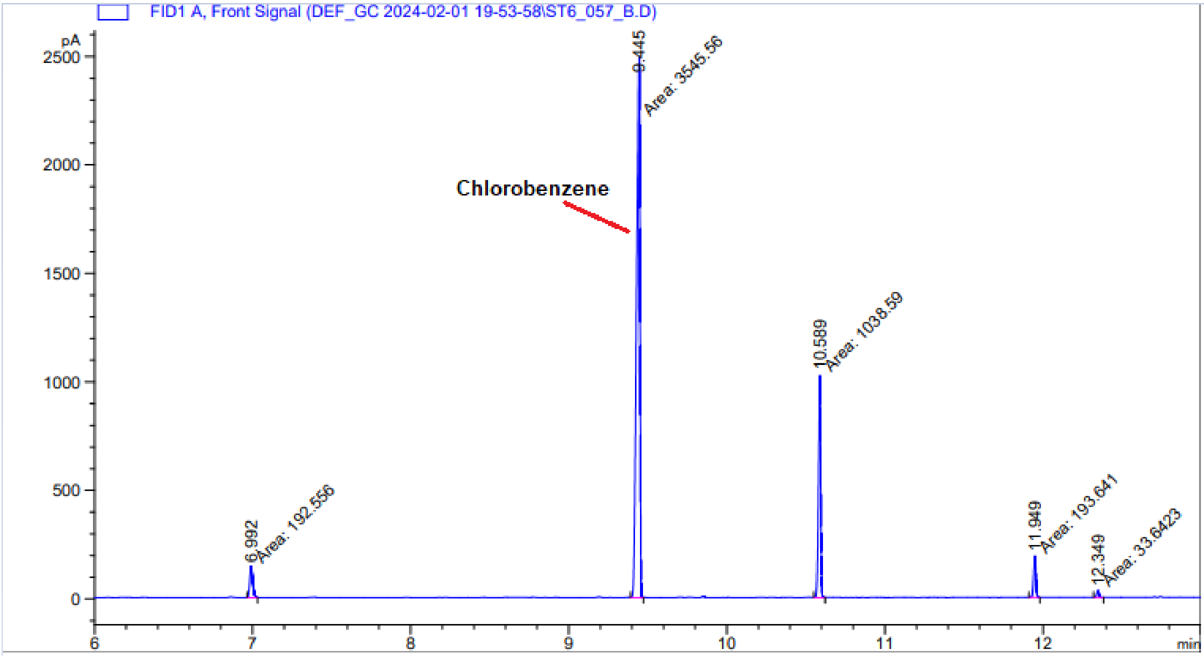

Entry 2

GC-FID traces for Table S5

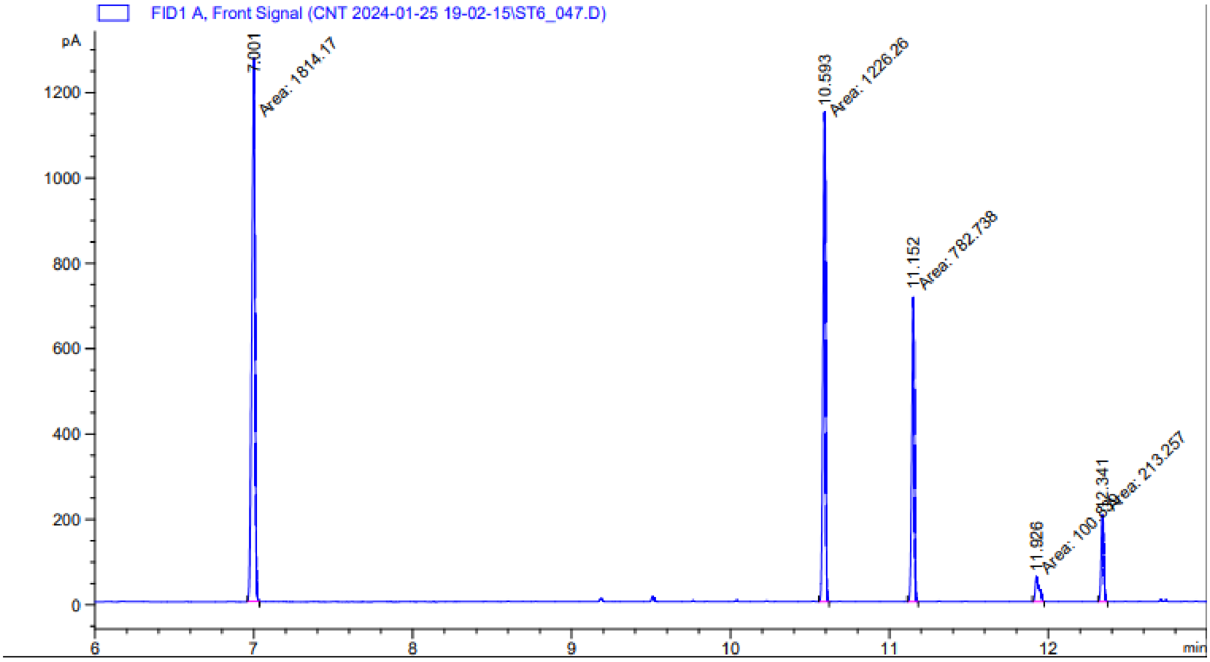

Entry 1

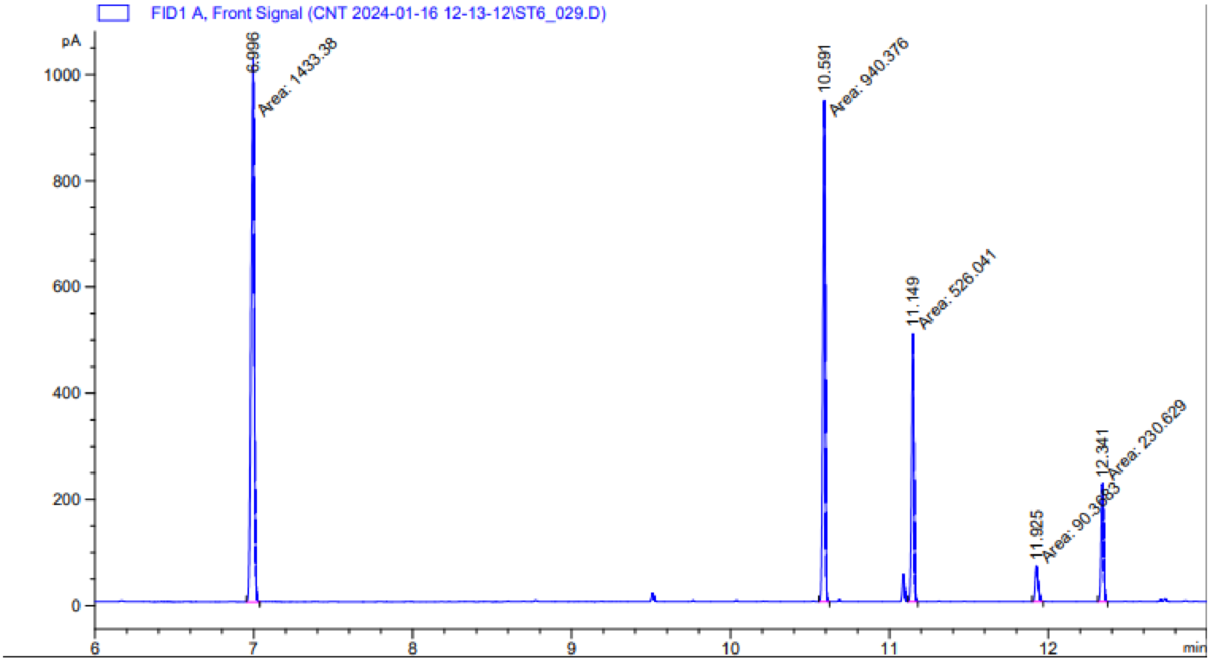

Entry 2

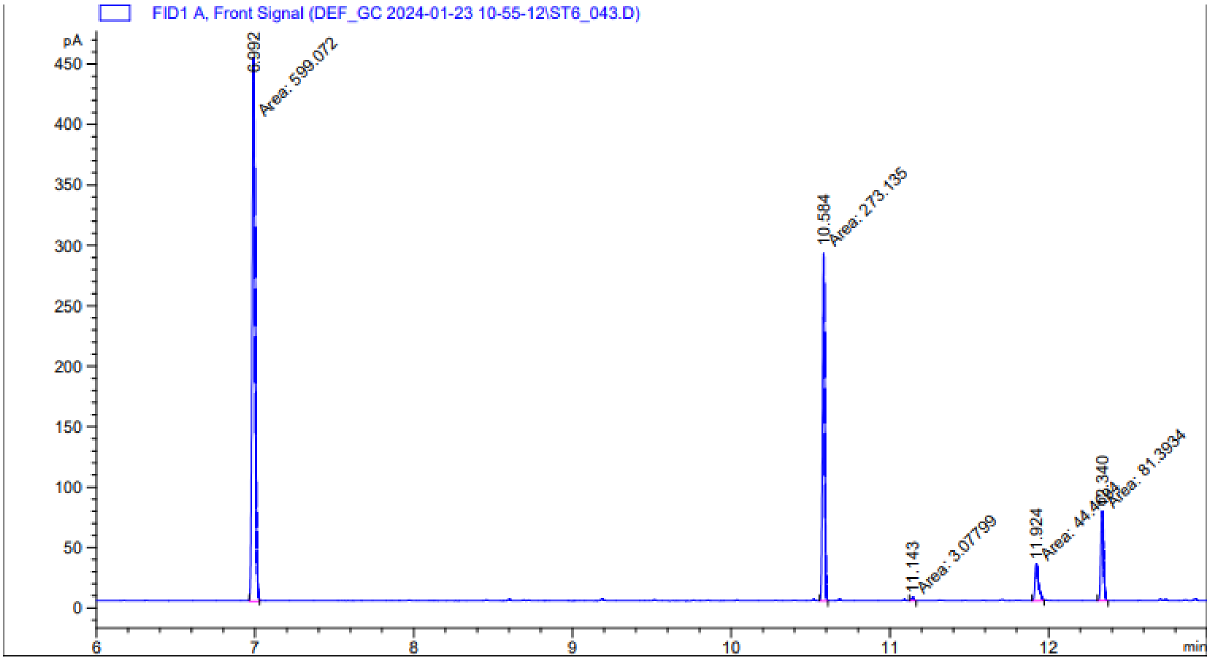

Entry 3

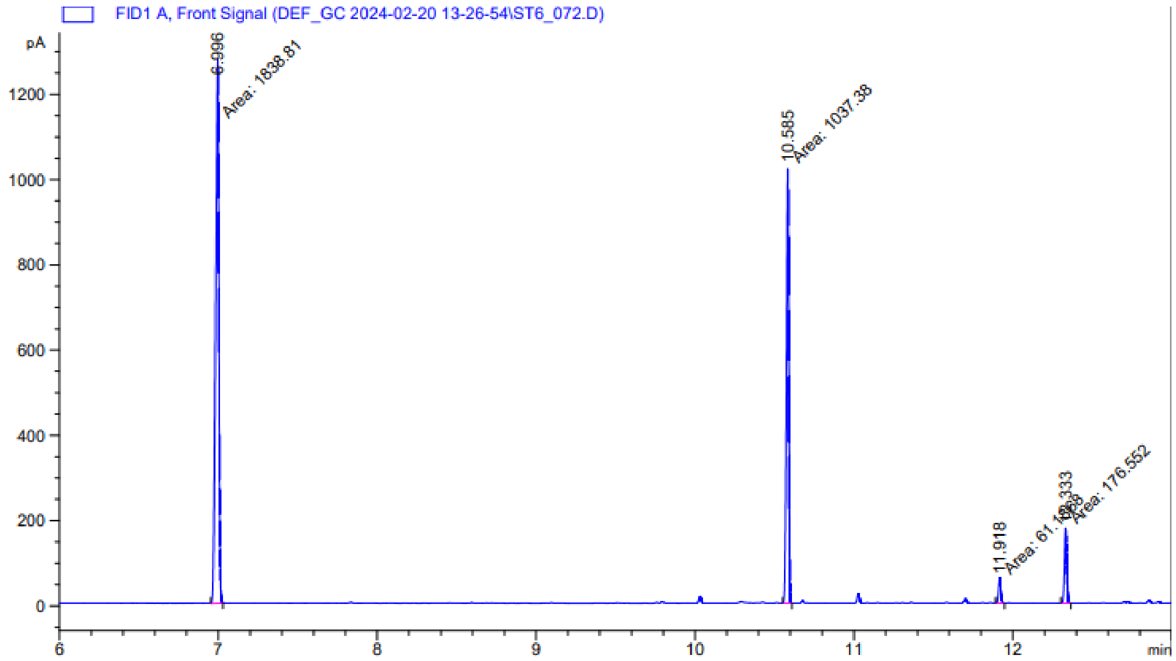

Entry 4

GC-FID traces for Table S6

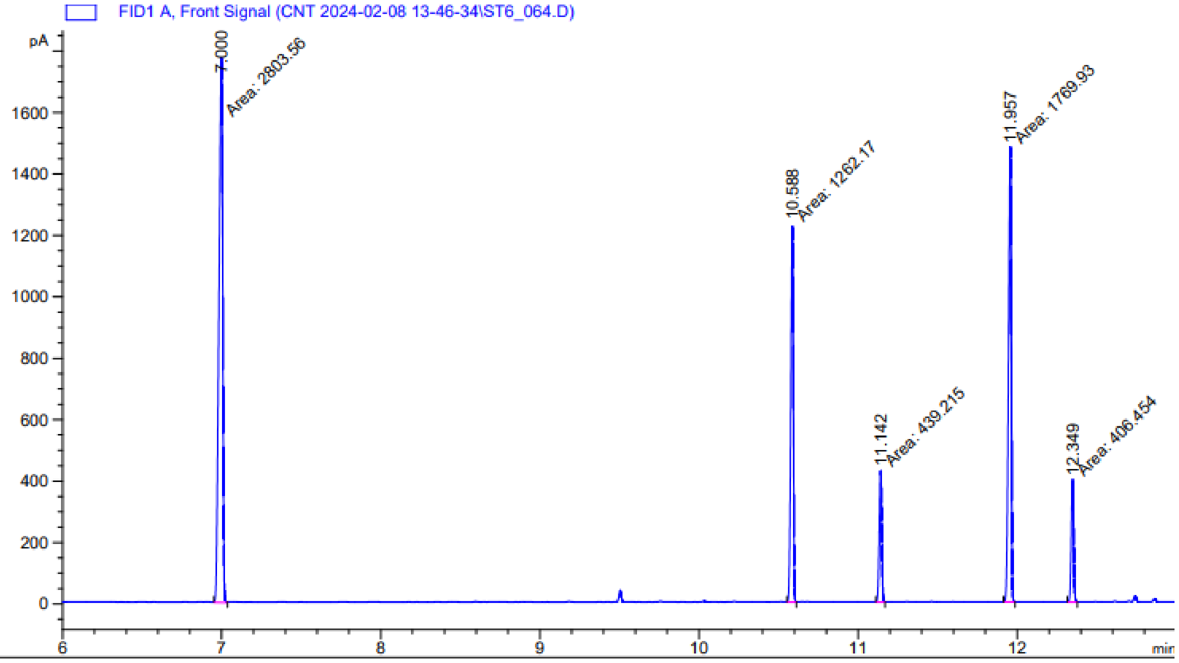

Entry 1

GC-FID traces for Table S7

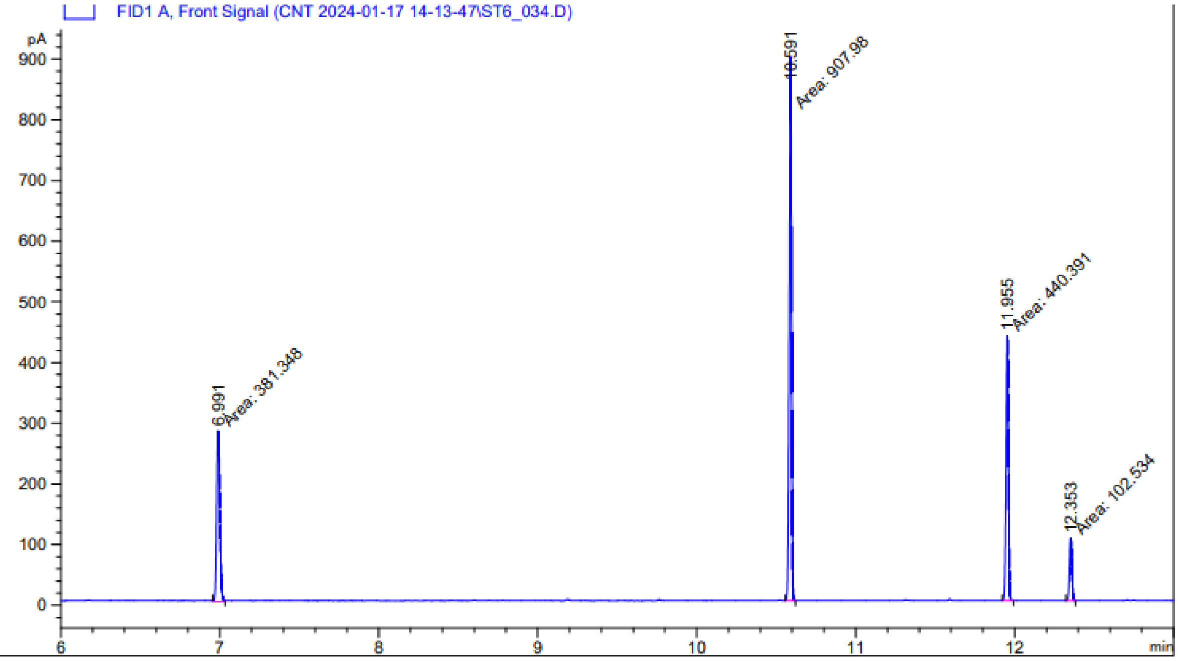

Entry 1

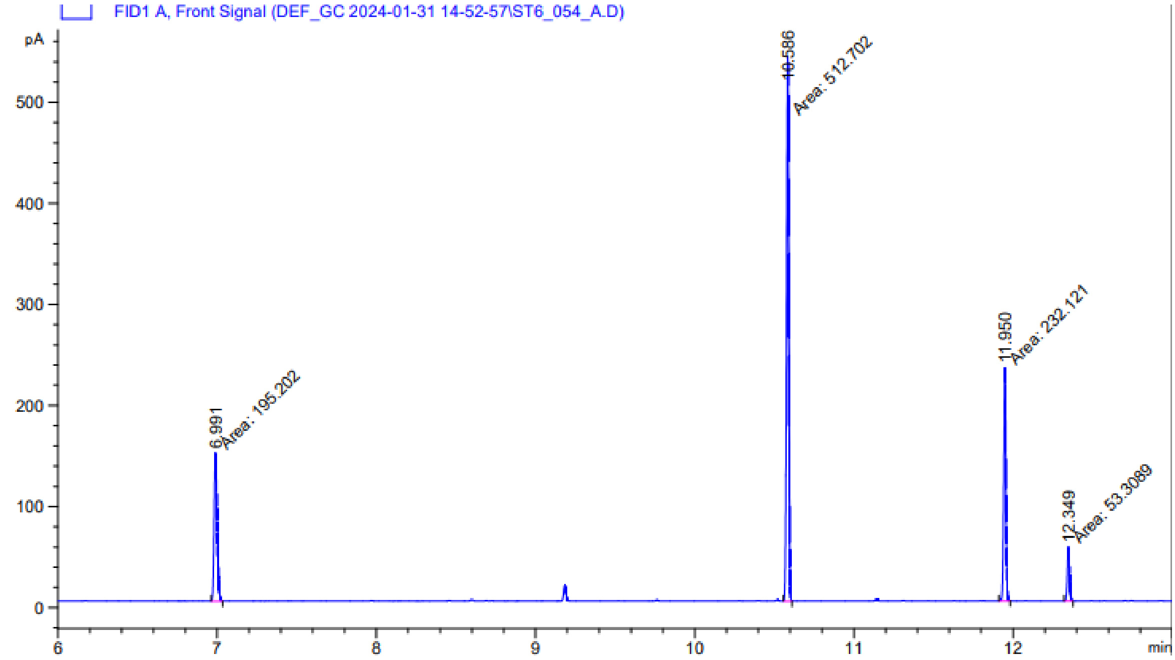

Entry 2

GC-FID traces for Table S8

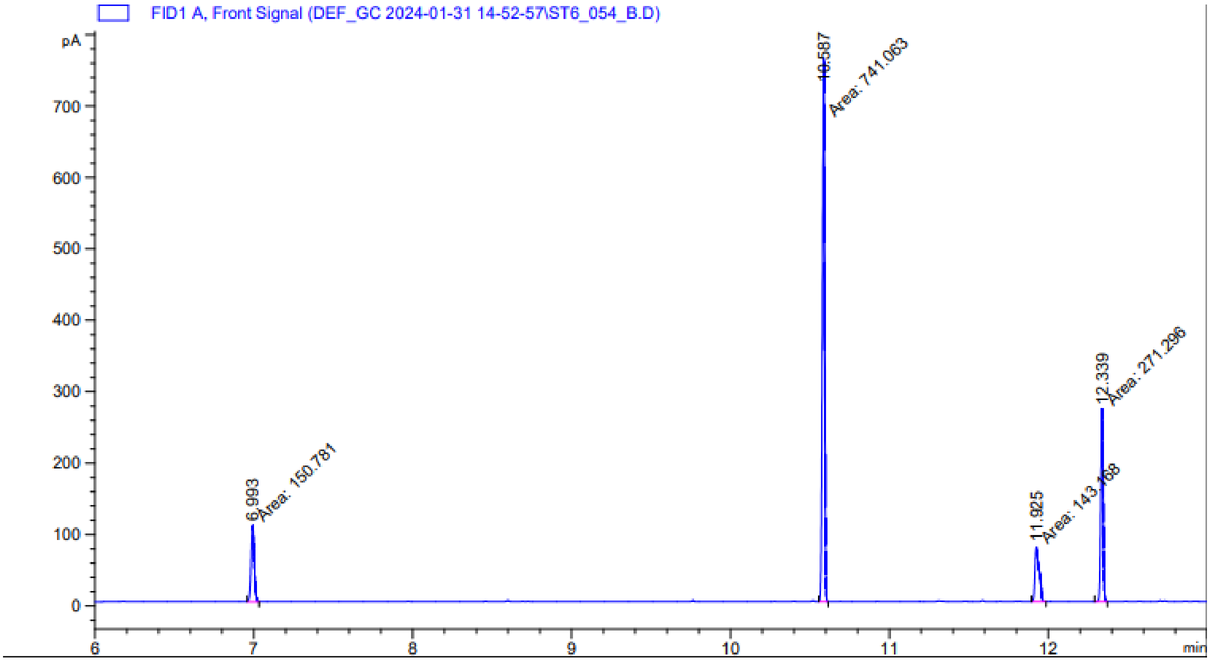

Entry 1

GC-FID traces for Table S9

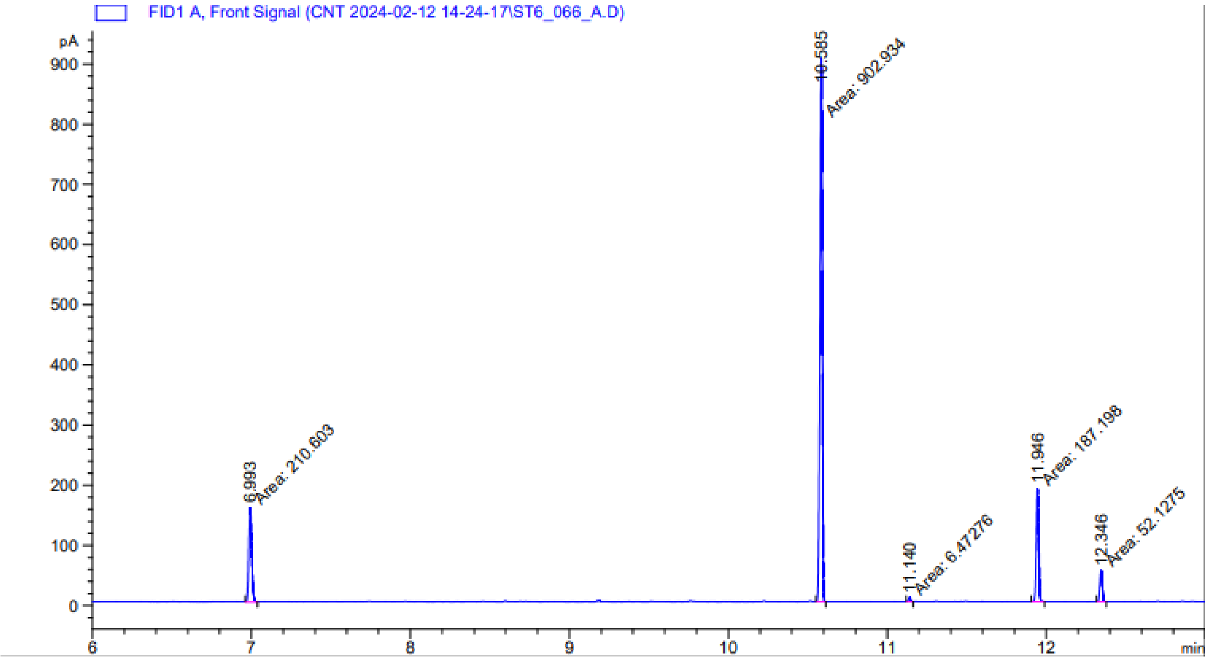

Entry 1

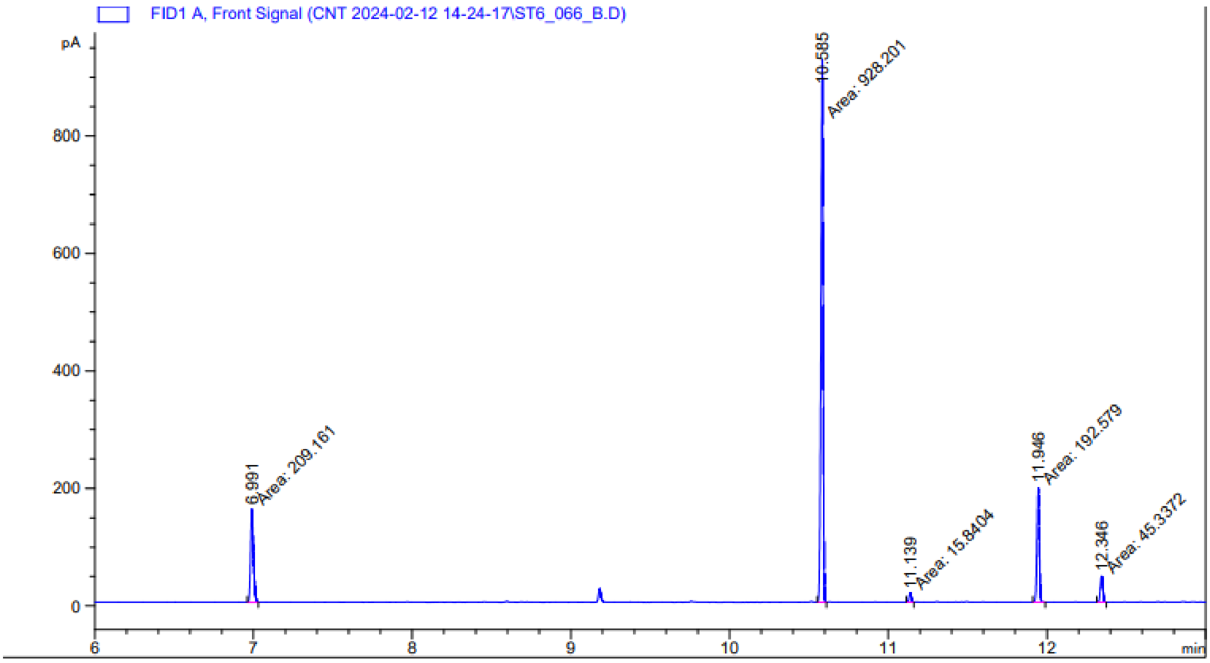

Entry 2

GC-FID traces for Table S10

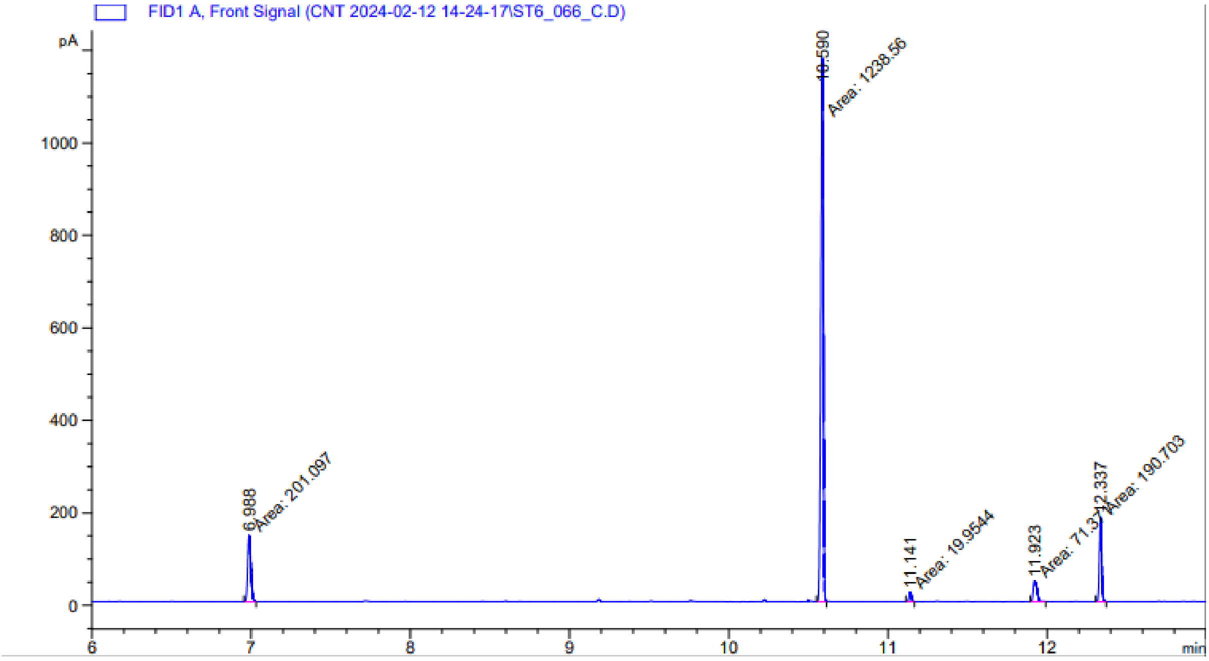

Entry 1

GC-FID traces for Table S11

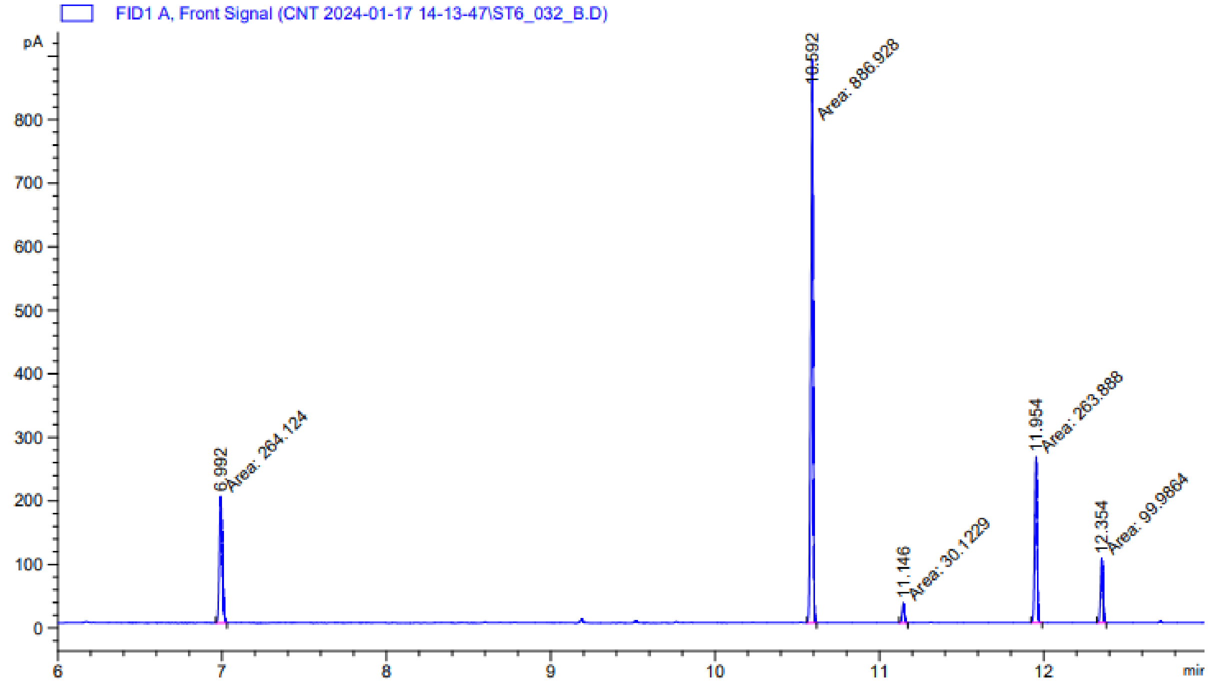

Entry 1

GC-FID traces for Table S12

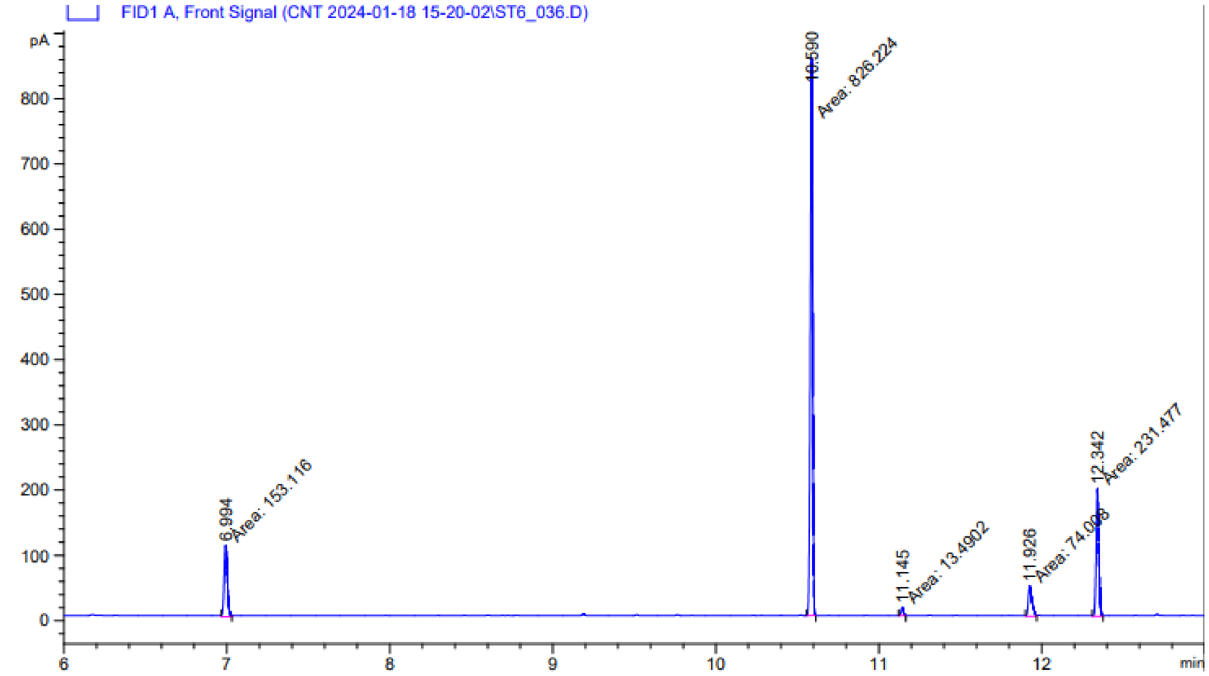

Entry 1

GC-FID traces for Table S13

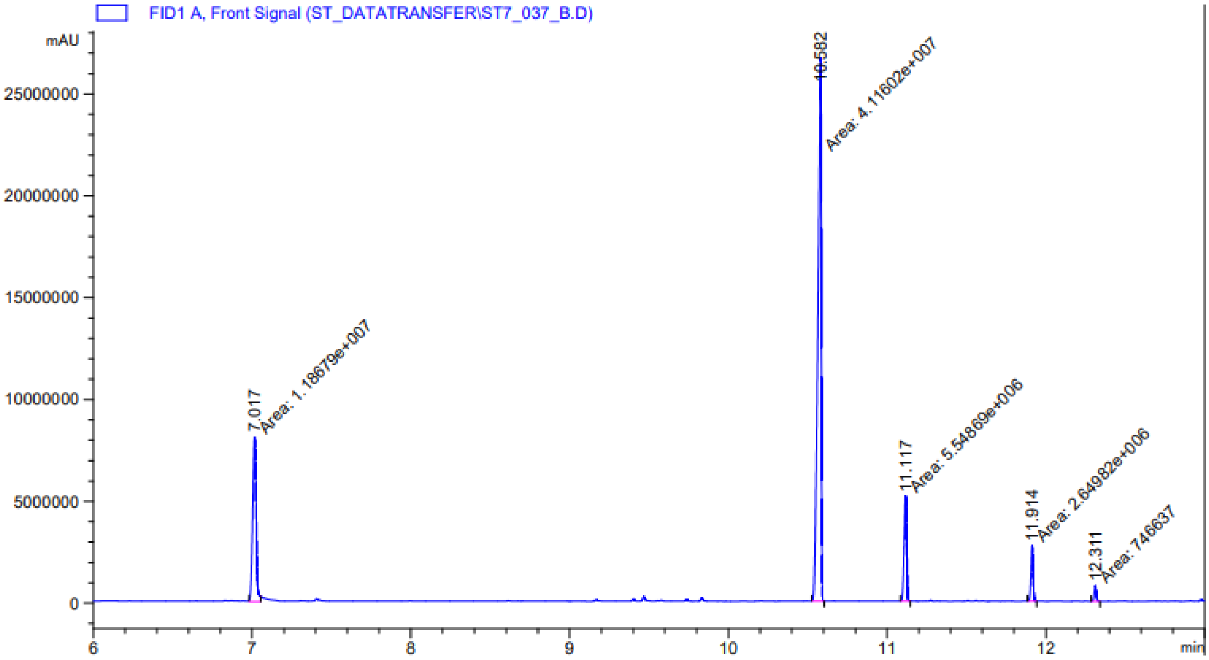

Entry 1

GC-FID traces for Table S14

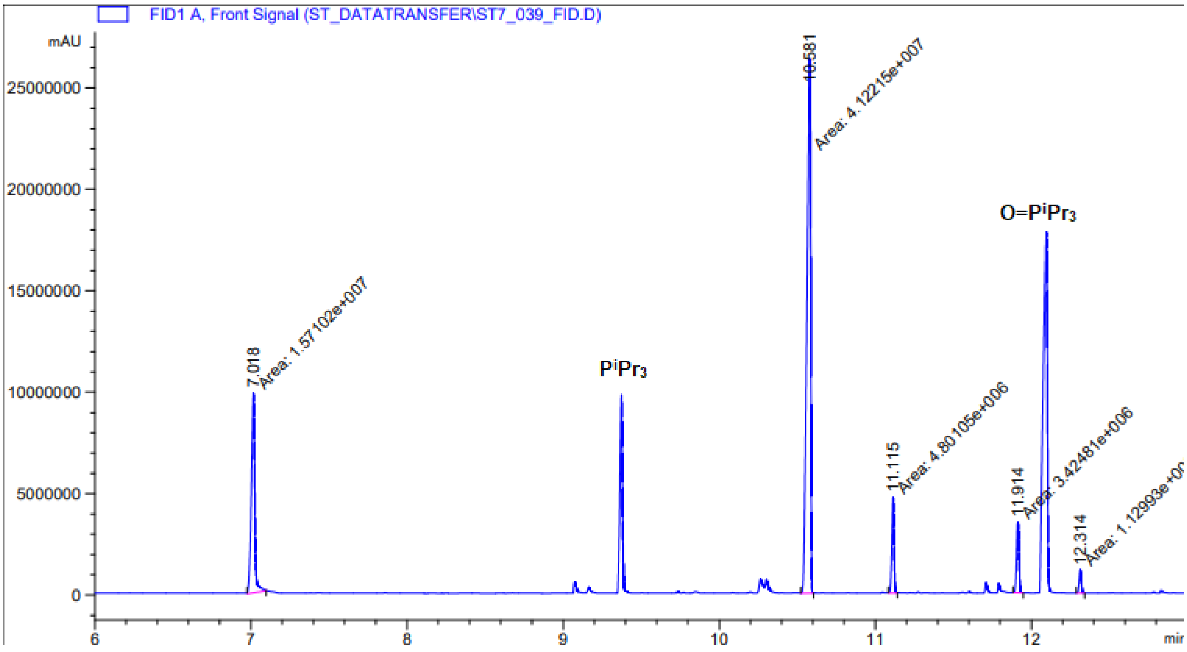

Entry 1

GC-FID traces for Table S15

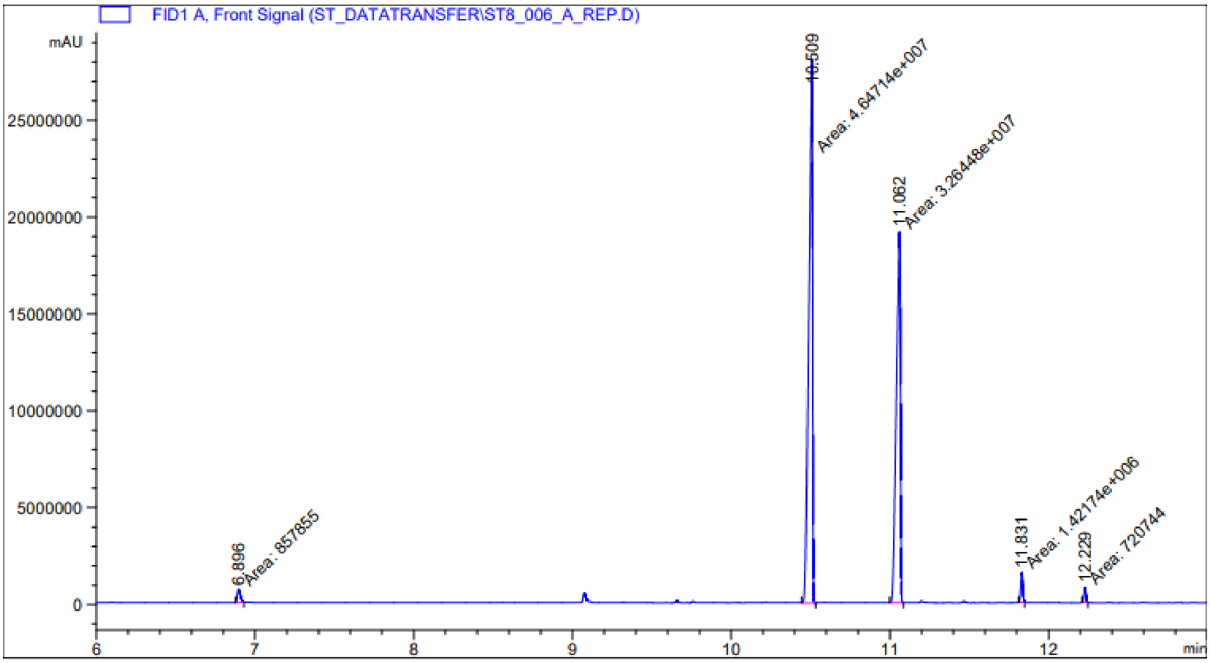

Entry 1

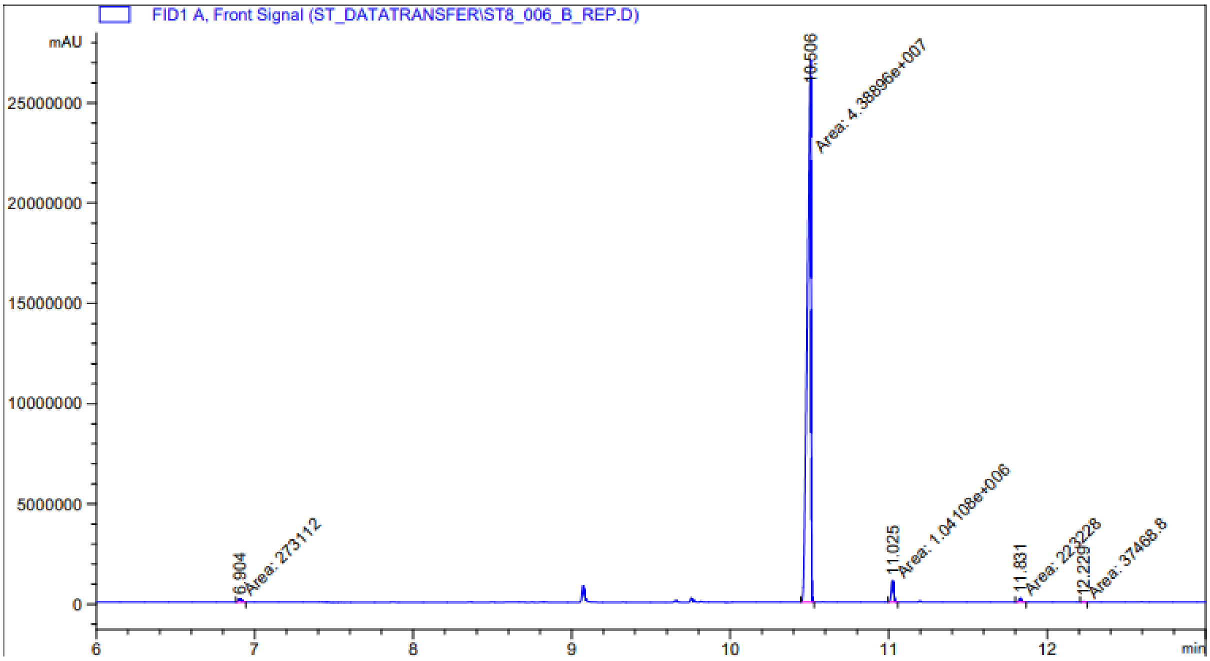

Entry 2

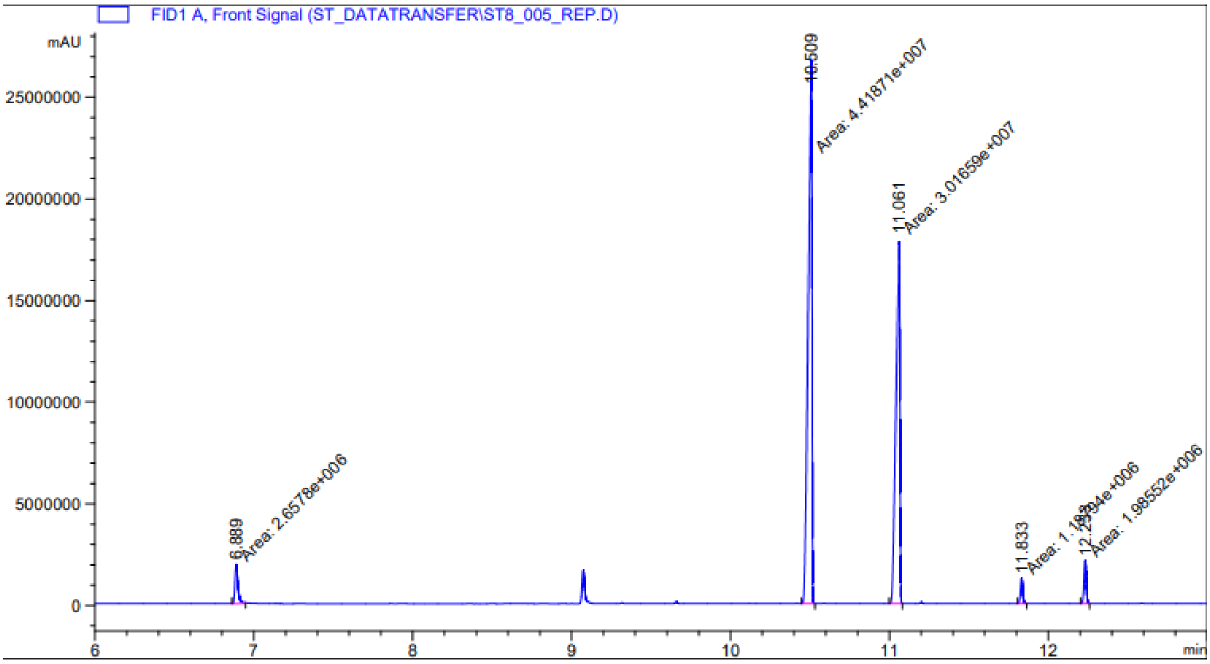

Entry 3

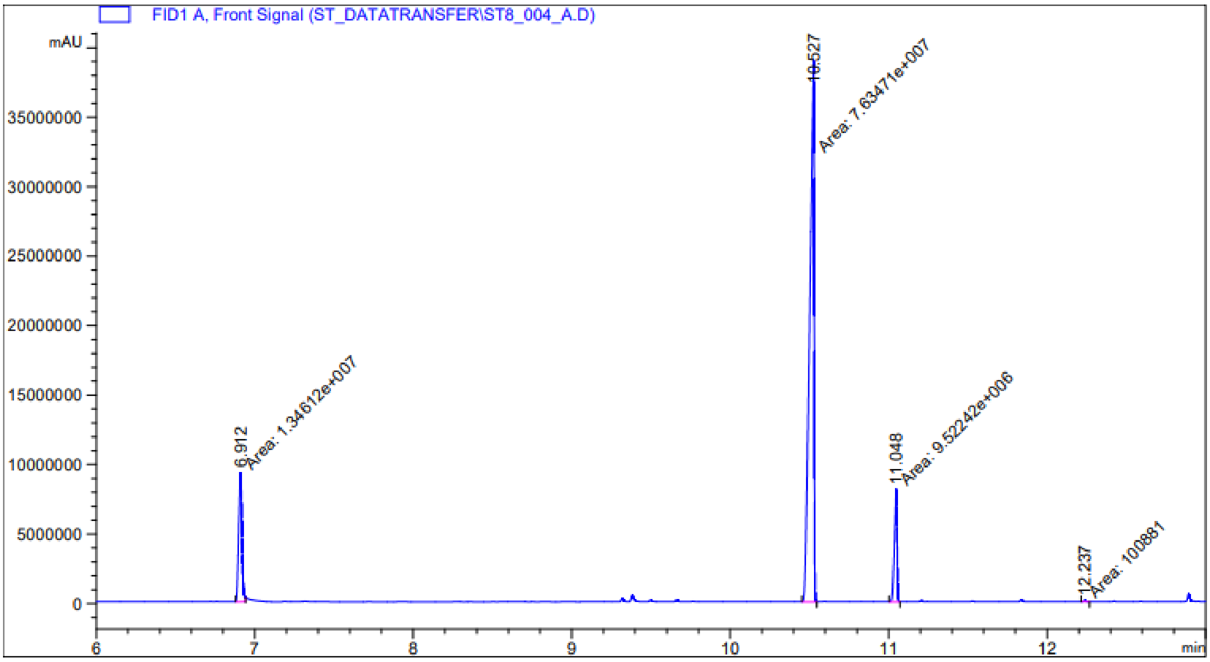

Entry 4

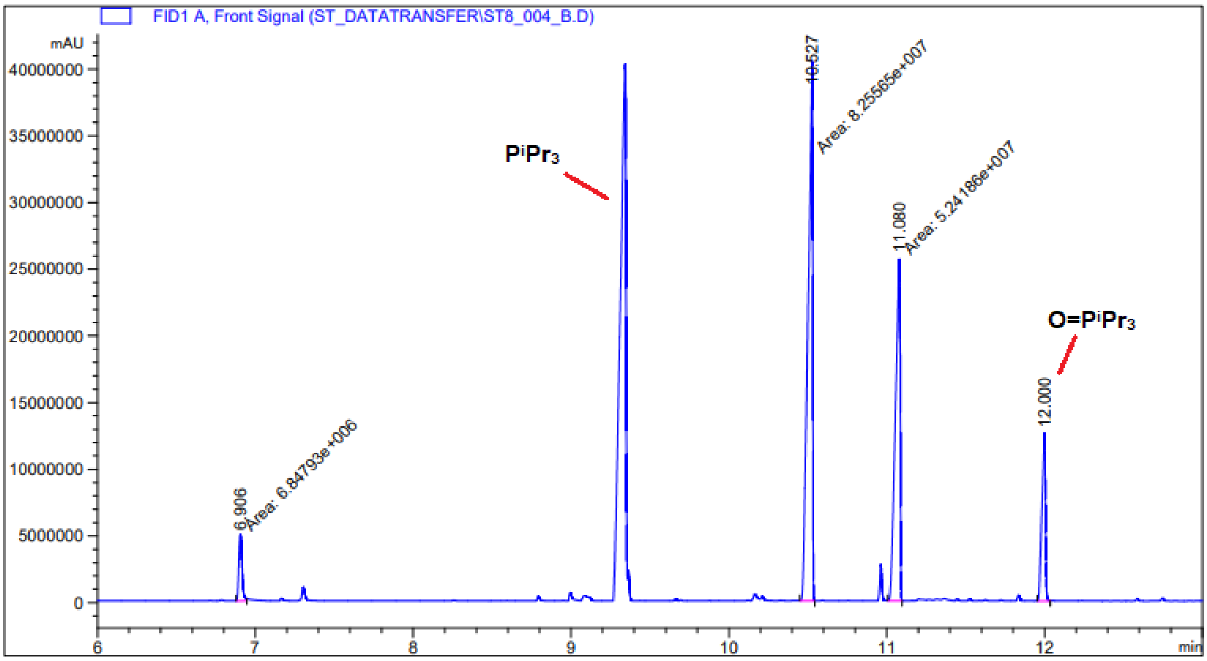

Entry 5 – The signals at 9.3min and 12.0min are P^iPr_3 and OP^iPr_3, respectively, as confirmed by GC-MS. It was also confirmed by GC-MS that no biphenyl or dimethylbiphenyl had been produced in this reaction.

GC-FID traces for Table S17

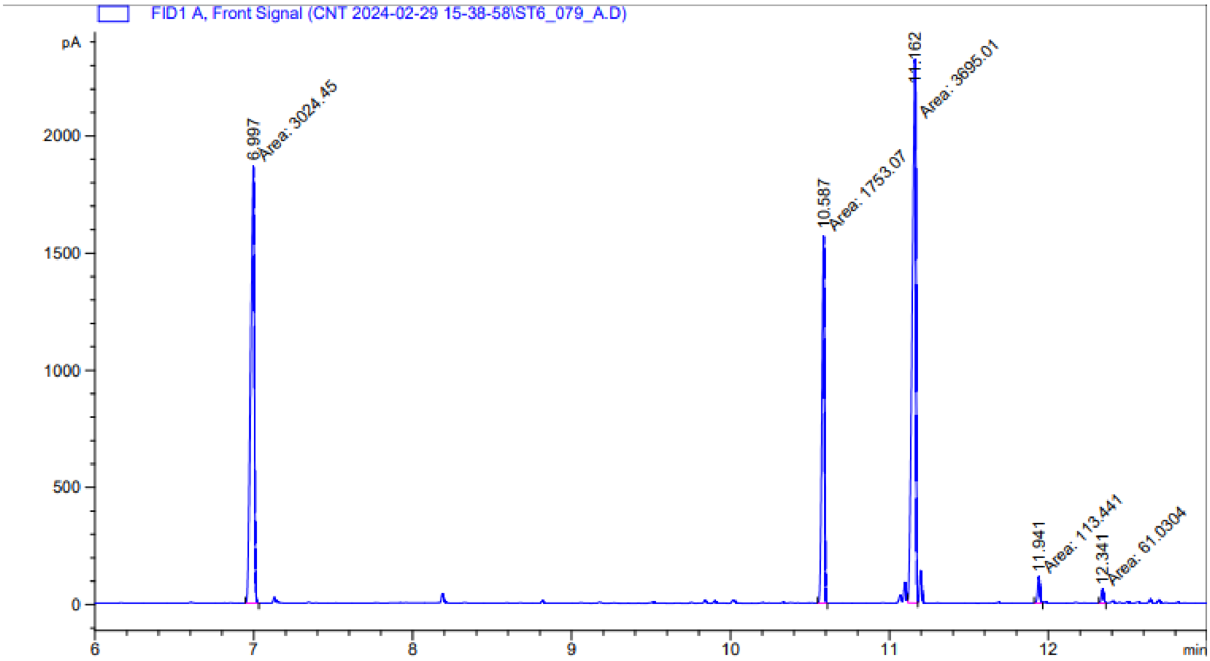

Entry 1

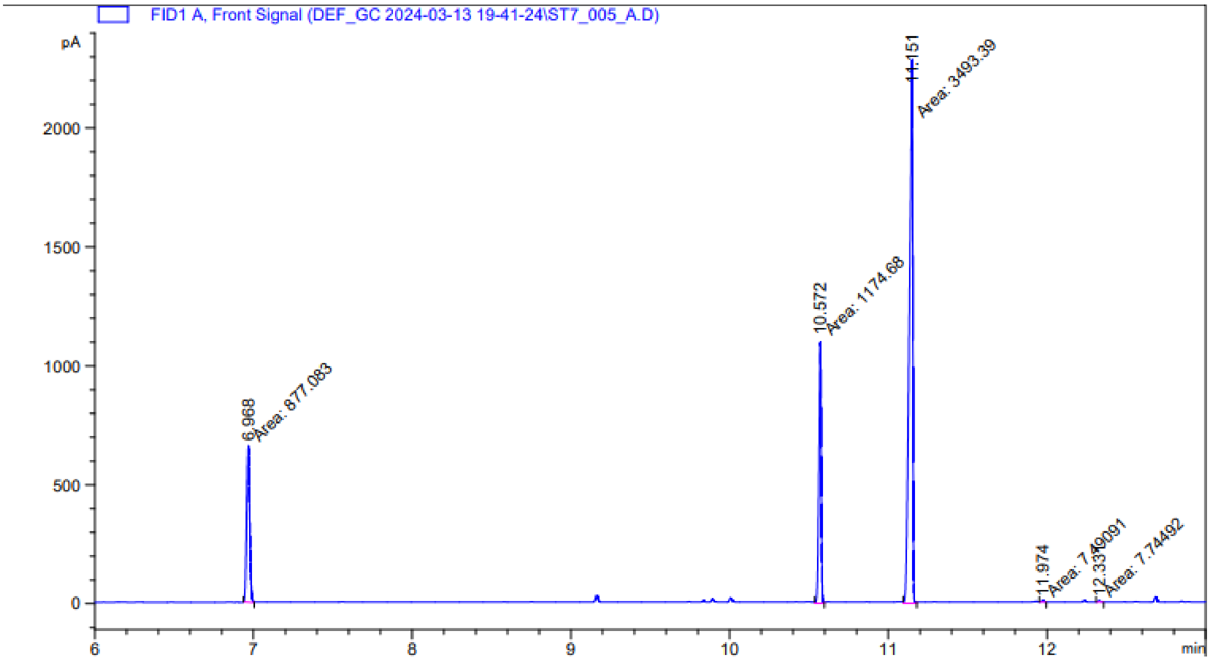

Entry 2

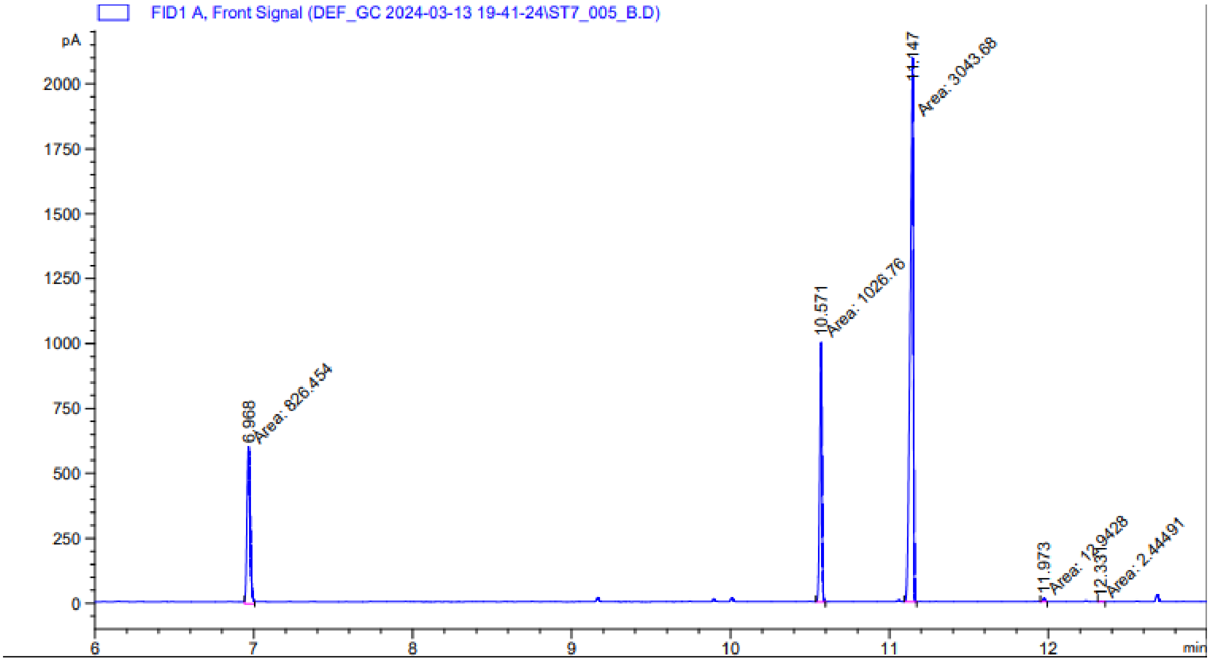

Entry 3

GC-FID traces for Table S18

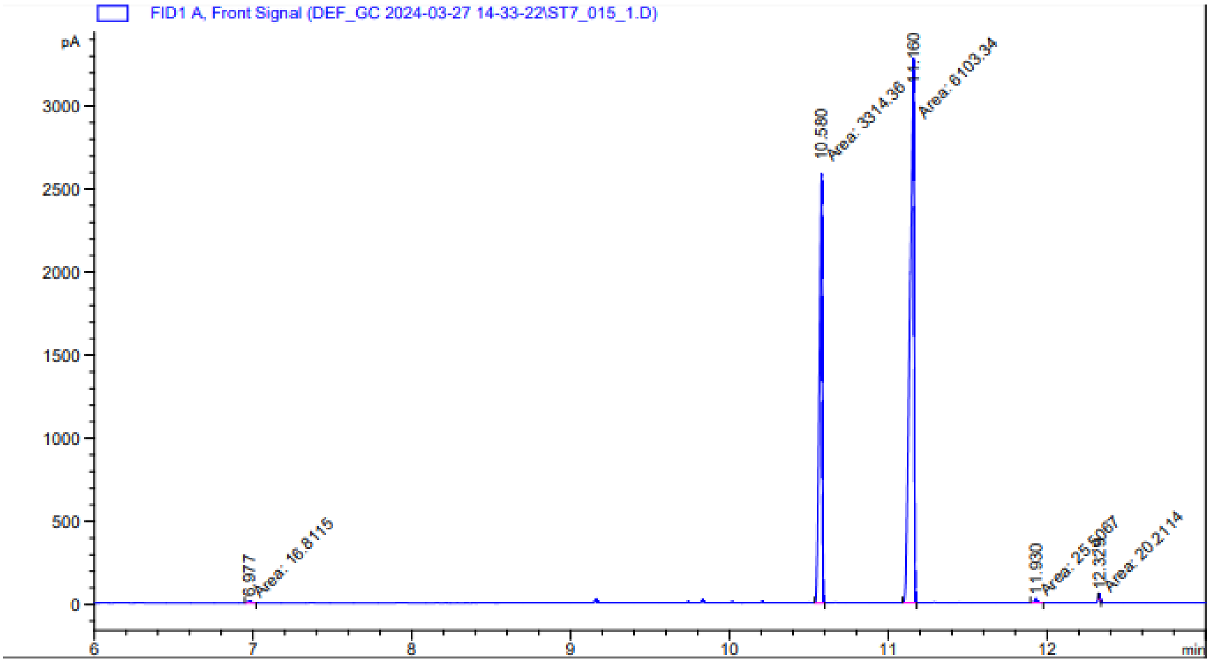

1 hour

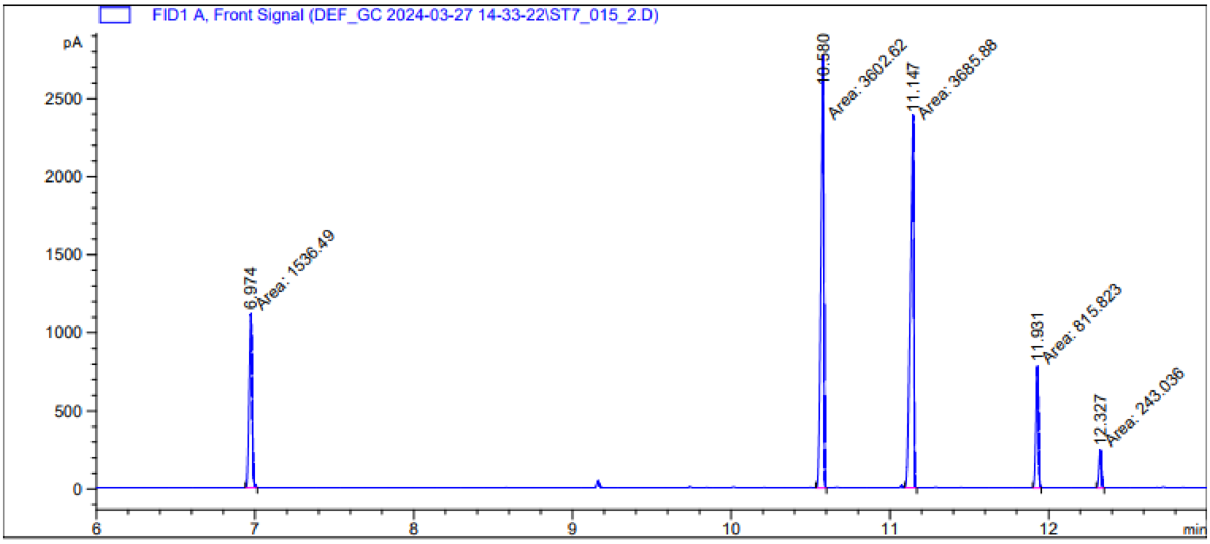

1.5 hours

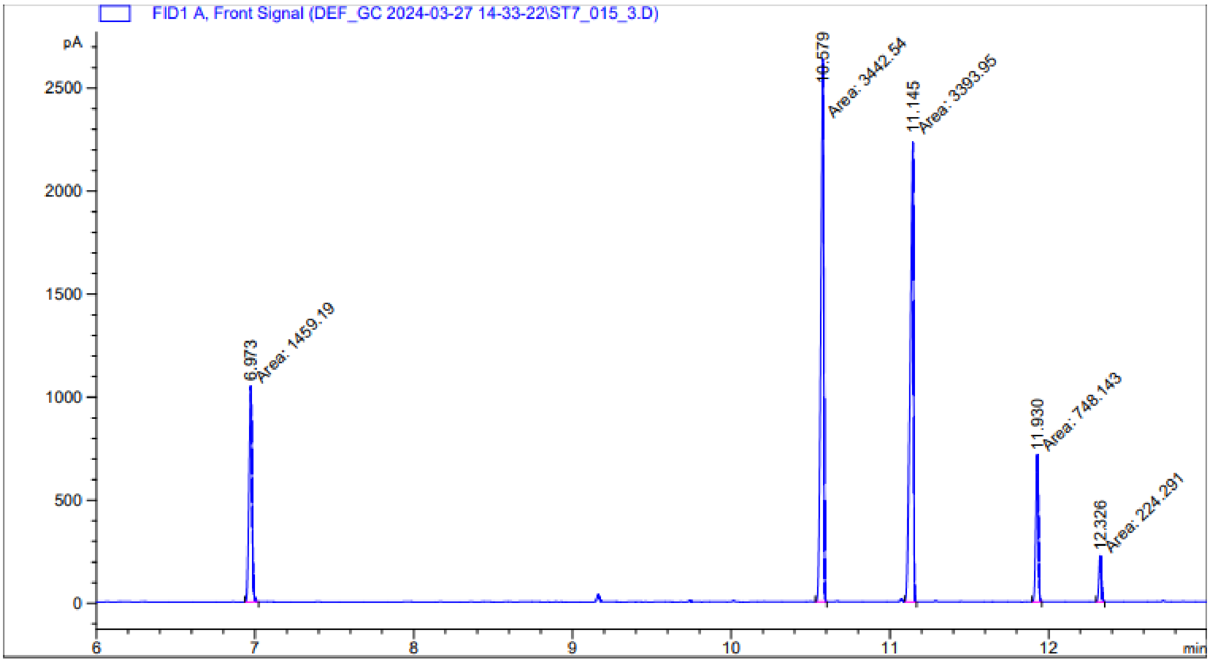

2 hours

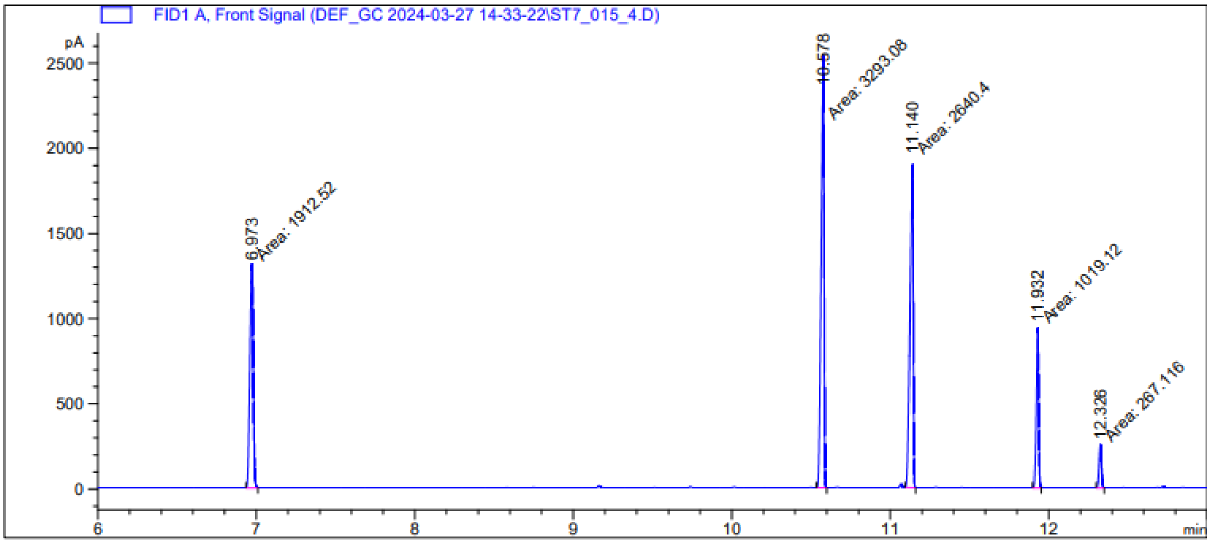

2.75 hours

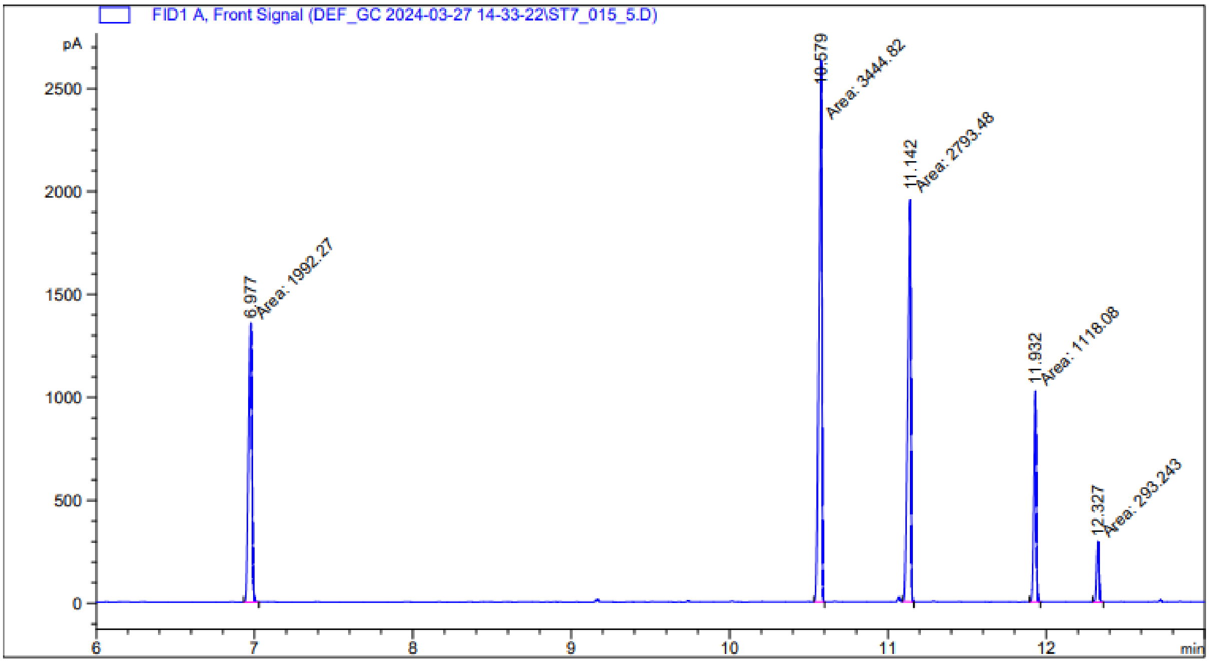

3.5 hours

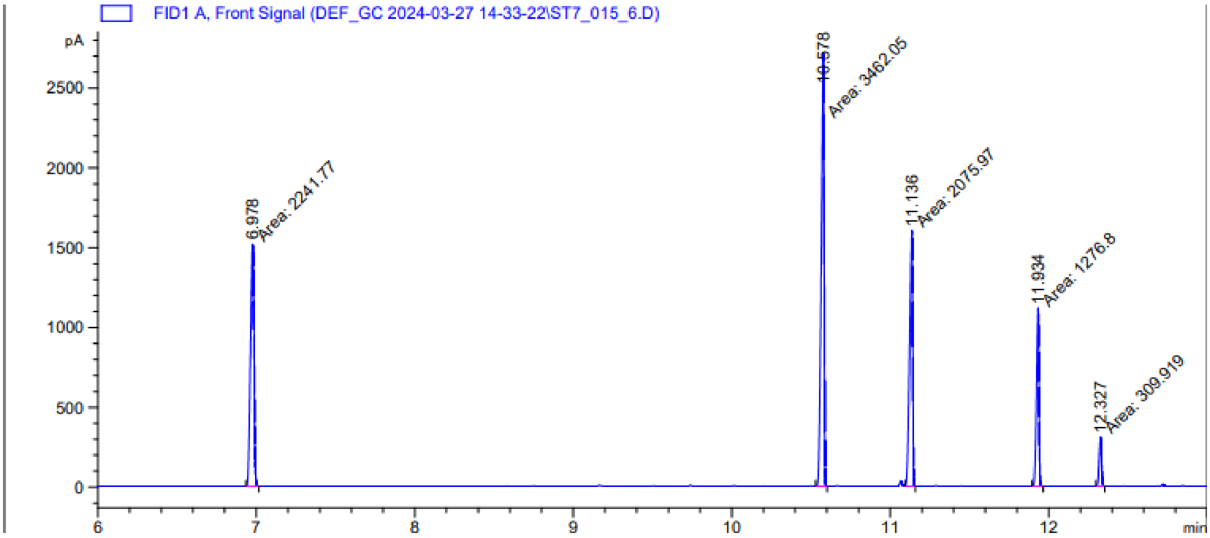

4.25 hours

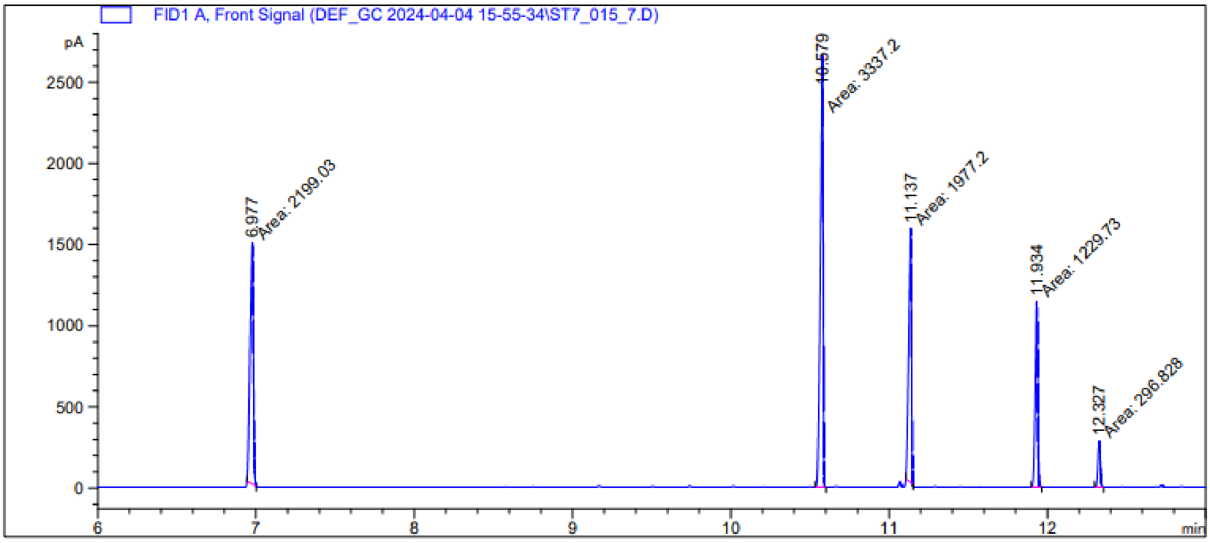

5 hours

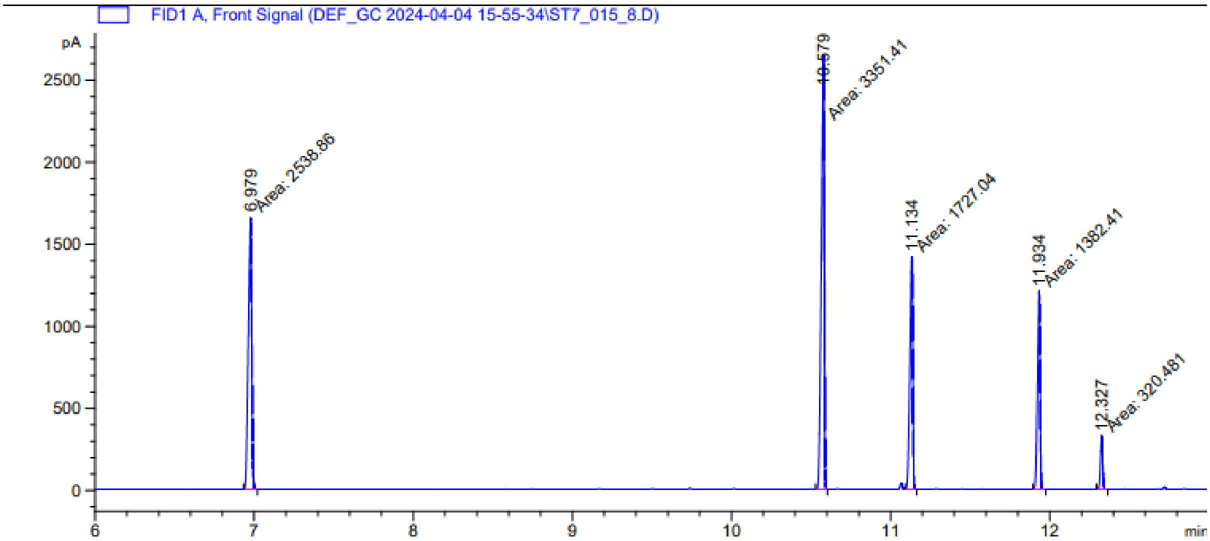

5.75 hours

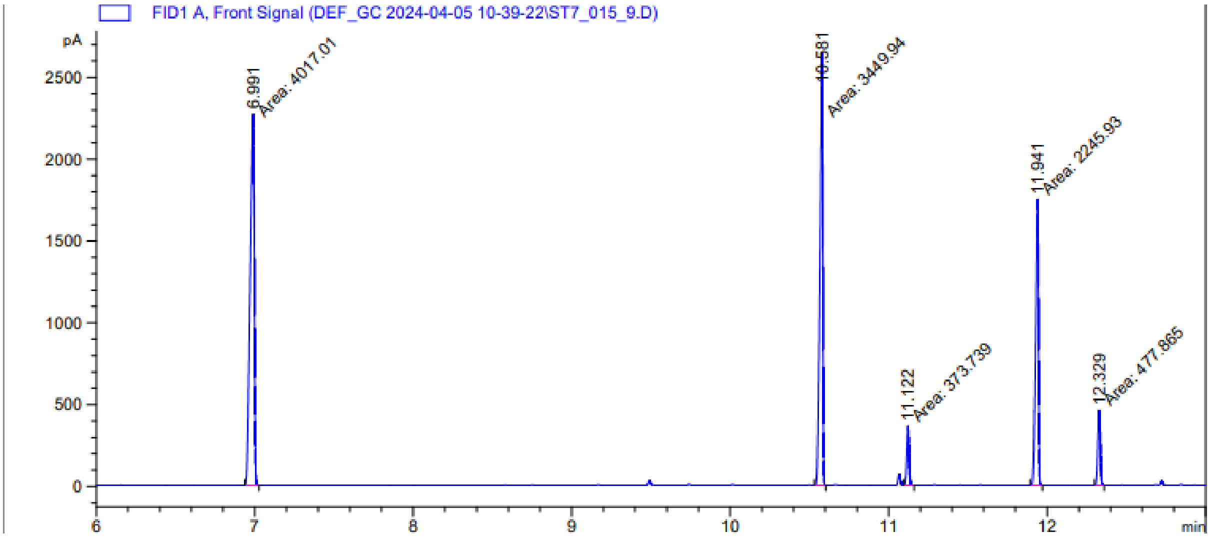

24 hours

17.0 References

(1) Clark, K. F.; Tyerman, S.; Evans, L.; Robertson, C. M.; Nelson, D. J.; Kennedy, A. R.; Murphy, J. A. *J. Am. Chem. Soc.* **2023**, *145*, 20849–20858.

(2) Klingensmith, L. M.; Strieter, E. R.; Barder, T. E.; Buchwald, S. L. *Organometallics* **2006**, *25*, 82–91.

(3) Pérez, H.; Melero, C.; Guijarro, A. ; Yus, M. *Tetrahedron* **2009**, *65*, 10769–10783.

(4) Cismesia, M. A.; Yoon, T. P. *Chem. Sci.*, **2015**,6, 5426–5434.

(5) C. G. Hatchard, C. A. Parker, *Proc. Roy. Soc. A.,* **1956**, 235, 518–536.

(6) a) Kuhn, H. J.; Braslavsky, S. E.; Schmidt, R. *Pure Appl. Chem.,* **2004**, 76, 2105–2146. b) Monalti, M. *et al*., Chemical Actinometry. Handbook of Photochemistry, 3rd Ed; Taylor & Francis Group, LLC. Boca Raton, FL, **2006**, 601–616.

(7) *CrysalisPRO.* **2016**. Rigaku Oxford Diffraction Ltd., Yarnton, England.

(8) Sheldrick, G. M. *Acta Cryst. C* **2015**, *71*, 3–8.

(9) Farrugia, L. J. *J Appl. Cryst.* **2012**, *45*, 849–854.

(10) Tyerman, S.; Robertson, C. M.; Murphy, J. A. *Org. Biomol. Chem.* **2024**, *22*, 1023–1026.
